# Influences of Host Community Characteristics on *Borrelia burgdorferi* Infection Prevalence in Blacklegged Ticks

**DOI:** 10.1371/journal.pone.0167810

**Published:** 2017-01-17

**Authors:** Holly B. Vuong, Grace S. Chiu, Peter E. Smouse, Dina M. Fonseca, Dustin Brisson, Peter J. Morin, Richard S. Ostfeld

**Affiliations:** 1 Rutgers University, Department of Ecology, Evolution, and Natural Resources, New Brunswick, NJ, United States of America; 2 Cary Institute of Ecosystem Studies, 2801 Sharon Turnpike, Millbrook, NY, United States of America; 3 Research School of Finance, Actuarial Studies and Statistics, College of Business and Economics, Building 26C, Australian National University, Canberra, ACT, Australia; 4 Rutgers University, Department of Entomology, 180 Jones Ave., New Brunswick, NJ, United States of America; 5 University of Pennsylvania, Department of Biology, 209 Leidy Laboratories, Philadelphia, PA, United States of America; University of Maryland, College Park, UNITED STATES

## Abstract

Lyme disease is a major vector-borne bacterial disease in the USA. The disease is caused by *Borrelia burgdorferi*, and transmitted among hosts and humans, primarily by blacklegged ticks (*Ixodes scapularis*). The ~25 *B*. *burgdorferi* genotypes, based on genotypic variation of their outer surface protein C (*ospC*), can be phenotypically separated as strains that primarily cause human diseases—human invasive strains (HIS)—or those that rarely do. Additionally, the genotypes are non-randomly associated with host species. The goal of this study was to examine the extent to which phenotypic outcomes of *B*. *burgdorferi* could be explained by the host communities fed upon by blacklegged ticks. In 2006 and 2009, we determined the host community composition based on abundance estimates of the vertebrate hosts, and collected host-seeking nymphal ticks in 2007 and 2010 to determine the *ospC* genotypes within infected ticks. We regressed instances of *B*. *burgdorferi* phenotypes on site-specific characteristics of host communities by constructing Bayesian hierarchical models that properly handled missing data. The models provided quantitative support for the relevance of host composition on Lyme disease risk pertaining to *B*. *burgdorferi* prevalence (i.e. overall nymphal infection prevalence, or NIP_All_) and HIS prevalence among the infected ticks (NIP_HIS_). In each year, NIP_All_ and NIP_HIS_ was found to be associated with host relative abundances and diversity. For mice and chipmunks, the association with NIP_All_ was positive, but tended to be negative with NIP_HIS_ in both years. However, the direction of association between shrew relative abundance with NIP_All_ or NIP_HIS_ differed across the two years. And, diversity (*H'*) had a negative association with NIP_All_, but positive association with NIP_HIS_ in both years. Our analyses highlight that the relationships between the relative abundances of three primary hosts and the community diversity with NIP_All_, and NIP_HIS_, are variable in time and space, and that disease risk inference, based on the role of host community, changes when we examine risk overall or at the phenotypic level. Our discussion focuses on the observed relationships between prevalence and host community characteristics and how they substantiate the ecological understanding of phenotypic Lyme disease risk.

## Introduction

Investigating the ecological factors that influence pathogen genetic variation in a host community may be critical to predicting disease risk. This partly reflects the fact that genetic variants of the pathogen can differ in virulence, transmissibility, and infectivity [1–3]. Unfortunately, our understanding of the ecological drivers influencing pathogen genetic diversity is limited, especially for multi-host zoonotic pathogens.

Interactions of pathogen genotypes with species in the host community may affect the temporal and spatial patterns of genotype prevalence, and could potentially influence the risk of disease [4–8]. For example, human disease severity associated with *Borrelia burgdorferi* [9], *Mycobacterium tuberculosis* [10], *Toxoplasma gondii* [11], and *Helicobacter pylori* [12] varies with different pathogen genotypes. Hence, understanding the ecological interactions between hosts and pathogen genotypic variability could provide insights on ways to reduce disease risk and protect human health.

Here, we examine the Lyme disease system, a disease caused by the bacterium *Borrelia bugdorferi* [13], to advance our understanding of how differences in the host community can influence risk of human exposure. This pathogen replicates within a variety of mammal and bird species and is transmitted between wildlife hosts, and from wildlife to humans, by ticks in the *Ixodes ricinus* complex (*I*. *scapularis*) in eastern North America. Over the past three decades, studies of Lyme disease ecology in the northeastern USA have revealed the importance of small mammals, including the white-footed mouse (*Peromyscus leucopus*), eastern chipmunk (*Tamius striatus*), short-tailed shrew (*Blarina brevicauda*), and masked shrew (*Sorex cinereus*) for general Lyme disease risk [14–18]. These small mammal species are among the most efficient vertebrates at transmitting *B*. *burgdorferi* infection to feeding ticks (i.e. the small mammals are competent reservoirs), and prevalence of tick infection is correlated with absolute or relative abundances of small-mammal hosts [16–19]. The ability of mice, chipmunks, and some shrews, to dominate depauperate faunal communities, by virtue of their ability to respond quickly to environmental degradation [20], appears to contribute to the negative relationship previously detected between host diversity and disease risk [16,18,19,21–24]. Differences in host community composition and potential host-tick feeding interactions might also influence the nymphal infection prevalence and density of infected nymphs.

Lyme disease risk in humans is variable, due to infection by different *B*. *burgdorferi* strains transmitted from nymphal (and adult) ticks, which had previously fed on wildlife hosts that support dissimilar strains of the bacterium [25–28]. The bacterial strains (or genotypes) can be characterized on the basis of their highly polymorphic outer surface protein C (*ospC*). Approximately 25 distinguishable strains of *B*. *burgdorferi* are currently known in the USA, with 17 strains occurring in the northeastern USA alone [29–31]. Of these 17 strains, five (*ospC* types A, B, I, K and N) exhibit significantly elevated occurrence rates among Lyme disease patients [25,32,33]. We collectively termed this subset of five as human invasive strains (HIS). The occurrence of these HIS types in Lyme disease patients warrants exploration of the ecological contributors to variable genotypic frequencies in tick populations associated with various wildlife populations, given that HIS and non-HIS types show divergent frequencies among wildlife hosts [26–28,34].

Our study, administered in 2006 and 2009, was intended to elucidate the determinants of Lyme disease risk, both in terms of overall nymphal infection prevalence (NIP) of any of the strains of *B*. *burgdorferi* (NIP_All_), as well as the prevalence of tick infection with HIS strains (NIP_HIS_) only, across multiple host communities of an endemic county in New York State. We use hierarchical/multilevel Bayesian models to examine NIP_All,_ and NIP_HIS_ simultaneously, the latter conditional on infection, across these host communities. This novel approach captures the variation at the individual tick level, irrespective of whether the ticks were tested positive, negative, or inconclusive for certain HIS strains. The approach also uses site-specific parameters for estimating NIP_All_ and NIP_HIS_. Although we identified strains by their genotypes, our model focused on the phenotypic disease risk (i.e., the prevalence of the HIS category among infected individuals) within the host community as this phenotypic risk is of greater impact than individual strains alone.

## Materials and Methods

### Field Collections

**Fig 1:**
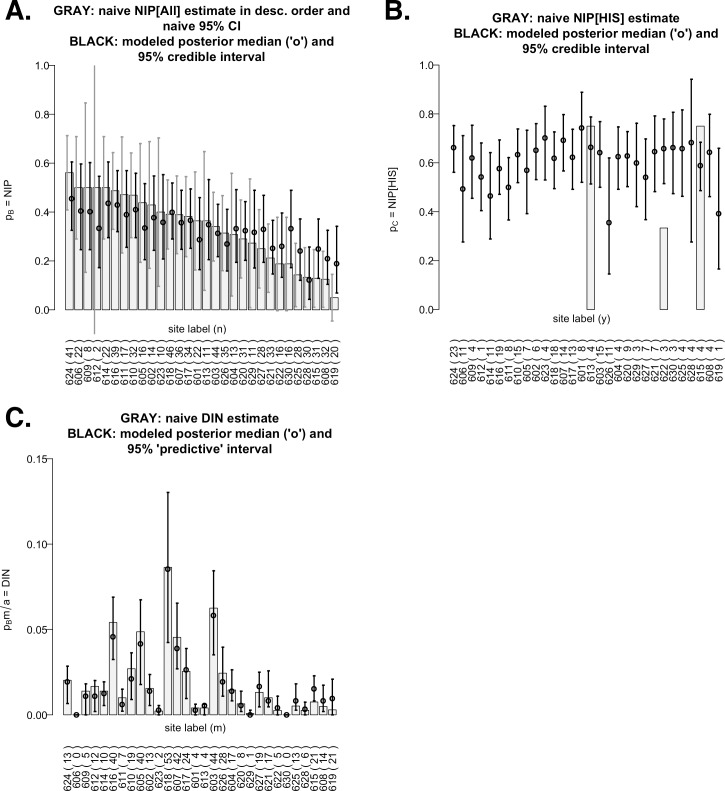
**Graphical displays of 2006 raw data and modeled results of overall nymphal infection prevalence (NIP**_**All**_**; panel A), NIP**_**HIS**_ **(panel B), and overall density of infected nymphs (DIN**_**All**_**; panel C).** All bar charts are ordered by descending values on the *x*-axis. A: Shown as gray bars are the thirty site-specific naïve NIP_ALL_ estimates (= *y/n* where *n* = # of test ticks; *y* = # of ticks which tested positive for *B*. *burgdorferi*) and each corresponding naïve 95% confidence interval (= *y/n* ± 1.96 SE_naïve_(*y/n*) based on sample proportions, also in gray) for the true NIP_All_ (= *p*_*B*_) at that site. In contrast, each black interval is a 95% credible interval (Bayesian confidence interval) using the posterior inference from our Bayesian model. Superimposed on each credible interval is the posterior median (a Bayesian estimate of the site’s true NIP_ALL_). B: Same as panel A but for conditional NIP_HIS_ estimates (= *h/y* where *h* = # of ticks whose RLB procedure indicated HIS+; RLB failure on any of the *y* positive ticks would lead to an indeterminate *h/y*). Only three sites yielded complete RLB results; their naïve confidence intervals were not computed due to small *y*s (hence, an invalid SE formula). In contrast, our Bayesian model provides valid estimates and 95% credible intervals for the true NIP_HIS_ (= *p*_*C*_) for all 30 sites (shown in black). C: Similar to panels A–B but for naïve DIN_All_ estimates (= *[m/a]*x*[y/n]* where *m* = # of nymphs dragged over a distance of *a*) and model-based estimates (= *[m/a]* x [posterior inference for *p*_*B*_]). Due to the uncertainty in *m* (unreplicated and hence, unmodeled), our model-based inference for DIN_All_ here should be interpreted with care (see S4 File).

**Fig 2:**
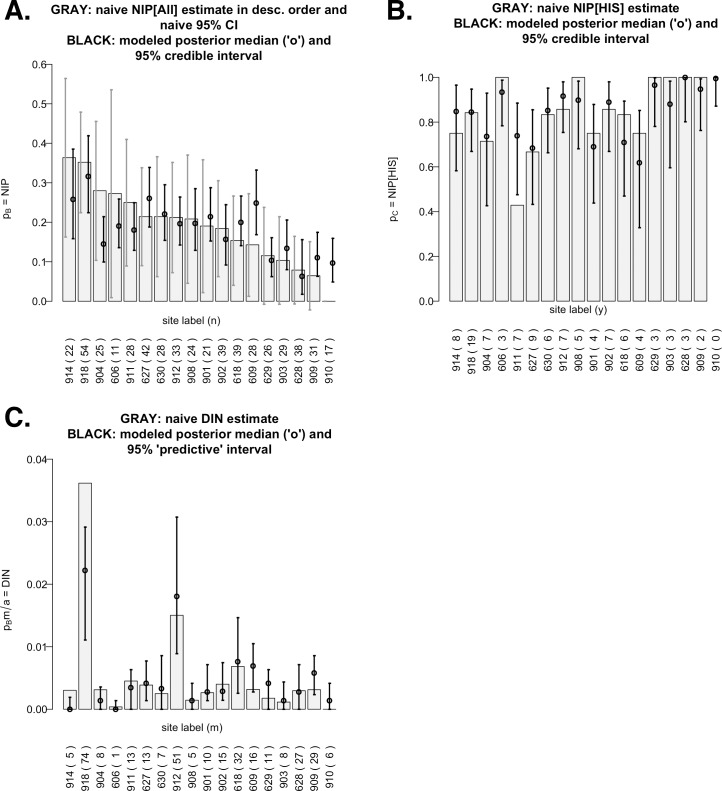
**Graphical displays of 2009 raw data and modeled results of overall nymphal infection prevalence (NIP**_**All**_**; panel A), NIP**_**HIS**_ **(panel B), and overall density of infected nymphs (DIN**_**All**_**; panel C).** All bar charts are ordered by descending values on the *x*-axis. Same information as for Fig 1 (year 2006), but for the eighteen sites in 2009.

We sampled the small mammal communities throughout Dutchess County NY in 2006 (30 sites) and 2009 (19 sites), with seven of the sites sampled in both years (see horizontal axes in Figs 1 and 2). We obtained permission from private land owners to set our grids for the duration of the sampling season on conditions of anonymity. Our trapping dates were 30 May– 19 September 2006 and 2 June– 2 October 2009 [35]. In 2006, we conducted small mammal trapping every other week, whereas in 2009, we trapped at all sites weekly. In both years, each time the site was sampled, traps were deployed for two-consecutive nights (= 1 trapping session). We used an 8 x 8 live trapping grid system, placing one Sherman trap (22.9cm x 7.6cm x 7.6cm) every 15m, and Tomahawks (48.3cm x 15.2cm x 15.2cm) every 30m, for a maximum of 16 Tomahawks and 64 Sherman traps on a full grid (see Supporting Information S1 File for more details). Traps were set between ~15:30 and 17:30, and checked the next morning between 08:30 and 12:00 to avoid any potential heat or cold weather related issues for the animals.

Animals used in this study were approved under the Cary Institute of Ecosystem Studies IACUC numbers 06–03 and 09–01 for field sampling. During the warmer periods of the season, Sherman traps were provided a mix of oats and sunflower seeds for the small animals (e.g. mice, chipmunks). In colder nights, these traps were provided with sunflower seeds and cotton gauze for the animals to create a warm bedding material within the trap. Havahart traps had two raw, unpeeled walnuts for squirrels, and if rain was forecasted, wooden boards were placed over the traps to provide covers for the animals.

Each mammal was identified to species, ear tagged with a unique code, sexed, weighed, evaluated for reproductive status, and then released at the point of capture. Shrews were microchipped with a unique PIT tag rather than an ear tag. Although the trapping occurred throughout the summer, our small mammal diversity measures are based on data from August through early October, coinciding with peak larval tick abundances [36]. Thus, our trapping efforts included four trapping sessions in 2006 and eight trapping sessions in 2009. For the three most common host species captured at each site (white-footed mouse, eastern chipmunk and short-tailed shrew), we calculated the minimum number alive (MNA) using program MARK v.6.0 [37]. MNA is based on mark-recapture data, where individuals are marked upon initial capture and recorded as present or absent in subsequent trapping sessions. We averaged the MNA values across these trapping sessions within each year and used those average values to estimate population densities, based on grid size.

We calculated host Shannon diversity estimates (*H’*) based on the MNA values of the three small mammal host species (white-footed mouse, eastern chipmunk, and short-tailed shrew), avian point counts, and the ‘activity density’ of larger mammalian hosts captured by camera traps. Avian counts were conducted between 05:00 to 10:00 AM to maximize avian detection during early morning activity. These avian counts were conducted two or three times at each site, and birds within a 100 m radius of the observer were identified by sight and sound. We included the American Robin (*Turdus migratorius*), Veery (*Catharus fuscescens*), Ovenbird (*Seiurus aurocapilla*), and Woodthrush (*Hylocichla mustelina*) in our host community estimates for diversity, as these four host species are relevant to tick feeding and *B*. *burgdorferi* infections [18,27,38].

To obtain quasi-quantitative estimates of densities for medium and larger sized mammals, we placed motion-detecting wildlife cameras (DeerCam and CritterGetter) at the sites, with scent lures or raw chicken and corn-cob as bait for two weeks, starting in early October 2006 and mid-October 2009 [16,23]. The number of identifiable individuals in each picture and the number of pictures provide an index of ‘activity level’ for those animals at the site. Briefly, the site with the highest quartile of ‘activity level’ was assigned the ‘most common’ density values, while lower quartile values were scored as ‘present’, and if the animal was absent or rare, the density was recorded as either ‘0’ or some low value, depending on the species. The observed density estimates for each category (most common, present, rare/absent) were based on published values for similar habitats (S2 File). The quasi-Shannon diversity values were based on the most commonly detected species of the host community, following LoGiudice et al. [23] density estimates. The Shannon (*H’*) calculations incorporated values based on ‘activity density’ estimates, averaged weekly minimum number of live densities of mice, chipmunks, and short-tailed shrew, and density estimates of avian hosts.

We collected questing nymphs during the nymphal peak period (June/July) in 2007 and 2010. These questing nymphs represent the previous summer’s larvae that fed on the host community in 2006 and 2009, respectively. At each site, we randomly dragged four 30m transects across our trapping grid to obtain a density estimate of the tick population, followed by a second round of density drags at least two weeks later [39]. To ensure sufficient nymphal sample sizes for estimation of *B*. *burgdorferi* infection prevalence, we conducted additional tick drags on many of the sites, following the second density drags. These supplemental drags were not used for calculations of tick density, and not all ticks collected from the supplemental drags were tested for *B*. *burgdorferi*. Note that one of the 19 sites in 2009 yielded a single nymph despite supplemental drags. Therefore, we omitted this site from consideration, reducing the number of 2009 sites to 18. See S1 File for other details about field collections procedures.

### Lab Analyses

We tested questing nymphal ticks for the *ospC* gene of *B*. *burgdorferi* with a polymerase chain reaction (PCR) procedure, followed by a reverse line blot (RLB) to differentiate the *ospC* genotypes detected [34,40]. For 2006 samples, we used outer primers OC6F/OC623R, followed by inner primers OC6+F/OC602R for a semi-nested PCR. For 2009 samples, we used new outer primers OC-368F/OC693R and new inner primers OC4+F/OC643 for the semi-nested PCR [41]. The primer set used in 2006 had lower binding efficiencies to the probes in the RLB procedure, resulting in only 68.4% of the *B*. *burgdorferi* positive ticks with conclusive *ospC* genotyping results. In contrast, there was 100% efficiency with the 2009 probes, leading to conclusive *ospC* genotyping results for all *B*. *burgdorferi* positive ticks. Genotype *ospC*-C is a hybrid of *ospC*-E and *ospC*-I, making double and triple co-infections with these genotypes difficult to distinguish. We scored *ospC*-C as present when both *ospC*-E and *ospC*-I were present, but we ultimately ignored *ospC*-C for statistical analyses. Genotype *ospC-*J was found once in one year and was absent the other year, so it was also removed from the analyses, resulting in a total of 15 *ospC* genotypes used in the statistical analyses.

### Infection Prevalence Data for Bayesian Analyses

For each site in each year, the lab analysis data were used to calculate the naïve estimates (i.e., simple empirical proportions) of NIP_All_, NIP_HIS_, and DIN_All_ (gray bar charts in Figs 1 and 2). DIN stands for “density of infected nymphs,” defined as the product of NIP and DON (density of nymphs), the latter computed using primary drags only. A naïve NIP_All_ estimate was *y/n*, where *y* was the number of ticks testing positive for any *ospC* type and *n* was the number of ticks subjected to PCR (Figs 1A and 2A). Thus, a naïve DIN_All_ estimate was [*m/a]* x *[y/n]* where *m* was the number of nymphs dragged over a distance of *a* (Figs 1C and 2C). A naïve NIP_HIS_ estimate was *h/y*, where *h* was the number of ticks testing positive for one or more HIS types, although such estimates were missing for most sites in 2006 for which the RLB procedure was inconclusive on one or more *ospC* positive ticks (Figs 1B and 2B).

These naïve estimates of NIP_All_ and DIN_All_ ignored covariate information, which were missing when RLB results were inconclusive, and the naïve confidence interval formula was invalid when the central limit theorem should not be applied, such as when *n* was small. In contrast, our Bayesian models described below integrate auxiliary information (covariates, as well as missing data due to inconclusive RLB), leading to more reliable inference (including credible intervals) that does not require large values of *n*.

### Bayesian Models for NIP_All_ and NIP_HIS_

**Fig 3:**
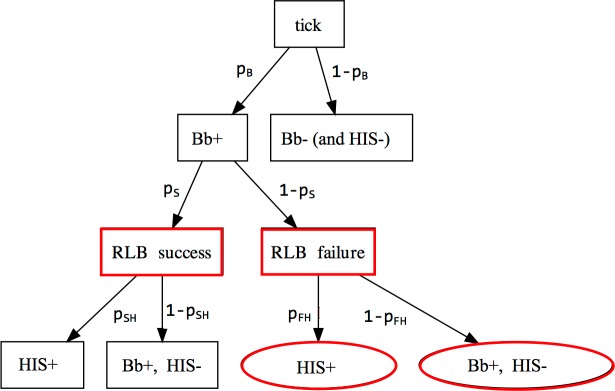
Tree diagram with all possible states and associated probabilities for a test tick. The probabilities are: *p*_*B*_ (nymphal infection rate (NIP_All_) of *B*. *burgdorferi*), *p*_*S*_ (conditional probability of a successful RLB test, given infection), *p*_*SH*_ (conditional probability that the test tick is HIS+, given RLB success), and *p*_*FH*_ (conditional probability that the test tick is HIS+, given RLB failure). Note that *p*_*c*_ (conditional NIP_HIS_, given infection) is equal to *p*_*S*_*p*_*SH*_ + (1 − *p*_*S*_)*p*_*FH*_. Observable states are in boxes, and unobservable states are in ovals. Red nodes do not apply to 2009 because *p*_*S*_ = 1.

**Fig 4:**
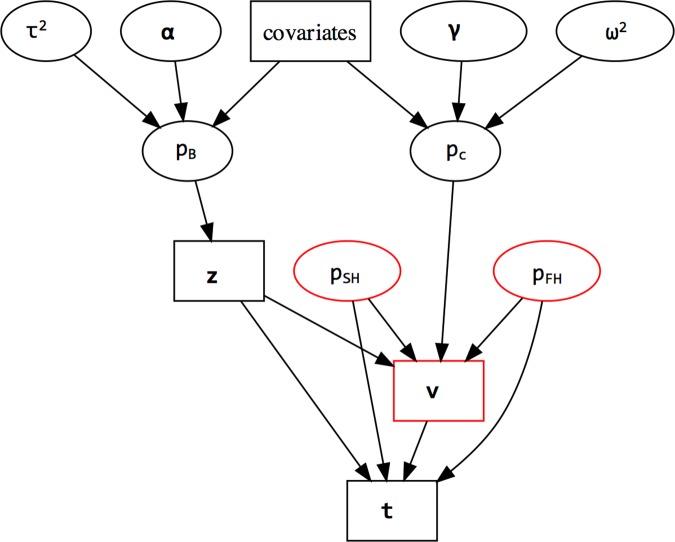
Visual representation of the integrative Bayesian hierarchical approach, upon which our GLM is constructed. All quantities depicted are site-specific except for regression coefficient vectors (***α***,***γ***) and variance parameters (*τ*^2^,*ω*^2^) which are study- (year-) specific. Both *p*_*B*_ and *p*_*C*_ depend on the same covariates. These two sets of dependencies are integrated through (1) the direct collective influence of *p*_*c*_,*p*_*SH*_, and *p*_*FH*_, and ***z*** (vector of 1’s and 0’s denoting the state of *B*. *burgdorferi* infection for test ticks), on ***v*** (vector of 1’s and 0’s denoting success/failure of RLB tests), and (2) the direct collective influence of *p*_*SH*_,*p*_*FH*_, ***z***, and ***v*** on ***t*** (vector of 1’s and 0’s denoting HIS presence/absence on test ticks). Model parameters are in ovals and data are in boxes. Red nodes are not modeled for the 2009 data because ***v*** ≡ **1** (non-stochastic). Model statements and details of the statistical analyses appear in S4 File.

We analyzed the data separately for 2006 and 2009 because most of our sites and the small mammal trapping frequencies differed between years. To examine how host covariates such as *H’* and the relative abundances of white-footed mouse, eastern chipmunk, and short-tailed shrew might influence NIP_All_ and conditional NIP_HIS_ (among infected ticks), we constructed a Bayesian generalized linear model (GLM), first for 2006, then for 2009 (Figs 3 and 4, respectively). Relative abundances for the three small mammal host species were calculated, based on density estimates for the inclusive host community, rather than for just these three specific host species. NIP_All_ and NIP_HIS_ were modeled as site-specific parameters, for which statistical inference was based on binary response variables, using individual ticks as the experimental units; that yielded a total of *n*_*i*_ units (of ticks) at site *i*, along with the associated site-specific covariate values. The dataset was therefore partitioned as such by site, and the *i*^th^ site’s multivariate response was represented by three response vectors (each of length *n*_*i*_): ***z***_*i*_ (containing 1’s and 0’s, denoting observed presence/absence of *B*. *burgdorferi*), ***v***_*i*_ (denoting the success ‘1’ or failure ‘0’ of the RLB test), and ***t***_*i*_ (denoting observed presence ‘1’ or absence ‘0’ of one or more HIS types). We did not distinguish among the five HIS *ospC*-types as we were comparing disease risk phenotypically (HIS vs non-HIS). The reverse line blot (RLB) inefficiencies associated with 2006 samples were taken into account by our models for NIP_All_ and NIP_HIS_. See S3 File for an example of the data associated with one of the sites in 2006. Data from 2009 consisted only of ***z***_*i*_ and ***t***_*i*_ vectors, because *ospC*-detection was complete for the 2009 samples.

Our integrative Bayesian framework fully accounted for the non-stochastic nature of the HIS presence/absence data (*t*_*ij*_) when *B*. *burgdorferi* was absent (*z*_*ij*_ = 0), and for the unobservability of *t*_*ij*_ when the RLB procedure failed (*v*_*ij*_ = 0). (See boldfaces in S3 File.) This is achieved by defining Bernoulli distribution parameters that correspond to Fig 3, namely, *p*_*B*_ (nymphal infection prevalence (NIP_All_) of *B*. *burgdorferi*), *p*_*c*_ (conditional NIP_HIS_, given infection), *p*_*S*_ (conditional probability of a successful RLB test, given infection), *p*_*SH*_ (conditional probability that the test tick is HIS+, given RLB success), and *p*_*FH*_ (conditional probability that the test tick is HIS+, given RLB failure). The relationships depicted by Fig 3 induce the constraint that $p_{i}^{S}=(1-p_{i}^{c}/p_{i}^{FH})(1-p_{i}^{SH}/p_{i}^{FH}{)}^{-1}$, which justified our choice of prior distributions (see S4 File). The regression equations of the GLM are

$$\log\frac{p_{i}^{B}}{1-p_{i}^{B}}=\alpha_{0}+\alpha_{1}x_{1i}+\cdots +\alpha_{4}x_{4i}+\eta_{i},$$

$$\log\frac{p_{i}^{c}}{1-p_{i}^{c}}=\gamma_{0}+\gamma_{1}x_{1i}+\cdots +\gamma_{4}x_{4i}+\xi_{i},$$

where *x*_*ki*_ denotes the *k*^th^ covariate (i.e., the relative abundance of mouse, shrew, or chipmunk species, or *H’*), and where the residual terms *η*_*i*_ and *ξ*_*i*_ have zero-mean Gaussian (normal) distributions with respective variance parameters *τ*^2^ and *ω*^2^. The ***α***,***γ*** regression coefficients vectors and the residual variance parameters (*τ*^2^ and *ω*^2^) were modeled to follow vague Gaussian and vague inverse-Gamma prior distributions, respectively, reflecting our lack of *a priori* knowledge concerning the behavior of these parameters. Additionally, there was no *a priori* indication that RLB test failure could be associated with a tick's underlying infection state, so by assuming a common RLB failure rate, irrespective of infection status, our modeling strategy allowed us to impute missing values of *t*_*ij*_ by treating them as unobserved model parameters. The inference for $p_{i}^{c}$ in turn accounted for these imputed values (Fig 4). Note that the covariates were log-transformed to reduce skewness (S5 File), then subsequently centered to improve computational efficiency ([42–44] and S5 File). See S4 File for the roles of model parameters, observed data, and prior and posterior distributions in Bayesian inference, and for detailed model statements for our studies.

The integrative models simultaneously accounted for variation in the number of tested and infected ticks, over- or under-dispersion (as most ticks were not infected with *B*. *burgdorferi*, there was an elevated number of ‘0’ counts, leading to a distribution that was not a true binomial), as well as influential site-specific data points. This framework offers a flexible analytical approach to identifying relevant covariates of NIP_All_ and NIP_HIS_, as it utilizes all information available from the dataset. Standard logistic regression techniques would handle the two types of prevalence in separate analyses, while ignoring information on RLB efficiency, but our hierarchical Bayesian models utilize the RLB efficiency as a linkage between the two types of prevalence data, improving our overall statistical inference.

We also assessed our Bayesian model’s goodness-of-fit in various ways, one of which was posterior predictive checks (e.g. [45]) which we describe as follows (see S4 File for more detail). We considered the naïve NIP_All_, NIP_HIS_, and DIN_All_ estimates and compared them to what our Bayesian models predicted. Specifically, we used the posterior inference from our models to make predictions of PCR results, (hence, posterior predictions of *y*), and computed the predictive versions of the naïve NIP_All_ and DIN_All_ estimates by using the model predictions. Goodness-of-fit of our models would be deemed high if each of the NIP and DIN estimates were consistent between raw data and posterior predictive results.

## Results

### *ospC* Infection

In 2006, 167 of 245 (68.2%) tick samples hybridized efficiently with specific probes in the reverse line blots, whereas all 103 samples amplified from 2009 hybridized with the probes efficiently. Thus, *ospC* analyses for 2006 were based only on samples for which hybridization was successful. Because we used different primer sets for the 2006 and 2009 tick samples, we also tested whether primer changes could account for the changes in relative proportions of the *ospC* types detected each year. The proportions of each *ospC* genotype in the two years were marginally correlated (*r* = 0.49, df = 13, *p* = 0.06). While year to year variation is likely, it is also possible that there may have been a small potential bias in primer binding to *B*. *burgdorferi*, or that the PCR products bound differentially in the reverse line blot. We note that the mean number of genotypes per tick was smaller in 2006 (2.02, SE = 0.12) than in 2009 (2.41, SE = 0.18) but not significantly so (Wilcoxon W = 7704, *p* = 0.07). On balance, we concluded that separate analyses of NIP_All_ and NIP_HIS_ prevalence between years were justified.

### Exploratory Analyses of Prevalence Data from Bayesian Modeling

Our Bayesian model-based inferences for NIPs (*p*_*B*_,*p*_*c*_) appear in black in Fig 1A and 1B for 2006 and Fig 2A and 2B for 2009. Bayesian inference is based on posterior distributions, which can be summarized by posterior medians (shown as circles) and 95% credible intervals (shown as black intervals). The intervals are interpreted as follows: given the data and model, there is (a) a 0.5 probability that the true NIP is larger than its posterior median, and (b) a 0.95 probability that the true NIP lies inside the shown credible interval. The model-based credible intervals are not only tighter (hence, the inference has more power) than the naïve confidence intervals (shown as gray intervals), but are also valid even when very few or no ticks were tested (e.g. sites 612 in 2006 and 910 in 2009) or when RLB data were missing (e.g. all but 3 sites in 2006). In Figs 1C and 2C, we took the product between the posterior median of NIP_All_ and the naïve DON (density of nymphs) to produce the posterior medians for DIN_All_ (density of infected nymphs); they can be regarded as model-based estimates of DIN_All_, but they and the accompanying predictive intervals are interpreted differently than the model-based medians and intervals for NIPs (see S4 File).

Referring to Figs 1 and 2, we see that naïve NIP_All_ estimates ranged from ~0.05 to 0.55 in 2006, whereas they ranged from 0 to ~0.35 in 2009. Their corresponding naïve 95% confidence intervals (in gray) are superimposed on the bar charts (and will be discussed further below). For NIP_HIS_, of the three sites with conclusive RLB results in 2006, two had naïve estimates near 0.8. Conclusive RLB testing in 2009 allowed calculation of naïve NIP_HIS_ estimates for all sites except one (site 910), due to the absence of positive ticks in the sample. In most 2009 cases, the naïve NIP_HIS_ estimate was quite high, being ~ ≥ 0.7 at 16 of the 18 observed sites. For DIN_All_, naïve estimates were noticeably higher in 2006 than in 2009, preliminarily suggesting higher Lyme disease risk for the 2006 sites than for the 2009 sites.

### Bayesian Inference for Host Influence on Disease Prevalence

Naïve NIP and DIN estimates were consistent between using raw data and using model predictions. Because of this cross-validation and other model diagnostics (S4 File), the goodness-of-fit of our Bayesian models was deemed high, thus we proceed to summarize the modeling results.

With respect to the relationship between NIPs and host-community characteristics, our Bayesian modeling results show that for 2006, integrating RLB methodological failure with detection of infection and HIS improved the inference for model parameters associated with HIS status, namely $p_{i}^{c}$ (the conditional probability that a tick from the *i*^*th*^ site would test positive for HIS, given infection), *γ* and *ω*^*2*^ (the vector of regression coefficients and residual variance, respectively, when predicting HIS status from covariates). Improvement amounts to a reduction in estimation uncertainty (reduced standard deviation of the posterior distribution), relative to models that ignore such failure. Not modeling RLB failure would amount to collapsing the tree diagram in Fig 3 by removing boxes in red and combining the red ovals with their respective box-shaped counterparts in black, which also removes the red nodes in Fig 4. Moreover, modeling RLB failure alongside NIP_All_ and NIP_HIS_ provided valuable inference on $p_{i}^{S}$ (the conditional probability of RLB success for a tick from the *i*^*th*^ site, given infection), $p_{i}^{SH}$ (the conditional probability that a tick from the *i*^*th*^ site would test positive for HIS, given RLB success), and $p_{i}^{FH}$ (the conditional probability that a tick from the *i*^*th*^ site, given RLB failure, would have tested positive for HIS, had RLB been successful), none of which would have been possible with the collapsed model (S4 File).

Based on the integrated model, the posterior probability for a regression slope parameter (*α* or *γ*) to take on a positive/negative value can be interpreted as evidence that the corresponding host community abundance is positively/negatively related to disease prevalence. For example, the regression slope estimate of +0.52 and a posterior probability of 0.96 that *α*_2_ > 0 in Table 1 (second row, year 2006), can be interpreted as near certainty that the relative abundance of mice is positively associated with NIP_All_. Unlike classical hypothesis testing, a Bayesian posterior probability is literally the ‘probability of the scenario in question’ as informed by the observed data.

**Table 1:** Main results of the integrative logistic regression analyses.

			2006		2009	
	Covariate	Parameter	Estimate	Posterior Probability	Estimate	Posterior Probability
	H’ (Host Shannon-Weiner Div)	*α*_1_	-0.04	0.57	-0.41	0.70
	Mouse Relative Abundance	*α*_2_	+0.52	0.96	+0.66	1.00
**NIP_ALL_**	Chipmunk Relative Abundance	*α*_3_	+0.10	0.89	—	—
	Shrew Relative Abundance	*α*_4_	+0.13	0.90	-0.10	0.71
	H’ (Host Shannon-Weiner Div)	*γ*_1_	+0.20	0.74	+1.67	0.71
	Mouse Relative Abundance	*γ*_2_	-0.73	0.96	-1.93	0.90
**NIP_HIS_**	Chipmunk Relative Abundance	*γ*_3_	-0.06	0.70	-0.81	0.98
	Shrew Relative Abundance	*γ*_4_	+0.26	0.95	-0.57	0.89

Estimates (i.e. posterior medians) of regression coefficients and posterior probabilities of positive or negative association between covariates and NIP_All_ or NIP_HIS_. A high posterior probability implies a high degree of confidence (little uncertainty) in the direction of the estimated association. For example, the second row under 2006 indicates a posterior probability of 0.96 (very high confidence) that mouse relative abundance is positively associated with NIP_All_ (slope estimate = +0.52). Missing entries correspond to a covariate that was omitted from our final 2009 model fit because there was negligible evidence of its association with NIP_All_ in the preliminary models that included all four covariates.

From our modeling, we found that each of the mouse, chipmunk, and shrew relative abundances showed good evidence of a positive relationship with NIP_All_ in 2006 (slope estimates = 0.52, 0.10, and 0.13, respectively; posterior probabilities = 96%, 89%, and 90%, respectively) and *H’* showed mild evidence of a negative relationship with NIP_All_ (slope estimate = -0.04; post. prob. = 57%) (Table 1). For 2009, only the collapsed model is required, due to fully observed ***z***_*i*_- and ***t***_*i*_-vectors. Our results showed very strong evidence that mouse relative abundance was again positively associated with NIP_All_ (slope estimate = 0.66; post. prob. > 99%), whereas *H’* and shrew relative abundance showed some evidence of negative association with NIP_All_ (slope estimates = -0.41 and -0.10, respectively; post. probs. = 70% and 71%, respectively) and the influence of chipmunk relative abundance was negligible.

For 2006, conditional NIP_HIS_ was negatively associated with mouse and chipmunk relative abundances, although the strength of evidence differed between host species (Table 1; slope estimates = -0.73 and -0.06, respectively; post. probs. = 96% and 70%, respectively). However, *H’*, and shrew relative abundance, were positively associated with NIP_HIS_ (slope estimates = 0.20 and 0.26, respectively; post. probs. = 74% and 95%, respectively). In contrast, the relative abundances of mice, chipmunks, and shrews in 2009 each showed strong evidence of negative association (slope estimates = -1.93, -0.81, and -0.57, respectively; post. probs. = 90%, 98%, and 89%, respectively). As in 2006, *H’* was positively associated with NIP_HIS_, with a similar level of evidence (slope estimate = 1.67; post. prob. = 71%). (See S6 File for more detail on model fit and an in-depth discussion of nuances of our statistical analyses with respect to disease detection practices.)

## Discussion

Our study examined how host community covariates can affect overall *B*. *burgdorferi* nymphal (tick) infection prevalence (NIP_All_), and infection prevalence of human-invasive strains (NIP_HIS_), the latter conditioned on ticks being infected. We were interested in understanding how the relative abundances of white-footed mouse, eastern chipmunk, and short-tailed shrew, and overall host community diversity (*H’*), might affect Lyme disease risk. This study assessed the influence–if any–of host community composition (i.e. relative abundances of three primary hosts), and of *H’* (which is a combination of species richness and evenness) on NIP_All_ and NIP_HIS_. Our model provides, as quantitative evidence, the probability that NIP_All_ and NIP_HIS_ were indeed associated with the relative abundances of particular hosts and/or host diversity. Overall, our analyses highlight that the relationships between the relative abundances of three primary hosts and the community diversity with NIP_All_, and NIP_HIS_, are variable in time and space, and that disease risk inference, based on the role of host community, changes when we examine risk overall or at the phenotypic level.

The results of our models support the contribution of mice to NIP_All_, but there was varying empirical support for the role of chipmunk and shrew relative abundances on NIP_All_ between the two years. With respect to NIP_HIS_, there was reasonably strong evidence present for mouse and chipmunk relative abundances being negatively associated with NIP_HIS_ in both years, and for varying patterns of associations between shrew relative abundance and NIP_HIS_ across the years. And in both years, there was moderate evidence of a negative association between *H’* and NIP_All_, but a positive association between *H’* and NIP_HIS_. Lastly, NIP_All_ and DIN_All_ were generally higher in 2006 than in 2009, with the model-based estimates providing more powerful inference than naïve estimates or inference based on discarding missing data.

The variation in NIP_All_ and DIN_All_ across our sites in both years highlights the spatial and temporal variability of overall Lyme disease risk within Dutchess County, New York. However, when we examined Lyme disease risk in the context of the HIS phenotype (NIP_HIS_), we found that disease risk was generally and consistently high across these sites, even considering estimation uncertainty, irrespective of their NIP_All_ and DIN_All_. Because NIP_HIS_ is conditional on *ospC* positive ticks, this does not translate directly into HIS risk. Nevertheless, the high HIS prevalence underscores how commonly the HIS phenotype can occur within the tick population, even after accounting for site and year variability.

It is not surprising that the relative abundance of the white-footed mouse would be positively associated with NIP_All_ (year 2006 and 2009, respectively), given that the white-footed mouse is a reservoir-competent host that is efficient at transmitting the bacterium to tick vectors [14,16,18,46]. White-footed mice are also abundant in the community, allowing for potentially higher host-tick feeding opportunities, and thus higher *B*. *burgdorferi* infection prevalence among ticks. Previous studies have demonstrated positive effects of chipmunks on Lyme disease risk [47–49], but our detection of a positive association with NIP_All_ in only one of two study years suggests inconsistent effects of chipmunks on risk. With shrews, the models showed moderate evidence for relative abundances having a positive association with NIP_All_ in 2006 but weaker evidence of a negative association with NIP_All_ in 2009. Shrews are known to have relatively high competency, feed high proportions of ticks, and have high population densities that may increase their contact rates with tick vectors [16,17]. We would expect that shrews, like mice, would have a positive effect on NIP_All_ across both years, but the change in the direction of association highlights the need for further exploration of whether (and how) this host species ultimately influences disease risk.

The moderate, negative association we detected between *H’* and NIP_All_ in both years was consistent with prior results from other sites in the northeastern US [23] and Ontario, Canada [19]. As *H’* (host diversity) increases, the frequency with which black-legged ticks feed and transmit the pathogen from competent hosts decreases [22]. But this observation cannot explain the moderate, positive association between *H’* and NIP_HIS_. Because white-footed mice, eastern chipmunks, short-tailed shrews, and masked shrews are competent reservoirs for *B*. *burgdorferi* [16,17], and because both mice and chipmunks (in particular) commonly carry HIS types [34], we had expected to find increased prevalence of HIS strains in depauperate (low *H’*) communities dominated by mice, chipmunks, and shrews. However, some of our recent work highlights the fact that a wide array of host species are competent at supporting HIS types, and that transmission efficiencies of these strains are relatively high [27]. Therefore, we might expect a positive association between *H’* and NIP_HIS_, as seen in this study. On the other hand, the negative relationship between *H’* and NIP_All_ may reflect the fact that these small rodents are clearly more competent reservoirs overall, so reductions in their relative abundances would help reduce the overall infection prevalence in the tick populations. This is in contrast to the findings by States et al. [50] who noted that overall NIP was similar in tick populations co-occurring with the host communities of an island and the adjacent mainland, where island sites had lower host species richness. Ultimately, understanding and reducing transmission risk would require better epidemiological data linking NIP_HIS_ and NIP_All_ to risk or incidence of Lyme disease in local human populations.

The contrasting results between years for shrew relative abundances with NIP_HIS_ also underscore the need for further investigation. Earlier research by Brisson and Dykhuizen [34] showed that HIS/non-HIS proportions in xenodiagnostic ticks that have fed on shrews are higher, compared to the average HIS/non-HIS proportions of the white-footed mouse, eastern chipmunk, and gray squirrel. Vuong et al. [27] detected similar results for shrews compared with rodents and birds. These positive associations between shrew relative abundance and NIP_HIS_ suggests that shrews can be important components of Lyme disease risk [17], but we still do not understand when and where they are important. Challenges in understanding the ecology of the shrews, and in estimating population densities, can make it difficult to assess when they are important hosts, but these results underscore the importance of shrews in influencing Lyme disease risk.

Our novel Bayesian approach offered a comprehensive way of examining human Lyme disease risk by making site-specific inferences associated with each individual tick tested. Using this analytical method, we were able to draw collective conclusions on the role of host diversity and the relative abundances of mice, chipmunks, and shrews on Lyme disease risk for years 2006 and 2009, and for the sites within those years. Our method was especially effective at capturing the noise and variation associated with each tick individual, hence providing more insights into the relevance of each parameter we examined in the study. As in the case for any regression analysis, definitive conclusions on the more general roles of host diversity and the relative abundances of the three common host species on Lyme disease risk are somewhat conditional on the sampling limitations of the study, e.g. a low number of positive PCR or RLB tests could have resulted from a lack of the disease in the wild and/or the inherent imperfection of the PCR or procedure itself (S6 File). Indeed, we found using the improved primers [41] for the 2009 samples provide consistent RLB outcomes, and using these new primers should be applied in the future. Additionally, our results only provide temporal snapshots of the relationship between risk and hosts. In order to obtain a robust comprehension of how host community influences *B*. *burgdorferi* prevalence in ticks, we need longer-term studies of spatial and temporal trends associated with these important ecological predictors. Knowledge of the long-term population dynamics of important host species and the frequency of different strains of *B*. *burgdorferi*, with different phenotypes affecting human disease risk can help improve our overall understanding of Lyme disease risk.

## Supporting Information

S1 File Field Collections. (PDF)

S2 File Host Activity Density Categories. Activity densities assigned to each host species in the community. Densities for the white-footed mouse, eastern chipmunks, short-tailed shrew, and birds were measured directly.
(PDF)

S3 File Example of a Site-Specific Partition of the Full Dataset for the Bayesian Analyses. Data from all 14 tested ticks sampled from site 602 (*i* = 2) in 2006 are presented. Rows are sorted according to the observed presence/absence of infection (*z*_*ij*_), observed success/failure of RLB test (*v*_*ij*_) if *z*_*ij*_ = 1 (otherwise "NA" for "not applicable"), and observed presence/absence of HIS strains (*t*_*ij*_) if *z*_*ij*_ = *v*_*ij*_ = 1 (otherwise missing data). Boldfaced entries are determined automatically by the value(s) in the preceding column(s).
(PDF)

S4 File Integrative Bayesian GLMs. (PDF)

S5 File Covariate Transformation. (PDF)

S6 File Limitations. (PDF)

S7 File Data (workbook of multiple spreadsheets). (XLS)

S8 File Computer Implementation for Analysis of 2006 Data (R dynamic document). (HTML)

S9 File Computer Implementation for Analysis of 2009 Data (R dynamic document). (HTML)
