# Supplementary material for: Influences of Host Community Characteristics on Borrelia burgdorferi Infection Prevalence in Blacklegged Ticks
Source: PLoS One. 2017 Jan 17;12(1):e0167810. doi: 10.1371/journal.pone.0167810 (PMC5241014; doi:10.1371/journal.pone.0167810)
Supplement: S1 File — (PDF) [file pone.0167810.s001.pdf]

S1 Text. Field Collections

Trapping grid dimensions were adjusted to maximize trapping coverage in smaller forest fragments, but maintained the same distance separation (15m between each Sherman trap and 30m for each Tomahawk trap). Larger fragments in year 2009 had a buffer strip of up to 30 meters with 3 to 30 traps, depending on trap type [1]. Animals captured in the buffer traps were not included in the population estimates for program MARK.

Animals used in this study were approved under the Cary Institute of Ecosystem Studies IACUC numbers 06-03 and 09-01 for field sampling. Animals were live trapped using Sherman or Havahart traps during the late spring to early autumn periods. During the warmer periods of the season, Sherman traps were provided a mix of oats and sunflower seeds for the small animals (e.g. mice, chipmunks). In colder nights, these traps were provided with sunflower seeds and cotton gauze for the animals to create a warm bedding material within the trap. Havahart traps had two raw, unpeeled walnuts for squirrels, and if rain was forecasted, wooden boards were placed over the traps to provide covers for the animals. Traps were opened in the late afternoon and checked early the next morning to avoid any potential heat or cold weather related issues for the animals.

Our estimates of avian diversity for 2006 were based on point-count surveys conducted for six sites in 2004 and 24 sites in 2005. We assume that diversity remained relatively consistent over these years. In 2009, 17 of 19 sites were surveyed for avian hosts. Each site was visited thrice each in 2004 and 2005, and twice each in 2009. All visits were in June, during the peak period of nymphal tick activity [2]. In these visual-audio point-counts, male birds are more likely to be detected than female birds, as male birds are more vocal and active during the breeding season. To correct for male biased observations, we doubled the counts of these birds, using the

maximum number of birds heard across the surveys in each year, and calculated densities based on the 100 m radius plots. Most counts were conducted at the center of the grids, but some counts were conducted off center, due to the irregular shapes of the grid. We focused on the American Robin (*Turdus migratorius*), Veery (*Catharus fuscescens*), Wood Thrush (*Hylocichla mustelina*), and Ovenbird (*Seiurus aurocapilla*) in our Shannon-Weiner diversity estimates because these four primary ground dwelling birds can be important hosts for *B. burgdorferi* [3,4]. We did not have permission at the time in 2009 to conduct an avian visual-audio survey at two sites (site 901 and another that was omitted from statistical modeling), hence the host community Shannon-Weiner diversity estimate for these sites did not include avian estimates.

Our supplemental tick drags occurred after the second density drag estimates to capture additional questing nymphal ticks that were insufficient in numbers at the site in order to test a sufficient number of ticks for reliable *ospC* prevalence estimates and HIS typing. These supplemental drags had no fixed distances and hence, no drag distances were recorded. Due to cost limitation, we did not test all ticks we collected, nor did we identify each individual tick we tested as coming from a density drag or from a supplemental drag. The random testing of ticks collected from each site is an important assumption that enters into the statistical models.

Literature Cited – S1 Text

1. Brunner JL, Duerr S, Keesing F, Killilea ME, Vuong H, Ostfeld RS. 2013. An experimental test of competition among mice, chipmunks, and squirrels in deciduous forest fragments. *PLoS One* 8: 1–9.
2. Ostfeld RS. 1997. The ecology of Lyme-disease risk. *Am Sci* 85: 338–346.
3. Vuong HB, Canham CD, Fonseca DM, Brisson D, Morin PJ, Smouse PE, et al. 2014. Occurrence and transmission efficiencies of *Borrelia burgdorferi ospC* types in avian and

mammalian wildlife. *Infect Genet Evol* 27: 594–600. doi:10.1016/j.meegid.2013.12.011

4. Giardina AR, Schmidt KA, Schauber EM, Ostfeld RS. 2000. Modeling the role of songbirds and rodents in the ecology of Lyme disease. *Can J Zool Can Zool* 78: 2184–2197. doi:10.1139/cjz-78-12-2184
