# Supplementary material for: Influences of Host Community Characteristics on Borrelia burgdorferi Infection Prevalence in Blacklegged Ticks
Source: PLoS One. 2017 Jan 17;12(1):e0167810. doi: 10.1371/journal.pone.0167810 (PMC5241014; doi:10.1371/journal.pone.0167810)
Supplement: S2 File — Activity densities assigned to each host species in the community. Densities for the white-footed mouse, eastern chipmunks, short-tailed shrew, and birds were measured directly. (PDF) [file pone.0167810.s002.pdf]

S2 Table. Host Activity Density Categories

Activity densities assigned to each host species in the community. Densities for the white-footed mouse, eastern chipmunks, short-tailed shrew, and birds were measured directly.

		Activity density class (no. per ha)*			
Common name	Species	Rare/ Absent	Present	Very Common	References
Gray squirrel	*Sciurus carolinensis*	3	7	15	LoGiudice et al. 2003, 2008 and refs within
White-tailed deer	*Odocoileus virginianus*	0	0.1	0.21	LoGiudice et al. 2003, 2008
Virginia opossum	*Didelphis virginiana*	0	0.12	0.9	LoGiudice et al. 2003, 2008
Striped skunk	*Mephitis mephitis*	0	0.04	0.2	LoGiudice et al. 2003, 2008
Raccoon	*Procyon lotor*	0	0.2	0.57	LoGiudice et al. 2003, 2008
Gray fox	*Urocyon cinereoargenteus*	0	0.015	0.062	Trapp & Hallberg 1975, Saunders 1988, Judge & Haviernick 2002
Coyote	*Canis latrans*	0	0.0004	0.005	Hilton 1986, Fortin 1995, Parker 1995, Samson & Crete 1997, Stoddart et al. 2001, Arjo 2004
Red Fox	*Vulpes vulpes*	0	0.003	0.007	Ables 1969, Sargeant 1972, Storm et al. 1976, Pils et al. 1978, Tullar & Berchielli 1980, Voigt and MacDonald 1984

* Species were placed into three activity density classes based on live traps and/or camera traps. The highest quartile is designated as "Very Common" and lower quartiles were considered "Present" or "Rare/Absent".

Literature Cited – S2 Table

1. LoGiudice, K., R.S. Ostfeld, K.A. Schmidt and F. Keesing. 2003. The ecology of infectious disease: effects of host diversity and community composition on Lyme disease risk. *PNAS* 100: 567-571.

2. LoGiudice, K., S.T.K. Duerr, M.J. Newhouse, K.A. Schmidt, M.E. Killilea and R.S. Ostfeld. 2008. Impact of host community composition on Lyme disease risk. *Ecology* 89:2841-2849.

3. Trapp, G.R., and Hallberg, D.L. 1975. Ecology of the gray fox (*Urocyon cinereoargenteus*): a review *in* Fox, M.W., ed. *The wild canids: Their systematics, behavioral ecology and evolution*. Behavioral Science Series. New York: Van Nostrand Reinhold Company: 164-178.

4. Saunders, D.A. 1988. *Adirondack Mammals*. State University of New York, College of Environmental Science and Forestry. 216pp.

5. Judge, K.A., Haviernick, M. 2002. Update COSEWIC status report on the grey fox *Urocyon cinereoargenteus* in Canada *in* COSEWIC assessment and update status report on the grey fox *Urocyon cinereoargenteus* interior in Canada. Committee on the Status of Endangered Wildlife in Canada. Ottawa. 1-32pp.

6. Hilton, H. 1986. Coyote assessment - 1985. Pp. 521-562 *in* Planning for Maine's inland fisheries and wildlife. Maine Department of Inland Fisheries and Wildlife, August, Maine *in* Grompper, M.E. 2002. The Ecology of Northeast Coyotes: Current Knowledge and Priorities for Future Research. WCS Working Paper No 17.

7. Fortin, C. 1995. Ecologie comparee du coyote, du lynx du Canada, et du renard roux au Parc National Forillon. M.Sc. Thesis, Universite Laval, Quebec *in* Grompper, M.E. 2002. The

Ecology of Northeast Coyotes: Current Knowledge and Priorities for Future Research. WCS Working Paper No 17.

8. Parker, G.R. 1995. Eastern coyote. Nimbus Publishing, Halifax *in* Grompper, M.E. 2002. The Ecology of Northeast Coyotes: Current Knowledge and Priorities for Future Research. WCS Working Paper No 17.

9. Samson, C. and M. Crete. 1997. Summer food habits and population density of coyotes, *Canis latrans*, in boreal forests of southeastern Quebec. *Canadian Field Naturalist* 111:227-233.

10. Stoddart, L.C., R.G. Giffiths, and F.F. Knowlton. 2001. Coyote responses to changing jackrabbit abundance affect sheep predation. *Journal of Range Management* 54:15-20.

11. Arjo, W.M. and D.H. Peltsher. 2004. Coyote and wolf habitat use in Northwestern Montana. *Northwest Science* 78: 24-32.

12. Ables, E.D. 1969. Home-range studies of red foxes (*Vulpes vulpes*). *Journal of Mammalogy* 50:108-120.

13. Sargeant, A.B. 1972. Red fox spatial characteristics in relation to waterfowl predation. *Journal of Wildlife Management* 36:225-236.

14. Storm, G.L., R.D. Andrews, R.L. Phillips, R.A. Bishop, D.B. Siniff, and J.R. Tester. 1976. Morphology, reproduction, dispersal, and mortality of midwestern red fox populations. *Wildlife Monographs* 49:3-82.

15. Pils, C.M., M.A. Martin, and R.L. Hine. 1978. Population dynamics, predator-prey relationships and management of the red fox in Wisconsin. Technical bulletin (Wisconsin Department of Natural Resources) 105: 56pgs.

16. Tullar Jr., B.F. and L.T. Berchielli, Jr. 1980. Movement of the red fox in central New York. *N.Y. Fish and Game Journal* 27:179-204.

17. Voigt, D.R. and D.W. MacDonald. 1984. Variation in the spatial and social behaviour of the red fox, *Vulpes vulpes*. *Acta Zoologica Fennica* 171:261-265.
