# Supplementary material for: Influences of Host Community Characteristics on Borrelia burgdorferi Infection Prevalence in Blacklegged Ticks
Source: PLoS One. 2017 Jan 17;12(1):e0167810. doi: 10.1371/journal.pone.0167810 (PMC5241014; doi:10.1371/journal.pone.0167810)
Supplement: S3 File — Data from all 14 tested ticks sampled from site 602 (i = 2) in 2006 are presented. Rows are sorted according to the observed presence/absence of infection (zij), observed success/failure of RLB test (vij) if zij = 1 (otherwise "NA" for "not applicable"), and observed presence/absence of HIS strains (tij) if zij = vij = 1 (otherwise missing data). Boldfaced entries are determined automatically by the value(s) in the preceding column(s). (PDF) [file pone.0167810.s003.pdf]

# S3 Table. Example of a Site-Specific Partition of the Full Dataset for the Bayesian Analyses

Data from all 14 tested ticks sampled from site 602 ($i$=2) in 2006 are presented below.

Rows are sorted according to the observed presence/absence of infection ($z_{ij}$), observed success/failure of RLB test ($v_{ij}$) if $z_{ij}$ = 1 (otherwise "NA" for "not applicable"), and observed presence/absence of HIS strains ($t_{ij}$) if $z_{ij} = v_{ij}$ = 1 (otherwise missing data). Boldfaced entries are determined automatically by the value(s) in the preceding column(s).

| $i$ (Site Label) | Tick Label $j$ (sorted) | $z_{ij}$ | $v_{ij}$ | $t_{ij}$ |
|---|---|---|---|---|
| 2 (602) | 1 | 0 | **NA** | **0** |
| | 2 | 0 | **NA** | **0** |
| | 3 | 0 | **NA** | **0** |
| | 4 | 0 | **NA** | **0** |
| | 5 | 0 | **NA** | **0** |
| | 6 | 0 | **NA** | **0** |
| | 7 | 0 | **NA** | **0** |
| | 8 | 0 | **NA** | **0** |
| | 9 | 1 | 0 | **missing** |
| | 10 | 1 | 0 | **missing** |
| | 11 | 1 | 0 | **missing** |
| | 12 | 1 | 0 | **missing** |
| | 13 | 1 | 1 | 0 |
| | 14 | 1 | 1 | 1 |
