# Supplementary material for: Influences of Host Community Characteristics on Borrelia burgdorferi Infection Prevalence in Blacklegged Ticks
Source: PLoS One. 2017 Jan 17;12(1):e0167810. doi: 10.1371/journal.pone.0167810 (PMC5241014; doi:10.1371/journal.pone.0167810)
Supplement: S4 File — (PDF) [file pone.0167810.s004.pdf]

S4 Text. Integrative Bayesian GLMs

Classical statistical modeling requires the specification of the likelihood function (joint probability distribution of the response data as random variables, given the model parameters). The likelihood is conventionally denoted by $L(\boldsymbol{\theta}) = f(\boldsymbol{y}|\boldsymbol{\theta})$, where $\boldsymbol{\theta}$ and $\boldsymbol{y}$ are the vectors of model parameters and response data, respectively. (In this SI, $\boldsymbol{y}$ comprehensively denotes all non-covariate information rather than merely the observed number of infected ticks as given in Figs 1–2 in the main text.) Bayesian modeling additionally requires the specification of the joint prior distribution (joint probability distribution of model parameters), $\pi(\boldsymbol{\theta})$, to reflect any *a priori* understanding of the behavior of parameters in the absence of the current dataset. Applying Bayes' rule to f and π yields the joint posterior distribution, $p(\boldsymbol{\theta}|\boldsymbol{y})$, which is the basis of Bayesian inference. For a particular parameter θ_r, Bayesian inference refers to the marginal posterior distribution $p(\theta_r|\boldsymbol{y})$. For complex models, closed-form expressions of $p(\boldsymbol{\theta}|\boldsymbol{y})$ often do not exist or are too cumbersome to derive analytically. For this reason, we used Markov chain Monte Carlo (MCMC), a computationally intensive method, to sample from $p(\boldsymbol{\theta}|\boldsymbol{y})$. (See [1] for more detail.) To this end, we implemented all Bayesian models with the MCMC software JAGS version 3.4.0 [2] and above, interfaced through the `runjags` package on R version 3.2.2 and above. The program code for our full Bayesian analyses appears in S8 File (for 2006 data) and S9 File (for 2009 data).

For our Lyme disease studies, all RLB tests in 2009 successfully produced a 1 or 0 for t_{ij} (presence/absence of HIS), but some in 2006 failed to produce results. As such, the GLM constructed for 2009 is a degenerate case of the 2006 model hierarchy. In what follows, we focus the discussion on the hierarchical framework of the more complex case, which accounted for the

unobservability of t_{ij} (due to RLB failure in 2006) by regarding the unobserved t_{ij}s as model parameters.

Referring to Fig 3, our binary data vector was $\boldsymbol{y} = [\boldsymbol{z}, \boldsymbol{v}, \boldsymbol{t}^{\text{observed}}]$, and we had the following probabilities:

$\mathrm{P}(z_{ij}=1)$ $= p_i^B$
= Prob(tick *j* from site *i* testing Bb+),
$\mathrm{P}(v_{ij}=1|z_{ij}=1)$ $= p_i^S$
=Prob(RLB success | tick *j* from site *i* testing Bb +),
$\mathrm{P}(t_{ij}=1|v_{ij}=1)$ $= p_i^{SH}$
= Prob(tick testing HIS+ | {tick *j* from site *i* testing Bb+}, {RLB success}),
$\mathrm{P}(t_{ij}=1|v_{ij}=0)$ $= p_i^{FH}$
= Prob(tick would have tested HIS+ had RLB been successful | {tick *j* from site *i* testing Bb+}, {RLB failure})
$\mathrm{P}(t_{ij}=1|z_{ij}=1)$ $= p_i^c$
= Prob(tick testing or would have tested HIS+ | tick *j* from site *i* testing Bb+)
$= p_i^S p_i^{SH} + (1-p_i^S) p_i^{FH}$,
$\mathrm{P}(t_{ij}=1|z_{ij}=0)$ $= 0$.

Little collinearity existed among the four covariates after removing sites with potentially influential covariate values (i.e., sites for which at least one covariate value was visibly isolated from the rest of the data). Thus, for a preliminary model on the full dataset (including potentially influential sites), we considered all 4 covariates (centered – see S5 Text) in each logistic linear predictor:

$$\mathrm{logit}(p_i^B) = \alpha_0 + \alpha_1 x_{1i} + \cdots + \alpha_4 x_{4i} + \eta_i\,,$$
$$\mathrm{logit}(p_i^c) = \gamma_0 + \gamma_1 x_{1i} + \cdots + \gamma_4 x_{4i} + \xi_i$$

where η_i and ξ_i were normally distributed noise terms with variances τ^2 and ω^2, respectively. Altogether, our parameter vector was $\boldsymbol{\theta} = [\boldsymbol{p}^B, \boldsymbol{p}^c, \boldsymbol{p}^S, \boldsymbol{p}^{SH}, \boldsymbol{p}^{FH}, \boldsymbol{\alpha}, \boldsymbol{\gamma}, \tau^2, \omega^2, \boldsymbol{t}^{\text{missing}}]$.

To complete the specification of our Bayesian GLM, we assumed the following flat or nearly flat priors (subject to necessary constraints) for all parameters that appear without parent nodes in Fig 4:

$$\alpha_k, \gamma_k \sim \text{Normal}(0, 1000); \qquad \tau^{-2}, \omega^{-2} \sim \text{Gamma}(1, a);$$

$$p_i^{SH} | p_i^c \sim \text{Uniform}(0, p_i^c); \qquad p_i^{FH} | p_i^c \sim \text{Uniform}(p_i^c, 1)$$

where a was taken to be either 0.1 (prior less vague) or 0.01 (prior more vague), depending on the computational efficiency (see S8 File, S9 File). Aside from the bounds in the uniform priors that were necessary to preserve the mathematical relationship $p_i^S = \left(1 - \frac{p_i^c}{p_i^{FH}}\right)\left(1 - \frac{p_i^{SH}}{p_i^{FH}}\right)^{-1} = (p_i^{FH} - p_i^c)/(p_i^{FH} - p_i^{SH})$, these vague priors reflect our *a priori* ignorance of these parameters' behavior.

Bayesian inference results

Bayesian inference based on the preliminary model (including all 4 covariates) suggested little evidence that a site's chipmunk relative abundance was relevant to p^B in 2009. Thus, for reduced model fits, we set α_3= 0 for 2009. Selected posterior summaries for the reduced models appear in Table 1. The same summaries appear graphically in Fig S4.1.

We diagnosed the goodness-of-fit of each final model by (a) examining "residual plots" that displayed violin plots of posterior distributions for η_i and ξ_i against each covariate and i, and (b) conducting posterior predictive cross-validation based on $\boldsymbol{z}$.

For (a), we found that even with the large amount of 0 data for z_{ij} and the presence of influential data, our residual plots showed no reason for concern of poor fit. Fig S4.2 shows examples of our residual plots.

For (b), we made posterior predictions only for $\boldsymbol{z}$ as the primary response data, while $\boldsymbol{v}$ and $\boldsymbol{t}$ were conditional on $\boldsymbol{z}$ and were therefore secondary response data. As described in the main text, for each site *i* we simulated predicted values of $\sum_j z_{ij}$ (denoted *y* in the main text)

from the posterior distribution. Denote posterior predictive results by a tilde ("~"). Then, our ith posterior predictive distribution of $\sum_j \tilde{z}_{ij}$ constituted several thousands of simulated predicted values of $\sum_j z_{ij}$. This led to the ith posterior predictive distribution of naïve $\mathrm{DIN_{All}}$ $(= m_i a_i^{-1} n_i^{-1} \sum_j \tilde{z}_{ij})$. Thus, each 95% "predictive" interval in Figs 1C, 2C (in black) was obtained by simply scaling by a factor of $m_i a_i^{-1} n_i^{-1}$ the 2.5th and 97.5th percentiles among the several thousands of $\sum_j \tilde{z}_{ij}$. Except for site 615 in 2006 and sites 914 and 918 in 2009, the remaining (29+16)/(30+18)=94% of the studied sites yielded 95% "predictive" intervals that contained the observed naïve estimates. In this regard, our models were highly consistent with the raw data, and hence, had high goodness-of-fit from the perspective of posterior predictions of naïve $\mathrm{DIN_{All}}$.

Note that the posterior medians presented in Figs 1C, 2C are not the 50th percentiles of $\sum_j \tilde{z}_{ij}$ but rather based on the posterior inference for the model parameter p^B (as in Figs 1A, 2A). Each posterior median in Figs 1C, 2C was obtained by scaling the posterior median of p^B by a factor of $m_i a_i^{-1}$, which amounted to a quasi-modeled estimate for $\mathrm{DIN_{All}}$ in the wild. Properly modeled inference for $\mathrm{DIN_{All}}$ would require the modeling of the true DON in the wild. As m_i was not replicated in the field experiment, we could not make mdoel-based inference for DON.

Aside from posterior predictive checks based on $\mathrm{DIN_{All}}$, we conducted additional checks based on the ranking of sites according to their naïve $\mathrm{NIP_{All}}$ estimates, specifically the sample median among the 30 sites in 2006 and that among the 18 sites in 2009 (Fig S4.3). Based on the raw data, the 2006 median was 0.364 (shared by sites 601 and 613), while the 2009 median was 0.199 (shared by sites 901 and 908). For each year, we simulated several thousands of sets of naïve $\mathrm{NIP_{All}}$ $(= n_i^{-1} \sum_j \tilde{z}_{ij}$ for i = 1, 2, …, 30 in 2006 or i = 1, 2, …, 18 in 2009), each set

constituting a single ordering of the 30 sites in 2006 or of the 18 sites in 2009. From each set of ordering we obtained the median. The several thousands of posterior predictive medians thus obtained formed the respective distributions shown in Fig S4.3. Comparing the posterior predictive distributions to the raw values based on the observed data (shown in red), again we see a high level of consistency between our model and the raw data. In fact, each year's 90% predictive interval contained the raw value from that year (0.273 < 0.364 < 0.380 for 2006, and 0.128 < 0.199 < 0.228 for 2009). Therefore, the two-sided posterior predictive p-value (probability that the posterior predictive naïve NIP_{All} value would be at least as extreme as the observed value) was > 0.1 for each year. (Small two-sided p-values would suggest poor fit.)

The high goodness-of-fit of our models on the whole based on (a) and (b) does not preclude isolated anomalies in the fit. In particular, an anomaly for the 2009 analysis was that the posterior median for p_i^c was always > 0.6, and was between 0.9 and 1.0 for 9 of the 18 sites. Due to this lack of spread, it is possible that the high posterior probabilities presented in Table 1 for NIP_{HIS}-related regression coefficients (γ_ks) in 2009 were a pure artifact of statistical significance on the logit scale rather than practical significance in the ecological sense.

For 2006, we additionally fitted a model that omitted $\boldsymbol{v}$ from the data vector $\boldsymbol{y}$. This naive model and the fully integrated model both yielded comparable inferences for infection-related parameters, namely, $\boldsymbol{p}^B, \boldsymbol{\alpha}$, and τ^2. However, the naive model yielded noticeably bigger posterior standard deviations (i.e., weaker inference) for NIP_{HIS}-related parameters, namely, $\boldsymbol{p}^c, \boldsymbol{\gamma}$, and ω^2. Thus, modeling $\boldsymbol{v}$ alongside $\boldsymbol{z}$ and $\boldsymbol{t}$ helped to improve our overall inference.

Finally, we investigated the potential effect of the confounding false and true positives/negatives on our inference (see S6 Text for more on confounding). Throughout the paper, we have referred to $p_i^B, p_i^{SH}, p_i^{FH}$, and p_i^c as disease detection probabilities. It was out of

the scope of our studies to rigorously assess the actual discrepancy between p_i^B and $\mathrm{NIP}_{\mathrm{All}}$ or between p_i^c and conditional $\mathrm{NIP}_{\mathrm{HIS}}$. Still, we compared our inference to that obtained from a model that considered the marginal likelihood for $\boldsymbol{t}$, as follows.

Hypothetically, suppose RLB tests had been administered for all sampled ticks, even if $z_{ij} = 0$. That is, pretend that each incidence of t_{ij} (0, 1, or missing) had been observed from an RLB test, irrespective of the value of z_{ij}. Then, $p_i^m = \mathrm{Prob}(t_{ij} = 1) = p_i^B p_i^c$ would be the marginal probability for any tick to test HIS+. Thus, for each year, we additionally fitted an integrated model based on $\mathrm{Prob}(t_{ij} = 1) = p_i^B p_i^c$, and compared the resulting inference to the model based on reality, i.e., based on $\mathrm{Prob}(t_{ij} = 1 \mid z_{ij} = 1) = p_i^c$ and $\mathrm{Prob}(t_{ij} = 1 \mid z_{ij} = 0) = 0$, as described throughout this paper. For both years, the hypothetical and realistic models yielded comparable inferences for infection-related parameters. This could be expected, because in each year, $\boldsymbol{z}$ was fully observed and modeled identically between models. However, inferences differed noticeably in various respects of $\mathrm{NIP}_{\mathrm{HIS}}$-related parameters. For 2006, the posterior medians for γ_0 and some p_i^cs were noticeably different between models, and so were the posterior standard deviations (SDs) for ω^2, some γ_ks and some p_i^cs (on the logit scale). For 2009, the hypothetical model slightly reduced the posterior SD for infection-related parameters, but at the expense of hugely increased posterior SDs for $\mathrm{NIP}_{\mathrm{HIS}}$-related parameters. In both years, the hypothetical model was much worse in predictive performance according to the deviance information criterion (Table S4.1).

Table S4.1. Deviance information criteria (DIC), computed as the mean deviance plus half the deviance variance [3]. Smaller DIC values suggest better predictive performance. Note that DIC can be compared only between models that involve the same $\boldsymbol{y}$, and thus cannot be compared between years.

Model (all covariates)	2006 DIC	2009 DIC
hypothetical	> 1600	> 950
realistic	< 1500	< 650

Fig S4.1. Graphical representation of Table 1 in the main text. Panels A and C: Estimates (posterior medians) of regression slope parameters. Panels B and D: Posterior probabilities of positive or negative association between covariates and NIP_{All} (Panel B) or NIP_{HIS} (Panel D). Blue plot symbols correspond to positive slope estimates, whereas red symbols correspond to negative slope estimates. Each plot symbol denotes the corresponding covariate under consideration, namely, 'H' for Shannon-Weiner diversity, 'M' for Mouse relative abundance, 'C' for Chipmunk relative abundance, and 'S' for Shrew relative abundance. For example, the largest value for 2006 in Panel B is the symbol 'M' in blue at a value of 0.96, indicating a posterior probability of 0.96 that mouse relative abundance is positively associated with NIP_{All}.

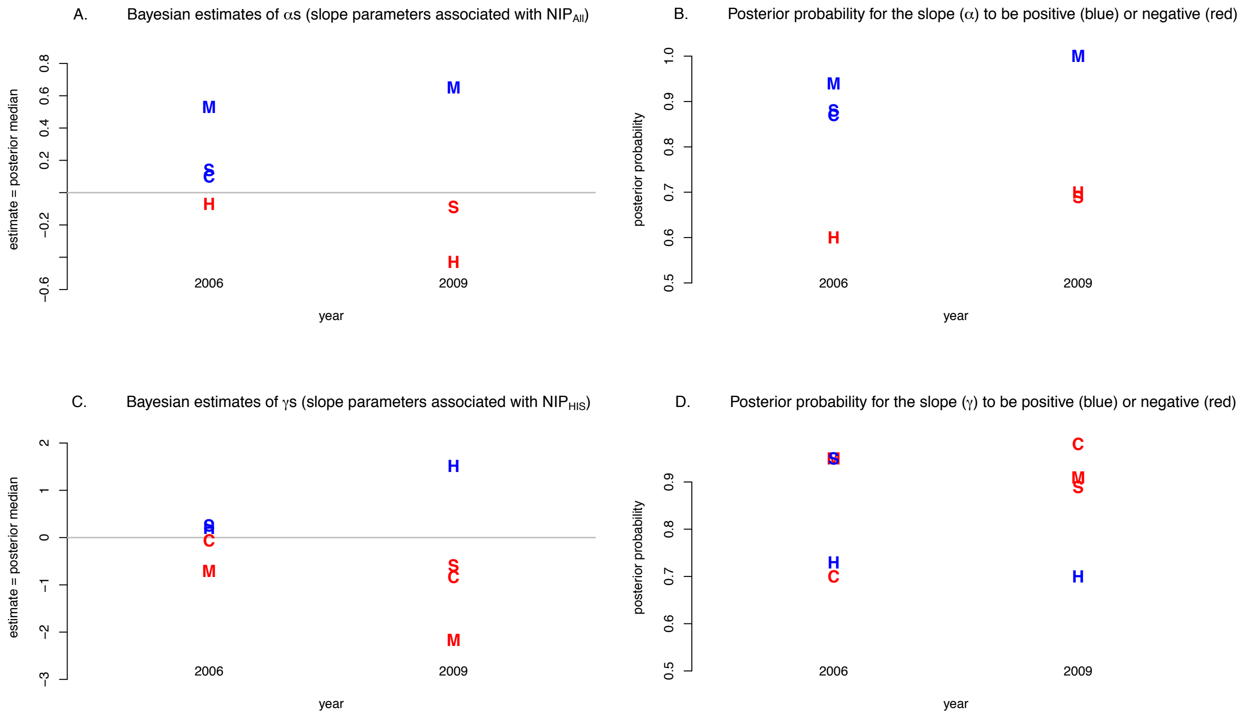

Fig S4.2. Examples of residual plots made up of violin plots of posterior distributions of η_is and ξ_is; the left half of any violin plot is the posterior distribution of the corresponding η_i or ξ_i, and the right half is its mirror image.

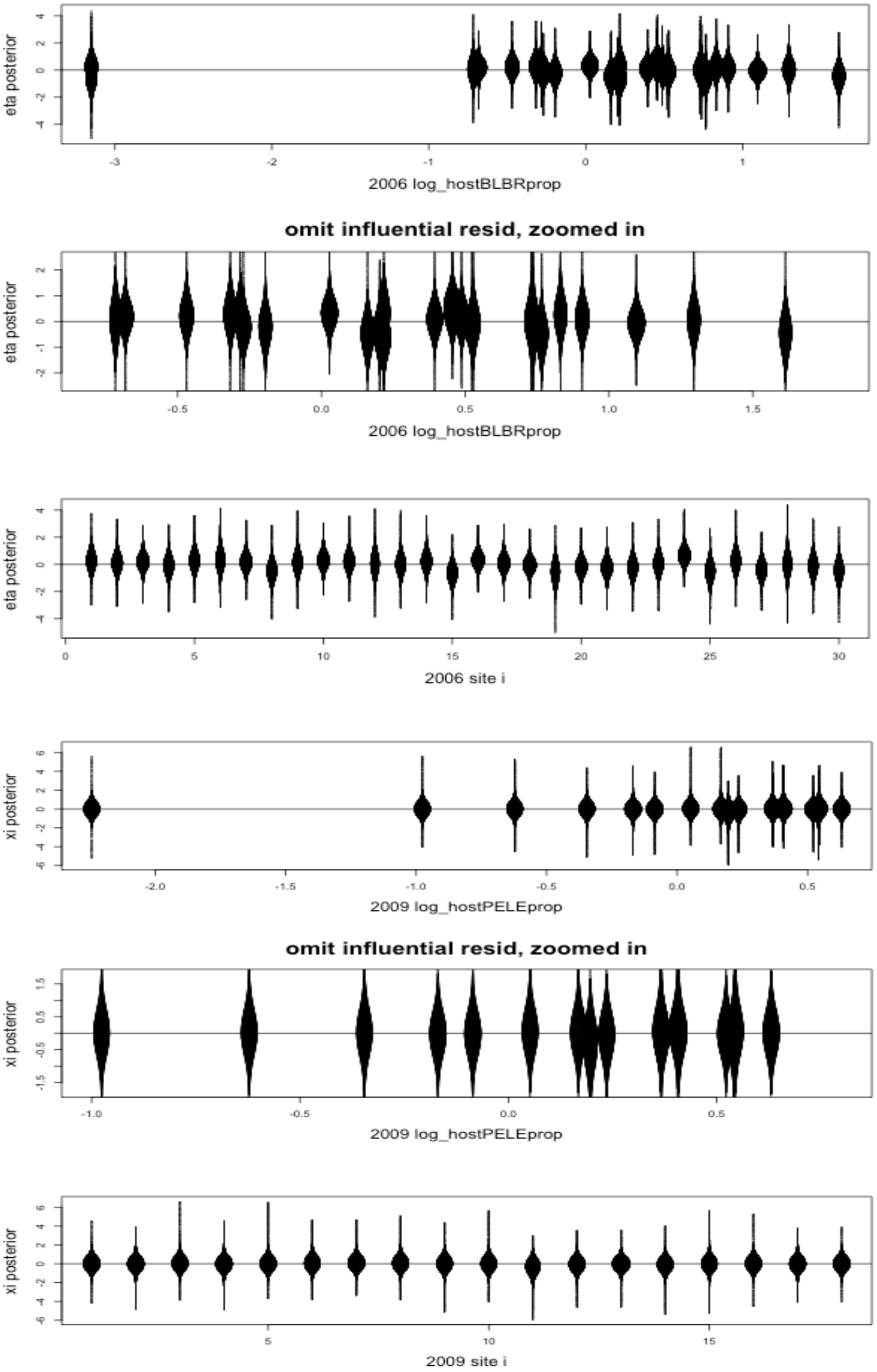

Fig S4.3. Posterior predictive distributions of the median value for $n_i^{-1} \sum_j z_{ij}$, where "median" is defined respectively as the average between the 15th and 16th largest among the 30 sites in 2006 (top panel), and the average between the 9th and 10th largest among the 18 sites in 2009 (bottom panel). In the case of multiple sites yielding identical predicted values of $\sum_j \tilde{z}_{ij}$, the sites were ordered according to S7 File. See S8 File and S9 File for full details and additional posterior predictive summaries.

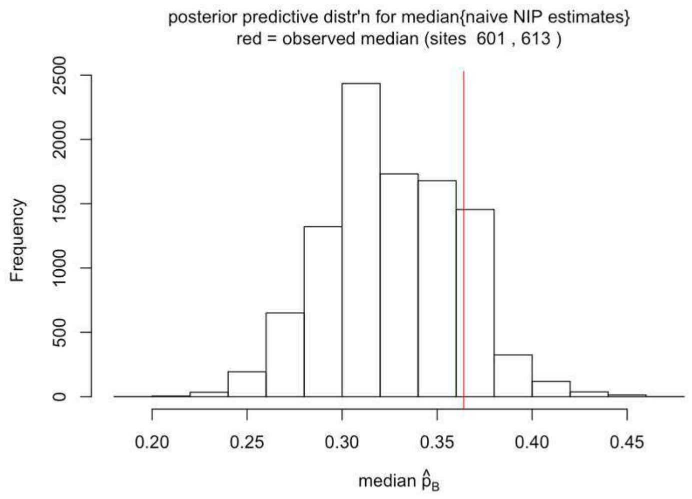

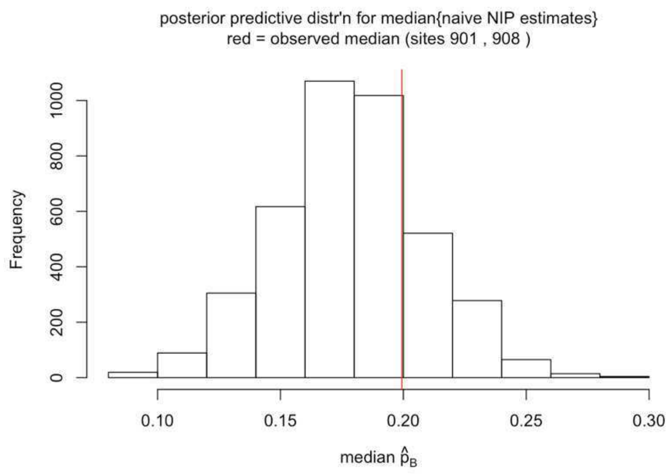

Literature Cited – S4 Text

1. Gelman A, Hill J. 2006. *Data Analysis Using Regression and Multilevel/Hierarchical Models*. Cambridge University Press.
2. Plummer M. 2013. JAGS v 3.4.0. http://mcmc-jags.sourceforge.net
3. Gelman A, Carlin JB, Stern HS, Dunson DB, Vehtari A, Ruben DS. 2013. *Bayesian Data Analysis*. 3rd ed. Chapman and Hall/CRC.
