# Supplementary material for: Influences of Host Community Characteristics on Borrelia burgdorferi Infection Prevalence in Blacklegged Ticks
Source: PLoS One. 2017 Jan 17;12(1):e0167810. doi: 10.1371/journal.pone.0167810 (PMC5241014; doi:10.1371/journal.pone.0167810)
Supplement: S5 File — (PDF) [file pone.0167810.s005.pdf]

S5 Text. Covariate Transformation

Let $x_{1i}, \ldots, x_{4i}$ denote, respectively, the ith site's covariate values for host diversity H', and relative abundances of mouse species, chipmunk species, and shrew species. Note that x_{2i}, x_{3i}, x_{4i} were shifted logarithms of percentages. Log-transformation was employed to improve the spread of these relative abundances. To preserve interpretability of those data, which often scored 0%, we used a shift of +0.5% that imitated the conventional continuity correction for count data.

For our Bayesian analyses, all x_{ki}s values had been additionally centered (after log-transformation, if applicable) so that the mean covariate value averaged over all sites was 0 (i.e., $\overline{x}_k$ = 0). Covariate centering is common for Bayesian GLMs [1-3] to improve the computational performance of simulating from the posterior distribution while fully preserving linearity of the GLM linear predictor.

Literature Cited – S5 Text

1. Chiu GS, Westveld AH. 2011. A unifying approach for food webs, phylogeny, social networks, and statistics. *Proc Natl Acad Sci U S A.*; 108: 15881–6. DOI:10.1073/pnas.1015359108

2. Chiu GS, Westveld AH. 2014. A statistical social network model for consumption data in trophic food webs. *Stat Methodol.*; 17: 139–160. DOI:10.1016/j.stamet.2013.09.001

3. Chiu GS, Wu MA, Lu L. 2013. Model-based assessment of estuary ecosystem health using the latent health factor index, with application to the Richibucto Estuary. *PLoS One*; 9: e65697. DOI: 10.1371/journal.pone.0065697
