# Supplementary material for: Influences of Host Community Characteristics on Borrelia burgdorferi Infection Prevalence in Blacklegged Ticks
Source: PLoS One. 2017 Jan 17;12(1):e0167810. doi: 10.1371/journal.pone.0167810 (PMC5241014; doi:10.1371/journal.pone.0167810)
Supplement: S6 File — (PDF) [file pone.0167810.s006.pdf]

S6 Text. Limitations

Note that all our models regarded the number of test ticks as part of the experimental design. Thus, the uncertainty in the number of test ticks from each site was not modeled in our statistical analyses, because of the absence of data on transect distances during field sampling and the arbitrariness of the actual number of ticks that were subjected to the PCR procedure. Even if our field and laboratory procedures had allowed us to construct models to capture this uncertainty, the resulting models would have been much more complex, thus possibly requiring an impractically large number of test ticks for extra statistical power in order to overcome model complexity.

Another limitation of our models is the confounding between true and false positives, and between true and false negatives. That is, our data did not allow us to identify whether a positive or negative lab result correctly reflected the true state of the tick. As we did not have external sources of information regarding false positive and false negative rates of either the PCR or RLB procedures, the distinction could not be incorporated into our models. The limitation, then, is in our conclusions regarding the relationship between covariates and response. For example, in the Bayesian analyses, NIP_{All} in fact refers to the *probability of detecting B. burgdorferi*, and conditional NIP_{HIS}, to the *probability of detecting HIS on a tick that has been identified as infected*. None of our conclusions directly translates to the actual underlying prevalence of either *B. burgdorferi* or HIS, the latter of which was entirely ignored among ticks that were tested negative for *B. burgdorferi*. This is because, following standard practice, we assigned t_{ij} the value 0 (detected absence of HIS strains) whenever z_{ij}= 0 (detected absence of infection), irrespective of the underlying (but unknowable) values of z_{ij}^{true} or t_{ij}^{true}, both of which could

have been 1. It is understood that neither of the PCR and RLB lab procedures was perfectly accurate; accuracy also varies from year to year due to changing industry standards in primer technology. Thus, z_{ij}=0 could have been observed on an infected tick (z_{ij}^{true}=1) (and *vice versa*), and similarly for t_{ij} and t_{ij}^{true} referring to detection/existence of HIS strains. We have less concern over this confounding regarding the conclusions about NIP_{All}, because it is reasonable to assume that the inherent PCR inadequacies in any given year had a somewhat constant effect (if any) across all sites, and such constancy should not bias conclusions about statistical evidence of covariates' influence on the response. However, the same argument may not apply to NIP_{HIS}. This is because the RLB's inadequacies can only have an effect on the data analysis through PCR procedures that lead to $z_{ij} = 1$. For instance, in the extreme case that the PCR yielded $z_{ij} = 1$ for all ticks from the *i*-th site, then the RLB's inadequacies would have contributed to the data analysis through the value of t_{ij} observed on each *j*-th tick from this site. In contrast, if all ticks from the *k*-th site had $z_{ij} = 0$, then the RLB's inadequacies would play no part whatsoever in the data analysis because the PCR's results would have fully determined the value t_{kj}=0 for each *j* without the RLB being administered.
