# Supplementary material for: Influences of Host Community Characteristics on Borrelia burgdorferi Infection Prevalence in Blacklegged Ticks
Source: PLoS One. 2017 Jan 17;12(1):e0167810. doi: 10.1371/journal.pone.0167810 (PMC5241014; doi:10.1371/journal.pone.0167810)
Supplement: S8 File — (HTML) [file pone.0167810.s008.html]

 

 

 

 
 
 


 

 

 Supporting Information S8 for Vuong, Chiu, Smouse, Fonseca, Brisson, Morin, and Ostfeld (2016). PLOS ONE. 

 
 
 
 
 
 

 
 
 
 
 


 


 

 

 


 

 
 
 

 


 


 Supporting Information S8 for Vuong, Chiu, Smouse, Fonseca, Brisson, Morin, and Ostfeld (2016).  PLOS ONE . 

 


 
 

  library(coda)
library(runjags)

head(tick2)  
  ##             d  m  n lyme hostHindexAll emptyVariable log_hostPELEprop
##      601  360  4 22    8         1.819                -1.1301030
##      602  360 13 14    6         1.890                -0.7550226
##      603  240 44 44   15         2.568                -1.1488535
##      604  360 17 13    4         1.582                -0.7918632
##      605  360 40 16    7         2.017                -0.4019712
##      606  330  0 22   11         0.555                -0.1996712
##           log_hostTASTprop log_hostBLBRprop
##      601         -5.298317        -1.316768
##      602         -1.987774        -1.241329
##      603         -1.067114        -2.830218
##      604         -2.180367        -1.619488
##      605         -5.298317        -2.465104
##      606         -5.298317        -1.931022  
 
 Preliminary JAGS model 
 
 Realistic model reflecting RLB success/failure 
  \[  
\begin{aligned}  
\text{[2.2a]} &&  
\left. z_{ij} \right| p_i^B \sim \text{(ind) Bernoulli}(p_i^B) \\  
\text{[2.3]} &&  
\left. \text{logit } p_i^B \right| \alpha_0, \boldsymbol{\alpha}, \tau^2, \boldsymbol{x}_i \sim \text{(ind) } N(\alpha_0 + \boldsymbol{x}_i ' \boldsymbol{\alpha}, \tau^2) \\  
\text{[2.6]} &&  
\left. v_{ij} \right| \{z_{ij}=1\}, p_i^S \sim \text{(ind) Bernoulli}(p_i^S) \\  
\text{[2.4c]} &&  
\left. t_{ij} \right| \{z_{ij}=1\}, \{v_{ij}=1\}, p_i^{SH} \sim \text{(ind) Bernoulli}(p_i^{SH}) \\  
\text{[2.4c1]} &&  
\left. t_{ij} \right| \{z_{ij}=1\}, \{v_{ij}=0\}, p_i^{FH} \sim \text{(ind) Bernoulli}(p_i^{FH}) \\  
\text{[2.7]} &&  
p_i^S = \frac{1-p_i^c/p_i^{FH}}{1-p_i^{SH}/p_i^{FH}} \\
&& p_i^{SH} < p_i^c < p_i^{FH} \\
\text{[2.5]} &&  
\text{logit } p_i^c \left| \gamma_0, \boldsymbol{\gamma}, \omega^2, \boldsymbol{x}_i \right. \sim \text{(ind) } N(\gamma_0 + \boldsymbol{x}_i ' \boldsymbol{\gamma}, \omega^2) \\  
\end{aligned}  
\]  
  print(dat.c) # covariates have been centered  
  ## $S
## [1] 30
## 
## $x
##                hostHindexAll emptyVariable log_hostPELEprop log_hostTASTprop
##      601         -0.15313333                   -0.386414919      -2.77860025
##      602         -0.08213333                   -0.011334547       0.53194277
##      603          0.59586667                   -0.405165468       1.45260350
##      604         -0.39013333                   -0.048175117       0.33934966
##      605          0.04486667                    0.341716818      -2.77860025
##      606         -1.41713333                    0.544016842      -2.77860025
##      607          0.73686667                   -0.258705394       1.39270536
##      608          0.01186667                    0.062469427      -2.77860025
##      609         -0.13613333                   -0.059274010       2.01055678
##      610          0.67486667                    0.163869542       0.47949629
##      611         -0.21713333                    0.459997986      -0.86167763
##      612         -0.08413333                   -0.017737984       1.14929611
##      613         -0.16713333                   -0.272423030       1.54155098
##      614         -0.20913333                    0.387013093       1.67574705
##      615          0.06086667                    0.195506627      -0.04423274
##      616          0.36086667                    0.268872851       0.78244584
##      617          0.66686667                    0.247751026      -0.21365089
##      618          0.30586667                    0.311365475       0.94949992
##      619          0.33586667                    0.188562154       0.03080245
##      620          0.56186667                    0.127501898       0.24669083
##      621         -0.35113333                   -0.518620344       1.19921050
##      622          0.69686667                   -0.275189284       0.46399211
##      623         -0.31313333                   -0.323425585       1.12941474
##      624          0.38586667                   -0.075022367       0.32149204
##      625          0.21386667                    0.278472924      -2.77860025
##      626         -0.18013333                    0.458669082      -2.77860025
##      627         -0.37313333                    0.249391715       1.56520518
##      628         -0.91313333                   -2.036932857       1.85806861
##      629         -0.36913333                    0.408215301      -2.77860025
##      630         -0.29713333                   -0.004971854       1.44969229
##                log_hostBLBRprop
##      601             0.83156557
##      602             0.90700528
##      603            -0.68188397
##      604             0.52884562
##      605            -0.31677015
##      606             0.21731233
##      607             0.48760266
##      608             0.16055952
##      609             0.73364003
##      610             0.45551435
##      611            -0.28208460
##      612            -0.71637014
##      613             0.72951632
##      614            -0.46896197
##      615             0.21731233
##      616             0.02807033
##      617             0.39387018
##      618             1.09565051
##      619            -3.14998350
##      620             0.52378232
##      621            -0.27078504
##      622            -0.19507322
##      623             1.29501794
##      624             0.45551435
##      625             0.76603153
##      626            -3.14998350
##      627             0.20342322
##      628            -3.14998350
##      629             0.73774682
##      630             1.61389838
## 
## $n_noLyme
##            601            602            603            604            605 
##             14              8             29              9              9 
##            606            607            608            609            610 
##             11             22             28              4             17 
##            611            612            613            614            615 
##              9              1              7             11             27 
##            616            617            618            619            620 
##             20             21             28             19             22 
##            621            622            623            624            625 
##             26             13              6             18             24 
##            626            627            628            629            630 
##             24             21             26              8             13 
## 
## $z_noLyme
##       [,1] [,2] [,3] [,4] [,5] [,6] [,7] [,8] [,9] [,10] [,11] [,12] [,13]
##  [1,]    0    0    0    0    0    0    0    0    0     0     0     0     0
##  [2,]    0    0    0    0    0    0    0    0 -999  -999  -999  -999  -999
##  [3,]    0    0    0    0    0    0    0    0    0     0     0     0     0
##  [4,]    0    0    0    0    0    0    0    0    0  -999  -999  -999  -999
##  [5,]    0    0    0    0    0    0    0    0    0  -999  -999  -999  -999
##  [6,]    0    0    0    0    0    0    0    0    0     0     0  -999  -999
##  [7,]    0    0    0    0    0    0    0    0    0     0     0     0     0
##  [8,]    0    0    0    0    0    0    0    0    0     0     0     0     0
##  [9,]    0    0    0    0 -999 -999 -999 -999 -999  -999  -999  -999  -999
## [10,]    0    0    0    0    0    0    0    0    0     0     0     0     0
## [11,]    0    0    0    0    0    0    0    0    0  -999  -999  -999  -999
## [12,]    0 -999 -999 -999 -999 -999 -999 -999 -999  -999  -999  -999  -999
## [13,]    0    0    0    0    0    0    0 -999 -999  -999  -999  -999  -999
## [14,]    0    0    0    0    0    0    0    0    0     0     0  -999  -999
## [15,]    0    0    0    0    0    0    0    0    0     0     0     0     0
## [16,]    0    0    0    0    0    0    0    0    0     0     0     0     0
## [17,]    0    0    0    0    0    0    0    0    0     0     0     0     0
## [18,]    0    0    0    0    0    0    0    0    0     0     0     0     0
## [19,]    0    0    0    0    0    0    0    0    0     0     0     0     0
## [20,]    0    0    0    0    0    0    0    0    0     0     0     0     0
## [21,]    0    0    0    0    0    0    0    0    0     0     0     0     0
## [22,]    0    0    0    0    0    0    0    0    0     0     0     0     0
## [23,]    0    0    0    0    0    0 -999 -999 -999  -999  -999  -999  -999
## [24,]    0    0    0    0    0    0    0    0    0     0     0     0     0
## [25,]    0    0    0    0    0    0    0    0    0     0     0     0     0
## [26,]    0    0    0    0    0    0    0    0    0     0     0     0     0
## [27,]    0    0    0    0    0    0    0    0    0     0     0     0     0
## [28,]    0    0    0    0    0    0    0    0    0     0     0     0     0
## [29,]    0    0    0    0    0    0    0    0 -999  -999  -999  -999  -999
## [30,]    0    0    0    0    0    0    0    0    0     0     0     0     0
##       [,14] [,15] [,16] [,17] [,18] [,19] [,20] [,21] [,22] [,23] [,24]
##  [1,]     0  -999  -999  -999  -999  -999  -999  -999  -999  -999  -999
##  [2,]  -999  -999  -999  -999  -999  -999  -999  -999  -999  -999  -999
##  [3,]     0     0     0     0     0     0     0     0     0     0     0
##  [4,]  -999  -999  -999  -999  -999  -999  -999  -999  -999  -999  -999
##  [5,]  -999  -999  -999  -999  -999  -999  -999  -999  -999  -999  -999
##  [6,]  -999  -999  -999  -999  -999  -999  -999  -999  -999  -999  -999
##  [7,]     0     0     0     0     0     0     0     0     0  -999  -999
##  [8,]     0     0     0     0     0     0     0     0     0     0     0
##  [9,]  -999  -999  -999  -999  -999  -999  -999  -999  -999  -999  -999
## [10,]     0     0     0     0  -999  -999  -999  -999  -999  -999  -999
## [11,]  -999  -999  -999  -999  -999  -999  -999  -999  -999  -999  -999
## [12,]  -999  -999  -999  -999  -999  -999  -999  -999  -999  -999  -999
## [13,]  -999  -999  -999  -999  -999  -999  -999  -999  -999  -999  -999
## [14,]  -999  -999  -999  -999  -999  -999  -999  -999  -999  -999  -999
## [15,]     0     0     0     0     0     0     0     0     0     0     0
## [16,]     0     0     0     0     0     0     0  -999  -999  -999  -999
## [17,]     0     0     0     0     0     0     0     0  -999  -999  -999
## [18,]     0     0     0     0     0     0     0     0     0     0     0
## [19,]     0     0     0     0     0     0  -999  -999  -999  -999  -999
## [20,]     0     0     0     0     0     0     0     0     0  -999  -999
## [21,]     0     0     0     0     0     0     0     0     0     0     0
## [22,]  -999  -999  -999  -999  -999  -999  -999  -999  -999  -999  -999
## [23,]  -999  -999  -999  -999  -999  -999  -999  -999  -999  -999  -999
## [24,]     0     0     0     0     0  -999  -999  -999  -999  -999  -999
## [25,]     0     0     0     0     0     0     0     0     0     0     0
## [26,]     0     0     0     0     0     0     0     0     0     0     0
## [27,]     0     0     0     0     0     0     0     0  -999  -999  -999
## [28,]     0     0     0     0     0     0     0     0     0     0     0
## [29,]  -999  -999  -999  -999  -999  -999  -999  -999  -999  -999  -999
## [30,]  -999  -999  -999  -999  -999  -999  -999  -999  -999  -999  -999
##       [,25] [,26] [,27] [,28] [,29]
##  [1,]  -999  -999  -999  -999  -999
##  [2,]  -999  -999  -999  -999  -999
##  [3,]     0     0     0     0     0
##  [4,]  -999  -999  -999  -999  -999
##  [5,]  -999  -999  -999  -999  -999
##  [6,]  -999  -999  -999  -999  -999
##  [7,]  -999  -999  -999  -999  -999
##  [8,]     0     0     0     0  -999
##  [9,]  -999  -999  -999  -999  -999
## [10,]  -999  -999  -999  -999  -999
## [11,]  -999  -999  -999  -999  -999
## [12,]  -999  -999  -999  -999  -999
## [13,]  -999  -999  -999  -999  -999
## [14,]  -999  -999  -999  -999  -999
## [15,]     0     0     0  -999  -999
## [16,]  -999  -999  -999  -999  -999
## [17,]  -999  -999  -999  -999  -999
## [18,]     0     0     0     0  -999
## [19,]  -999  -999  -999  -999  -999
## [20,]  -999  -999  -999  -999  -999
## [21,]     0     0  -999  -999  -999
## [22,]  -999  -999  -999  -999  -999
## [23,]  -999  -999  -999  -999  -999
## [24,]  -999  -999  -999  -999  -999
## [25,]  -999  -999  -999  -999  -999
## [26,]  -999  -999  -999  -999  -999
## [27,]  -999  -999  -999  -999  -999
## [28,]     0     0  -999  -999  -999
## [29,]  -999  -999  -999  -999  -999
## [30,]  -999  -999  -999  -999  -999
## 
## $t_noLyme
##       [,1] [,2] [,3] [,4] [,5] [,6] [,7] [,8] [,9] [,10] [,11] [,12] [,13]
##  [1,]    0    0    0    0    0    0    0    0    0     0     0     0     0
##  [2,]    0    0    0    0    0    0    0    0 -999  -999  -999  -999  -999
##  [3,]    0    0    0    0    0    0    0    0    0     0     0     0     0
##  [4,]    0    0    0    0    0    0    0    0    0  -999  -999  -999  -999
##  [5,]    0    0    0    0    0    0    0    0    0  -999  -999  -999  -999
##  [6,]    0    0    0    0    0    0    0    0    0     0     0  -999  -999
##  [7,]    0    0    0    0    0    0    0    0    0     0     0     0     0
##  [8,]    0    0    0    0    0    0    0    0    0     0     0     0     0
##  [9,]    0    0    0    0 -999 -999 -999 -999 -999  -999  -999  -999  -999
## [10,]    0    0    0    0    0    0    0    0    0     0     0     0     0
## [11,]    0    0    0    0    0    0    0    0    0  -999  -999  -999  -999
## [12,]    0 -999 -999 -999 -999 -999 -999 -999 -999  -999  -999  -999  -999
## [13,]    0    0    0    0    0    0    0 -999 -999  -999  -999  -999  -999
## [14,]    0    0    0    0    0    0    0    0    0     0     0  -999  -999
## [15,]    0    0    0    0    0    0    0    0    0     0     0     0     0
## [16,]    0    0    0    0    0    0    0    0    0     0     0     0     0
## [17,]    0    0    0    0    0    0    0    0    0     0     0     0     0
## [18,]    0    0    0    0    0    0    0    0    0     0     0     0     0
## [19,]    0    0    0    0    0    0    0    0    0     0     0     0     0
## [20,]    0    0    0    0    0    0    0    0    0     0     0     0     0
## [21,]    0    0    0    0    0    0    0    0    0     0     0     0     0
## [22,]    0    0    0    0    0    0    0    0    0     0     0     0     0
## [23,]    0    0    0    0    0    0 -999 -999 -999  -999  -999  -999  -999
## [24,]    0    0    0    0    0    0    0    0    0     0     0     0     0
## [25,]    0    0    0    0    0    0    0    0    0     0     0     0     0
## [26,]    0    0    0    0    0    0    0    0    0     0     0     0     0
## [27,]    0    0    0    0    0    0    0    0    0     0     0     0     0
## [28,]    0    0    0    0    0    0    0    0    0     0     0     0     0
## [29,]    0    0    0    0    0    0    0    0 -999  -999  -999  -999  -999
## [30,]    0    0    0    0    0    0    0    0    0     0     0     0     0
##       [,14] [,15] [,16] [,17] [,18] [,19] [,20] [,21] [,22] [,23] [,24]
##  [1,]     0  -999  -999  -999  -999  -999  -999  -999  -999  -999  -999
##  [2,]  -999  -999  -999  -999  -999  -999  -999  -999  -999  -999  -999
##  [3,]     0     0     0     0     0     0     0     0     0     0     0
##  [4,]  -999  -999  -999  -999  -999  -999  -999  -999  -999  -999  -999
##  [5,]  -999  -999  -999  -999  -999  -999  -999  -999  -999  -999  -999
##  [6,]  -999  -999  -999  -999  -999  -999  -999  -999  -999  -999  -999
##  [7,]     0     0     0     0     0     0     0     0     0  -999  -999
##  [8,]     0     0     0     0     0     0     0     0     0     0     0
##  [9,]  -999  -999  -999  -999  -999  -999  -999  -999  -999  -999  -999
## [10,]     0     0     0     0  -999  -999  -999  -999  -999  -999  -999
## [11,]  -999  -999  -999  -999  -999  -999  -999  -999  -999  -999  -999
## [12,]  -999  -999  -999  -999  -999  -999  -999  -999  -999  -999  -999
## [13,]  -999  -999  -999  -999  -999  -999  -999  -999  -999  -999  -999
## [14,]  -999  -999  -999  -999  -999  -999  -999  -999  -999  -999  -999
## [15,]     0     0     0     0     0     0     0     0     0     0     0
## [16,]     0     0     0     0     0     0     0  -999  -999  -999  -999
## [17,]     0     0     0     0     0     0     0     0  -999  -999  -999
## [18,]     0     0     0     0     0     0     0     0     0     0     0
## [19,]     0     0     0     0     0     0  -999  -999  -999  -999  -999
## [20,]     0     0     0     0     0     0     0     0     0  -999  -999
## [21,]     0     0     0     0     0     0     0     0     0     0     0
## [22,]  -999  -999  -999  -999  -999  -999  -999  -999  -999  -999  -999
## [23,]  -999  -999  -999  -999  -999  -999  -999  -999  -999  -999  -999
## [24,]     0     0     0     0     0  -999  -999  -999  -999  -999  -999
## [25,]     0     0     0     0     0     0     0     0     0     0     0
## [26,]     0     0     0     0     0     0     0     0     0     0     0
## [27,]     0     0     0     0     0     0     0     0  -999  -999  -999
## [28,]     0     0     0     0     0     0     0     0     0     0     0
## [29,]  -999  -999  -999  -999  -999  -999  -999  -999  -999  -999  -999
## [30,]  -999  -999  -999  -999  -999  -999  -999  -999  -999  -999  -999
##       [,25] [,26] [,27] [,28] [,29]
##  [1,]  -999  -999  -999  -999  -999
##  [2,]  -999  -999  -999  -999  -999
##  [3,]     0     0     0     0     0
##  [4,]  -999  -999  -999  -999  -999
##  [5,]  -999  -999  -999  -999  -999
##  [6,]  -999  -999  -999  -999  -999
##  [7,]  -999  -999  -999  -999  -999
##  [8,]     0     0     0     0  -999
##  [9,]  -999  -999  -999  -999  -999
## [10,]  -999  -999  -999  -999  -999
## [11,]  -999  -999  -999  -999  -999
## [12,]  -999  -999  -999  -999  -999
## [13,]  -999  -999  -999  -999  -999
## [14,]  -999  -999  -999  -999  -999
## [15,]     0     0     0  -999  -999
## [16,]  -999  -999  -999  -999  -999
## [17,]  -999  -999  -999  -999  -999
## [18,]     0     0     0     0  -999
## [19,]  -999  -999  -999  -999  -999
## [20,]  -999  -999  -999  -999  -999
## [21,]     0     0  -999  -999  -999
## [22,]  -999  -999  -999  -999  -999
## [23,]  -999  -999  -999  -999  -999
## [24,]  -999  -999  -999  -999  -999
## [25,]  -999  -999  -999  -999  -999
## [26,]  -999  -999  -999  -999  -999
## [27,]  -999  -999  -999  -999  -999
## [28,]     0     0  -999  -999  -999
## [29,]  -999  -999  -999  -999  -999
## [30,]  -999  -999  -999  -999  -999
## 
## $n_RLB
##            601             602           603            604            605 
##              4              2             12              1              3 
##            606            607            608            609            610 
##              7             11              3              3             14 
##            611            612            613            614            615 
##              7              0              4              6              4 
##            616            617            618            619            620 
##             14             10             13              0              7 
##            621            622            623            624            625 
##              2              3              3             13              1 
##            626            627            628            629            630 
##              8              6              2              2              2 
## 
## $z_RLB
##       [,1] [,2] [,3] [,4] [,5] [,6] [,7] [,8] [,9] [,10] [,11] [,12] [,13]
##  [1,]    1    1    1    1 -999 -999 -999 -999 -999  -999  -999  -999  -999
##  [2,]    1    1 -999 -999 -999 -999 -999 -999 -999  -999  -999  -999  -999
##  [3,]    1    1    1    1    1    1    1    1    1     1     1     1  -999
##  [4,]    1 -999 -999 -999 -999 -999 -999 -999 -999  -999  -999  -999  -999
##  [5,]    1    1    1 -999 -999 -999 -999 -999 -999  -999  -999  -999  -999
##  [6,]    1    1    1    1    1    1    1 -999 -999  -999  -999  -999  -999
##  [7,]    1    1    1    1    1    1    1    1    1     1     1  -999  -999
##  [8,]    1    1    1 -999 -999 -999 -999 -999 -999  -999  -999  -999  -999
##  [9,]    1    1    1 -999 -999 -999 -999 -999 -999  -999  -999  -999  -999
## [10,]    1    1    1    1    1    1    1    1    1     1     1     1     1
## [11,]    1    1    1    1    1    1    1 -999 -999  -999  -999  -999  -999
## [12,] -999 -999 -999 -999 -999 -999 -999 -999 -999  -999  -999  -999  -999
## [13,]    1    1    1    1 -999 -999 -999 -999 -999  -999  -999  -999  -999
## [14,]    1    1    1    1    1    1 -999 -999 -999  -999  -999  -999  -999
## [15,]    1    1    1    1 -999 -999 -999 -999 -999  -999  -999  -999  -999
## [16,]    1    1    1    1    1    1    1    1    1     1     1     1     1
## [17,]    1    1    1    1    1    1    1    1    1     1  -999  -999  -999
## [18,]    1    1    1    1    1    1    1    1    1     1     1     1     1
## [19,] -999 -999 -999 -999 -999 -999 -999 -999 -999  -999  -999  -999  -999
## [20,]    1    1    1    1    1    1    1 -999 -999  -999  -999  -999  -999
## [21,]    1    1 -999 -999 -999 -999 -999 -999 -999  -999  -999  -999  -999
## [22,]    1    1    1 -999 -999 -999 -999 -999 -999  -999  -999  -999  -999
## [23,]    1    1    1 -999 -999 -999 -999 -999 -999  -999  -999  -999  -999
## [24,]    1    1    1    1    1    1    1    1    1     1     1     1     1
## [25,]    1 -999 -999 -999 -999 -999 -999 -999 -999  -999  -999  -999  -999
## [26,]    1    1    1    1    1    1    1    1 -999  -999  -999  -999  -999
## [27,]    1    1    1    1    1    1 -999 -999 -999  -999  -999  -999  -999
## [28,]    1    1 -999 -999 -999 -999 -999 -999 -999  -999  -999  -999  -999
## [29,]    1    1 -999 -999 -999 -999 -999 -999 -999  -999  -999  -999  -999
## [30,]    1    1 -999 -999 -999 -999 -999 -999 -999  -999  -999  -999  -999
##       [,14]
##  [1,]  -999
##  [2,]  -999
##  [3,]  -999
##  [4,]  -999
##  [5,]  -999
##  [6,]  -999
##  [7,]  -999
##  [8,]  -999
##  [9,]  -999
## [10,]     1
## [11,]  -999
## [12,]  -999
## [13,]  -999
## [14,]  -999
## [15,]  -999
## [16,]     1
## [17,]  -999
## [18,]  -999
## [19,]  -999
## [20,]  -999
## [21,]  -999
## [22,]  -999
## [23,]  -999
## [24,]  -999
## [25,]  -999
## [26,]  -999
## [27,]  -999
## [28,]  -999
## [29,]  -999
## [30,]  -999
## 
## $v_RLB
##       [,1] [,2] [,3] [,4] [,5] [,6] [,7] [,8] [,9] [,10] [,11] [,12] [,13]
##  [1,]    1    1    1    1 -999 -999 -999 -999 -999  -999  -999  -999  -999
##  [2,]    1    1 -999 -999 -999 -999 -999 -999 -999  -999  -999  -999  -999
##  [3,]    1    1    1    1    1    1    1    1    1     1     1     1  -999
##  [4,]    1 -999 -999 -999 -999 -999 -999 -999 -999  -999  -999  -999  -999
##  [5,]    1    1    1 -999 -999 -999 -999 -999 -999  -999  -999  -999  -999
##  [6,]    1    1    1    1    1    1    1 -999 -999  -999  -999  -999  -999
##  [7,]    1    1    1    1    1    1    1    1    1     1     1  -999  -999
##  [8,]    1    1    1 -999 -999 -999 -999 -999 -999  -999  -999  -999  -999
##  [9,]    1    1    1 -999 -999 -999 -999 -999 -999  -999  -999  -999  -999
## [10,]    1    1    1    1    1    1    1    1    1     1     1     1     1
## [11,]    1    1    1    1    1    1    1 -999 -999  -999  -999  -999  -999
## [12,] -999 -999 -999 -999 -999 -999 -999 -999 -999  -999  -999  -999  -999
## [13,]    1    1    1    1 -999 -999 -999 -999 -999  -999  -999  -999  -999
## [14,]    1    1    1    1    1    1 -999 -999 -999  -999  -999  -999  -999
## [15,]    1    1    1    1 -999 -999 -999 -999 -999  -999  -999  -999  -999
## [16,]    1    1    1    1    1    1    1    1    1     1     1     1     1
## [17,]    1    1    1    1    1    1    1    1    1     1  -999  -999  -999
## [18,]    1    1    1    1    1    1    1    1    1     1     1     1     1
## [19,] -999 -999 -999 -999 -999 -999 -999 -999 -999  -999  -999  -999  -999
## [20,]    1    1    1    1    1    1    1 -999 -999  -999  -999  -999  -999
## [21,]    1    1 -999 -999 -999 -999 -999 -999 -999  -999  -999  -999  -999
## [22,]    1    1    1 -999 -999 -999 -999 -999 -999  -999  -999  -999  -999
## [23,]    1    1    1 -999 -999 -999 -999 -999 -999  -999  -999  -999  -999
## [24,]    1    1    1    1    1    1    1    1    1     1     1     1     1
## [25,]    1 -999 -999 -999 -999 -999 -999 -999 -999  -999  -999  -999  -999
## [26,]    1    1    1    1    1    1    1    1 -999  -999  -999  -999  -999
## [27,]    1    1    1    1    1    1 -999 -999 -999  -999  -999  -999  -999
## [28,]    1    1 -999 -999 -999 -999 -999 -999 -999  -999  -999  -999  -999
## [29,]    1    1 -999 -999 -999 -999 -999 -999 -999  -999  -999  -999  -999
## [30,]    1    1 -999 -999 -999 -999 -999 -999 -999  -999  -999  -999  -999
##       [,14]
##  [1,]  -999
##  [2,]  -999
##  [3,]  -999
##  [4,]  -999
##  [5,]  -999
##  [6,]  -999
##  [7,]  -999
##  [8,]  -999
##  [9,]  -999
## [10,]     1
## [11,]  -999
## [12,]  -999
## [13,]  -999
## [14,]  -999
## [15,]  -999
## [16,]     1
## [17,]  -999
## [18,]  -999
## [19,]  -999
## [20,]  -999
## [21,]  -999
## [22,]  -999
## [23,]  -999
## [24,]  -999
## [25,]  -999
## [26,]  -999
## [27,]  -999
## [28,]  -999
## [29,]  -999
## [30,]  -999
## 
## $t_RLB
##       [,1] [,2] [,3] [,4] [,5] [,6] [,7] [,8] [,9] [,10] [,11] [,12] [,13]
##  [1,]    1    1    0    1 -999 -999 -999 -999 -999  -999  -999  -999  -999
##  [2,]    0    1 -999 -999 -999 -999 -999 -999 -999  -999  -999  -999  -999
##  [3,]    1    0    0    1    1    1    1    1    1     1     0     0  -999
##  [4,]    1 -999 -999 -999 -999 -999 -999 -999 -999  -999  -999  -999  -999
##  [5,]    0    1    0 -999 -999 -999 -999 -999 -999  -999  -999  -999  -999
##  [6,]    0    0    1    0    1    1    0 -999 -999  -999  -999  -999  -999
##  [7,]    0    1    1    1    1    1    1    0    0     1     1  -999  -999
##  [8,]    1    0    1 -999 -999 -999 -999 -999 -999  -999  -999  -999  -999
##  [9,]    0    1    0 -999 -999 -999 -999 -999 -999  -999  -999  -999  -999
## [10,]    1    1    0    0    0    1    1    1    0     1     1     1     1
## [11,]    0    1    0    0    0    0    1 -999 -999  -999  -999  -999  -999
## [12,] -999 -999 -999 -999 -999 -999 -999 -999 -999  -999  -999  -999  -999
## [13,]    0    1    1    1 -999 -999 -999 -999 -999  -999  -999  -999  -999
## [14,]    0    0    0    0    0    0 -999 -999 -999  -999  -999  -999  -999
## [15,]    1    1    0    1 -999 -999 -999 -999 -999  -999  -999  -999  -999
## [16,]    0    1    1    1    0    1    0    1    1     1     0     1     1
## [17,]    0    1    1    1    1    0    1    0    0     1  -999  -999  -999
## [18,]    1    1    1    0    1    0    0    0    0     1     1     0     1
## [19,] -999 -999 -999 -999 -999 -999 -999 -999 -999  -999  -999  -999  -999
## [20,]    0    0    0    0    0    1    1 -999 -999  -999  -999  -999  -999
## [21,]    0    1 -999 -999 -999 -999 -999 -999 -999  -999  -999  -999  -999
## [22,]    0    1    0 -999 -999 -999 -999 -999 -999  -999  -999  -999  -999
## [23,]    1    0    1 -999 -999 -999 -999 -999 -999  -999  -999  -999  -999
## [24,]    1    1    1    0    0    1    0    1    0     1     0     1     1
## [25,]    1 -999 -999 -999 -999 -999 -999 -999 -999  -999  -999  -999  -999
## [26,]    0    0    0    0    0    0    1    0 -999  -999  -999  -999  -999
## [27,]    0    1    1    1    1    1 -999 -999 -999  -999  -999  -999  -999
## [28,]    0    1 -999 -999 -999 -999 -999 -999 -999  -999  -999  -999  -999
## [29,]    0    1 -999 -999 -999 -999 -999 -999 -999  -999  -999  -999  -999
## [30,]    0    0 -999 -999 -999 -999 -999 -999 -999  -999  -999  -999  -999
##       [,14]
##  [1,]  -999
##  [2,]  -999
##  [3,]  -999
##  [4,]  -999
##  [5,]  -999
##  [6,]  -999
##  [7,]  -999
##  [8,]  -999
##  [9,]  -999
## [10,]     1
## [11,]  -999
## [12,]  -999
## [13,]  -999
## [14,]  -999
## [15,]  -999
## [16,]     1
## [17,]  -999
## [18,]  -999
## [19,]  -999
## [20,]  -999
## [21,]  -999
## [22,]  -999
## [23,]  -999
## [24,]  -999
## [25,]  -999
## [26,]  -999
## [27,]  -999
## [28,]  -999
## [29,]  -999
## [30,]  -999
## 
## $n_noRLB
##            601            602            603            604            605 
##              4              4              3              3              4 
##            606            607            608            609            610 
##              4              3              1              1              1 
##            611            612            613            614            615 
##              1              1              0              5              0 
##            616            617            618            619            620 
##              5              3              5              1              2 
##            621            622            623            624            625 
##              5              0              1             10              3 
##            626            627            628            629            630 
##              3              1              2              1              1 
## 
## $z_noRLB
##       [,1] [,2] [,3] [,4] [,5] [,6] [,7] [,8] [,9] [,10]
##  [1,]    1    1    1    1 -999 -999 -999 -999 -999  -999
##  [2,]    1    1    1    1 -999 -999 -999 -999 -999  -999
##  [3,]    1    1    1 -999 -999 -999 -999 -999 -999  -999
##  [4,]    1    1    1 -999 -999 -999 -999 -999 -999  -999
##  [5,]    1    1    1    1 -999 -999 -999 -999 -999  -999
##  [6,]    1    1    1    1 -999 -999 -999 -999 -999  -999
##  [7,]    1    1    1 -999 -999 -999 -999 -999 -999  -999
##  [8,]    1 -999 -999 -999 -999 -999 -999 -999 -999  -999
##  [9,]    1 -999 -999 -999 -999 -999 -999 -999 -999  -999
## [10,]    1 -999 -999 -999 -999 -999 -999 -999 -999  -999
## [11,]    1 -999 -999 -999 -999 -999 -999 -999 -999  -999
## [12,]    1 -999 -999 -999 -999 -999 -999 -999 -999  -999
## [13,] -999 -999 -999 -999 -999 -999 -999 -999 -999  -999
## [14,]    1    1    1    1    1 -999 -999 -999 -999  -999
## [15,] -999 -999 -999 -999 -999 -999 -999 -999 -999  -999
## [16,]    1    1    1    1    1 -999 -999 -999 -999  -999
## [17,]    1    1    1 -999 -999 -999 -999 -999 -999  -999
## [18,]    1    1    1    1    1 -999 -999 -999 -999  -999
## [19,]    1 -999 -999 -999 -999 -999 -999 -999 -999  -999
## [20,]    1    1 -999 -999 -999 -999 -999 -999 -999  -999
## [21,]    1    1    1    1    1 -999 -999 -999 -999  -999
## [22,] -999 -999 -999 -999 -999 -999 -999 -999 -999  -999
## [23,]    1 -999 -999 -999 -999 -999 -999 -999 -999  -999
## [24,]    1    1    1    1    1    1    1    1    1     1
## [25,]    1    1    1 -999 -999 -999 -999 -999 -999  -999
## [26,]    1    1    1 -999 -999 -999 -999 -999 -999  -999
## [27,]    1 -999 -999 -999 -999 -999 -999 -999 -999  -999
## [28,]    1    1 -999 -999 -999 -999 -999 -999 -999  -999
## [29,]    1 -999 -999 -999 -999 -999 -999 -999 -999  -999
## [30,]    1 -999 -999 -999 -999 -999 -999 -999 -999  -999
## 
## $v_noRLB
##       [,1] [,2] [,3] [,4] [,5] [,6] [,7] [,8] [,9] [,10]
##  [1,]    0    0    0    0 -999 -999 -999 -999 -999  -999
##  [2,]    0    0    0    0 -999 -999 -999 -999 -999  -999
##  [3,]    0    0    0 -999 -999 -999 -999 -999 -999  -999
##  [4,]    0    0    0 -999 -999 -999 -999 -999 -999  -999
##  [5,]    0    0    0    0 -999 -999 -999 -999 -999  -999
##  [6,]    0    0    0    0 -999 -999 -999 -999 -999  -999
##  [7,]    0    0    0 -999 -999 -999 -999 -999 -999  -999
##  [8,]    0 -999 -999 -999 -999 -999 -999 -999 -999  -999
##  [9,]    0 -999 -999 -999 -999 -999 -999 -999 -999  -999
## [10,]    0 -999 -999 -999 -999 -999 -999 -999 -999  -999
## [11,]    0 -999 -999 -999 -999 -999 -999 -999 -999  -999
## [12,]    0 -999 -999 -999 -999 -999 -999 -999 -999  -999
## [13,] -999 -999 -999 -999 -999 -999 -999 -999 -999  -999
## [14,]    0    0    0    0    0 -999 -999 -999 -999  -999
## [15,] -999 -999 -999 -999 -999 -999 -999 -999 -999  -999
## [16,]    0    0    0    0    0 -999 -999 -999 -999  -999
## [17,]    0    0    0 -999 -999 -999 -999 -999 -999  -999
## [18,]    0    0    0    0    0 -999 -999 -999 -999  -999
## [19,]    0 -999 -999 -999 -999 -999 -999 -999 -999  -999
## [20,]    0    0 -999 -999 -999 -999 -999 -999 -999  -999
## [21,]    0    0    0    0    0 -999 -999 -999 -999  -999
## [22,] -999 -999 -999 -999 -999 -999 -999 -999 -999  -999
## [23,]    0 -999 -999 -999 -999 -999 -999 -999 -999  -999
## [24,]    0    0    0    0    0    0    0    0    0     0
## [25,]    0    0    0 -999 -999 -999 -999 -999 -999  -999
## [26,]    0    0    0 -999 -999 -999 -999 -999 -999  -999
## [27,]    0 -999 -999 -999 -999 -999 -999 -999 -999  -999
## [28,]    0    0 -999 -999 -999 -999 -999 -999 -999  -999
## [29,]    0 -999 -999 -999 -999 -999 -999 -999 -999  -999
## [30,]    0 -999 -999 -999 -999 -999 -999 -999 -999  -999
## 
## $t_noRLB
##       [,1] [,2] [,3] [,4] [,5] [,6] [,7] [,8] [,9] [,10]
##  [1,]   NA   NA   NA   NA -999 -999 -999 -999 -999  -999
##  [2,]   NA   NA   NA   NA -999 -999 -999 -999 -999  -999
##  [3,]   NA   NA   NA -999 -999 -999 -999 -999 -999  -999
##  [4,]   NA   NA   NA -999 -999 -999 -999 -999 -999  -999
##  [5,]   NA   NA   NA   NA -999 -999 -999 -999 -999  -999
##  [6,]   NA   NA   NA   NA -999 -999 -999 -999 -999  -999
##  [7,]   NA   NA   NA -999 -999 -999 -999 -999 -999  -999
##  [8,]   NA -999 -999 -999 -999 -999 -999 -999 -999  -999
##  [9,]   NA -999 -999 -999 -999 -999 -999 -999 -999  -999
## [10,]   NA -999 -999 -999 -999 -999 -999 -999 -999  -999
## [11,]   NA -999 -999 -999 -999 -999 -999 -999 -999  -999
## [12,]   NA -999 -999 -999 -999 -999 -999 -999 -999  -999
## [13,] -999 -999 -999 -999 -999 -999 -999 -999 -999  -999
## [14,]   NA   NA   NA   NA   NA -999 -999 -999 -999  -999
## [15,] -999 -999 -999 -999 -999 -999 -999 -999 -999  -999
## [16,]   NA   NA   NA   NA   NA -999 -999 -999 -999  -999
## [17,]   NA   NA   NA -999 -999 -999 -999 -999 -999  -999
## [18,]   NA   NA   NA   NA   NA -999 -999 -999 -999  -999
## [19,]   NA -999 -999 -999 -999 -999 -999 -999 -999  -999
## [20,]   NA   NA -999 -999 -999 -999 -999 -999 -999  -999
## [21,]   NA   NA   NA   NA   NA -999 -999 -999 -999  -999
## [22,] -999 -999 -999 -999 -999 -999 -999 -999 -999  -999
## [23,]   NA -999 -999 -999 -999 -999 -999 -999 -999  -999
## [24,]   NA   NA   NA   NA   NA   NA   NA   NA   NA    NA
## [25,]   NA   NA   NA -999 -999 -999 -999 -999 -999  -999
## [26,]   NA   NA   NA -999 -999 -999 -999 -999 -999  -999
## [27,]   NA -999 -999 -999 -999 -999 -999 -999 -999  -999
## [28,]   NA   NA -999 -999 -999 -999 -999 -999 -999  -999
## [29,]   NA -999 -999 -999 -999 -999 -999 -999 -999  -999
## [30,]   NA -999 -999 -999 -999 -999 -999 -999 -999  -999  
  jags.script.c <- "

model{ # realistic, RLB, alpha_2=gamma_2=0 (ignore emptyVariable)

  ########################### NOTE ##########################
  ##
  ## To reflect conditional binomials, partition n_i for
  ## each i into
  ##
  ##         n_i = n_i_noLyme + n_i_RLB + n_i_noRLB
  ##
  ## and similarly for the associated data vectors z_i, t_i, v_i.
  ## Thus, if the partition is irrelevant to the model statement
  ## (e.g. Eq. [2.2a]), then must use same distribution (with 
  ## same parameters) over all loop partitions under likelihood.
  ##
  ##############################################################
  
  # ---------- definitions
  
  tau <- 1/sqrt(tausq.inv) # SD in eq 2.3
  omega <- 1/sqrt(omsq.inv) # SD in eq 2.5
  alph[2] <- 0
  gam[2] <- 0
  
  for(i in 1:S){

    pB[i] <- ilogit(logitpB[i])
    pC[i] <- ilogit(logitpC[i])
    pS[i] <- (1-pC[i]/pFH[i])/(1-pSH[i]/pFH[i]) # eq 2.7
    logitpS[i] <- logit(pS[i])
    logitpSH[i] <- logit(pSH[i])
    logitpFH[i] <- logit(pFH[i])

    nuL[i] <- alph0 + alph[1] * x[i,1] + inprod(alph[3:5], x[i,3:5]) # mean in eq 2.3
    nuH[i] <- gam0 + gam[1] * x[i,1] + inprod(gam[3:5], x[i,3:5]) # mean in eq 2.5     
  }
    
  # ---------- likelihood
  
  for(i in 1:S){
    
    logitpB[i] ~ dnorm(nuL[i], tausq.inv) # eq 2.3    
    logitpC[i] ~ dnorm(nuH[i], omsq.inv) # eq 2.5

    for(j in 1:n_noLyme[i]){ # z=0

      z_noLyme[i,j] ~ dbern(pB[i]) # eq 2.2 for PCR-
    }
  }

  for(i in 1:12){ # z=1, v=0

    for(j in 1:n_noRLB[i]){
      z_noRLB[i,j] ~ dbern(pB[i]) # eq 2.2 for PCR+, v[i,j] = 0
      v_noRLB[i,j] ~ dbern(pS[i]) # eq 2.6 for PCR+, v[i,j] = 0
      t_noRLB[i,j] ~ dbern(pFH[i]) # eq 2.4c1
    }
  }    

  for(i in 14:14){ # z=1, v=0

    for(j in 1:n_noRLB[i]){
      z_noRLB[i,j] ~ dbern(pB[i]) # eq 2.2 for PCR+, v[i,j] = 0
      v_noRLB[i,j] ~ dbern(pS[i]) # eq 2.6 for PCR+, v[i,j] = 0
      t_noRLB[i,j] ~ dbern(pFH[i]) # eq 2.4c1
    }
  }    

  for(i in 16:21){ # z=1, v=0

    for(j in 1:n_noRLB[i]){
      z_noRLB[i,j] ~ dbern(pB[i]) # eq 2.2 for PCR+, v[i,j] = 0
      v_noRLB[i,j] ~ dbern(pS[i]) # eq 2.6 for PCR+, v[i,j] = 0
      t_noRLB[i,j] ~ dbern(pFH[i]) # eq 2.4c1
    }
  }    

  for(i in 23:S){ # z=1, v=0

    for(j in 1:n_noRLB[i]){
      z_noRLB[i,j] ~ dbern(pB[i]) # eq 2.2 for PCR+, v[i,j] = 0
      v_noRLB[i,j] ~ dbern(pS[i]) # eq 2.6 for PCR+, v[i,j] = 0
      t_noRLB[i,j] ~ dbern(pFH[i]) # eq 2.4c1
    }
  }    

  for(i in 1:11){ # z=v=1

    for(j in 1:n_RLB[i]){

        z_RLB[i,j] ~ dbern(pB[i]) # eq 2.2 for PCR+, v[i,j] = 1
        v_RLB[i,j] ~ dbern(pS[i]) # eq 2.6 for PCR+, v[i,j] = 1
        t_RLB[i,j] ~ dbern(pSH[i]) # eq 2.4c
    }    
  }

  for(i in 13:18){ # z=v=1

    for(j in 1:n_RLB[i]){

        z_RLB[i,j] ~ dbern(pB[i]) # eq 2.2 for PCR+, v[i,j] = 1
        v_RLB[i,j] ~ dbern(pS[i]) # eq 2.6 for PCR+, v[i,j] = 1
        t_RLB[i,j] ~ dbern(pSH[i]) # eq 2.4c
    }    
  }

  for(i in 20:S){ # z=v=1

    for(j in 1:n_RLB[i]){

        z_RLB[i,j] ~ dbern(pB[i]) # eq 2.2 for PCR+, v[i,j] = 1
        v_RLB[i,j] ~ dbern(pS[i]) # eq 2.6 for PCR+, v[i,j] = 1
        t_RLB[i,j] ~ dbern(pSH[i]) # eq 2.4c
    }    
  }

  # ---------- priors
  
  tausq.inv ~ dgamma(1, .01)
  omsq.inv ~ dgamma(1, .01)

  alph0 ~ dnorm(0, .001)
  gam0 ~ dnorm(0, .001)
  alph[1] ~ dnorm(0, .001)
  gam[1] ~ dnorm(0, .001)

  for(k in 3:5){
    
    alph[k] ~ dnorm(0, .001)
    gam[k] ~ dnorm(0, .001)
        
  }

  for(i in 1:S){

    pSH[i] ~ dunif(0, pC[i])
    pFH[i] ~ dunif(pC[i], 1)
  }
}
"  
  fit.c <- run.jags(jags.script.c, data=dat.c, n.chains=2,
                      inits=list(tausq.inv=1, omsq.inv=1),
                      adapt=10000, burnin=5000, sample=5000, thin=10,
                      monitor=c(
                        "t_noRLB[1,1]","t_noRLB[1,2]","t_noRLB[1,3]","t_noRLB[1,4]",
                        "t_noRLB[2,1]","t_noRLB[2,2]","t_noRLB[2,3]","t_noRLB[2,4]",
                        "t_noRLB[3,1]","t_noRLB[3,2]","t_noRLB[3,3]",
                        "t_noRLB[4,1]","t_noRLB[4,2]","t_noRLB[4,3]",
                        "t_noRLB[5,1]","t_noRLB[5,2]","t_noRLB[5,3]","t_noRLB[5,4]",
                        "t_noRLB[6,1]","t_noRLB[6,2]","t_noRLB[6,3]","t_noRLB[6,4]",
                        "t_noRLB[7,1]","t_noRLB[7,2]","t_noRLB[7,3]",
                        "t_noRLB[8,1]",
                        "t_noRLB[9,1]",
                        "t_noRLB[10,1]",
                        "t_noRLB[11,1]",
                        "t_noRLB[12,1]",
                        "t_noRLB[14,1]","t_noRLB[14,2]","t_noRLB[14,3]","t_noRLB[14,4]","t_noRLB[14,5]",
                        "t_noRLB[16,1]","t_noRLB[16,2]","t_noRLB[16,3]","t_noRLB[16,4]","t_noRLB[16,5]",
                        "t_noRLB[17,1]","t_noRLB[17,2]","t_noRLB[17,3]",
                        "t_noRLB[18,1]","t_noRLB[18,2]","t_noRLB[18,3]","t_noRLB[18,4]","t_noRLB[18,5]",
                        "t_noRLB[19,1]",
                        "t_noRLB[20,1]","t_noRLB[20,2]",
                        "t_noRLB[21,1]","t_noRLB[21,2]","t_noRLB[21,3]","t_noRLB[21,4]","t_noRLB[21,5]",
                        "t_noRLB[23,1]",
                        "t_noRLB[24,1]","t_noRLB[24,2]","t_noRLB[24,3]","t_noRLB[24,4]","t_noRLB[24,5]",
                        "t_noRLB[24,6]","t_noRLB[24,7]","t_noRLB[24,8]","t_noRLB[24,9]","t_noRLB[24,10]",
                        "t_noRLB[25,1]","t_noRLB[25,2]","t_noRLB[25,3]",
                        "t_noRLB[26,1]","t_noRLB[26,2]","t_noRLB[26,3]",
                        "t_noRLB[27,1]",
                        "t_noRLB[28,1]","t_noRLB[28,2]",
                        "t_noRLB[29,1]",
                        "t_noRLB[30,1]",
                        "logitpB","logitpC","logitpS","logitpSH","logitpFH",
                        "alph0","alph","gam0","gam","tau","omega","deviance",
                        "pd","dic"))  
  ## module dic loaded
## Compiling rjags model...
## Calling the simulation using the rjags method...
## Adapting the model for 10000 iterations...
##   |++++++++++++++++++++++++++++++++++++++++++++++++++| 100%
## Burning in the model for 5000 iterations...
##   |**************************************************| 100%
## Running the model for 50000 iterations...
##   |**************************************************| 100%
## Extending 50000 iterations for pD/DIC estimates...
##   |**************************************************| 100%
## Simulation complete
## Calculating summary statistics...
## Note: The monitored variables 'alph[2]' and 'gam[2]' appear to be non-stochastic; they will not be included in the
## convergence diagnostic
## Calculating the Gelman-Rubin statistic for 243 variables....
## Finished running the simulation
## Warning messages:
## 1: The length of the initial values argument supplied found does not correspond to the number of chains specified.  Some initial values were recycled or ignored. 
## 2: In rjags::jags.model(model, data = dataenv, inits = inits, n.chains = length(runjags.object$end.state),  :
##   Unused variable "t_noLyme" in data
## 3: In rjags::jags.samples(rjags, variable.names = monitor, n.iter = extra.options$sample,  :
##   Failed to set trace monitor for pD
## pD is infinite because at least one observed node does not have fixed support  
 See  this  for discussion on infinite  pD . 
  scans.c <- as.mcmc.list(fit.c)
scans.c.pooled <- rbind(scans.c[[1]], scans.c[[2]])  
  fit.c$runjags.version  
  ## [1] "2.0.2-8"                      "R version 3.2.2 (2015-08-14)"
## [3] "unix"                         "RStudio"                     
## [5] "mac.binary.mavericks"         "2015-09-20 17:07:51"  
  fit.c$psrf$mpsrf # Gelman-Rubin convergence check  
  ## [1] 1.03492  
  fit.c.dic.alt <- mean(scans.c.pooled[,"deviance"]) + var(scans.c.pooled[,"deviance"])/2
fit.c.dic.alt  
  ## [1] 1511.262  
  pval <- apply(scans.c.pooled[,c(230,232:234,236,238:240)], 2, ecdf) # placeholder
pval <- sapply(pval,do.call,args=list(0))
pval <- data.frame(lefttail=pval,righttail=1-pval,
                      median=apply(scans.cNoS.pooled[,c(230,232:234,236,238:240)], 2, median))
tmp <- subset(pval,lefttail<.5)
pval <- rbind(tmp, subset(pval,righttail<.5))

print(pval)  # posterior probs of   0 (righttail) -- tails sum to 1  
  ##         lefttail righttail      median
## alph[3]   0.0370    0.9630  0.52465413
## alph[4]   0.1062    0.8938  0.09940828
## alph[5]   0.0993    0.9007  0.12994497
## gam[1]    0.2579    0.7421  0.20047632
## gam[5]    0.0461    0.9539  0.25656229
## alph[1]   0.5721    0.4279 -0.04478082
## gam[3]    0.9614    0.0386 -0.72719620
## gam[4]    0.6976    0.3024 -0.06117828  
  par(mfrow=c(4,3))
traceplot(scans.c)  
                                           
   
 Note with  dgamma(1, .001)  then larger DIC and poorer mixing (i.e., not our model of choice): 
  ## > fit.c.dic.alt # with dgamma(1, .001)
## [1] 1558.504  
   
     
 
 
 
 Reduced realistic model (as if HIS missingness is unrelated to any mechanism, i.e. failure data ignored) 
 This parametrization allows easy JAGS coding without the need to partition { \((i,j)\) } into { \((i,j)\) :  \(t_{i,j}\)  observed} and { \((i,j)\) :  \(t_{i,j}\)  unobserved}. 
  \[  
\begin{aligned}  
\text{[2.2a]} &&  
\left. z_{ij} \right| p_i \sim \text{(ind) Bernoulli}(p_i^B) \\  
\text{[2.3]} &&  
\left. \text{logit } p_i^B \right| \alpha_0, \boldsymbol{\alpha}, \tau^2, \boldsymbol{x}_i \sim \text{(ind) } N(\alpha_0 + \boldsymbol{x}_i ' \boldsymbol{\alpha}, \tau^2) \\  
\text{[3.1aa]} &&  
\left. t_{ij} \right| z_{ij}, p_i^c \sim \text{(ind) Bernoulli}(z_{ij} p_i^c) \\  
\text{[2.5]} &&  
\text{logit } p_i^c \left| \gamma_0, \boldsymbol{\gamma}, \omega^2, \boldsymbol{x}_i \right. \sim \text{(ind) } N(\gamma_0 + \boldsymbol{x}_i ' \boldsymbol{\gamma}, \omega^2) \\  
\end{aligned}  
\]  
  print(dat.a)  
  ## $S
## [1] 30
## 
## $n
##  [1] 22 14 44 13 16 22 36 32  8 32 17  2 11 22 31 39 34 46 20 31 33 16 10
## [24] 41 28 35 28 30 11 16
## 
## $z
##       [,1] [,2] [,3] [,4] [,5] [,6] [,7] [,8] [,9] [,10] [,11] [,12] [,13]
##  [1,]    1    0    0    1    0    1    0    0    1     0     0     0     0
##  [2,]    1    0    0    0    0    1    0    1    1     0     0     0     1
##  [3,]    0    1    0    0    0    0    0    1    0     1     0     1     1
##  [4,]    1    1    0    0    0    0    0    1    0     0     0     0     1
##  [5,]    0    1    1    0    1    0    0    1    1     0     0     1     0
##  [6,]    0    0    1    1    0    1    1    1    1     0     1     1     0
##  [7,]    0    0    1    0    0    0    0    0    0     1     1     0     0
##  [8,]    0    0    0    0    0    1    1    0    0     1     0     0     0
##  [9,]    1    1    0    1    0    0    1    0 -999  -999  -999  -999  -999
## [10,]    1    0    0    0    1    1    0    1    1     0     0     0     1
## [11,]    1    0    1    1    0    1    0    0    1     0     0     1     1
## [12,]    0    1 -999 -999 -999 -999 -999 -999 -999  -999  -999  -999  -999
## [13,]    1    1    1    0    0    0    1    0    0     0     0  -999  -999
## [14,]    0    0    0    0    0    1    0    1    1     1     1     0     0
## [15,]    0    0    0    1    0    0    0    0    0     1     0     0     0
## [16,]    0    0    1    0    1    0    0    1    1     1     1     0     0
## [17,]    0    1    0    1    0    0    1    0    1     0     1     1     0
## [18,]    0    0    1    0    0    0    0    0    0     0     1     1     0
## [19,]    0    0    1    0    0    0    0    0    0     0     0     0     0
## [20,]    0    1    0    0    0    0    0    1    0     0     1     0     0
## [21,]    0    1    0    0    0    0    0    0    0     0     0     0     1
## [22,]    0    0    0    0    0    0    0    0    1     1     0     0     1
## [23,]    0    0    0    0    0    1    1    1    0     1  -999  -999  -999
## [24,]    0    0    1    0    1    1    1    1    0     1     1     0     1
## [25,]    0    0    0    1    0    0    0    0    0     0     0     1     0
## [26,]    0    0    1    0    0    0    0    0    0     0     0     0     0
## [27,]    0    0    0    0    0    0    0    0    0     0     0     1     0
## [28,]    0    0    1    0    0    0    0    0    0     0     0     0     0
## [29,]    1    0    0    0    0    0    1    1    0     0     0  -999  -999
## [30,]    0    0    0    0    0    0    1    0    0     1     0     1     0
##       [,14] [,15] [,16] [,17] [,18] [,19] [,20] [,21] [,22] [,23] [,24]
##  [1,]     0     0     1     1     0     0     0     1     1  -999  -999
##  [2,]     1  -999  -999  -999  -999  -999  -999  -999  -999  -999  -999
##  [3,]     0     0     0     1     0     0     0     1     0     0     0
##  [4,]  -999  -999  -999  -999  -999  -999  -999  -999  -999  -999  -999
##  [5,]     0     0     1  -999  -999  -999  -999  -999  -999  -999  -999
##  [6,]     0     1     1     0     1     0     0     0     0  -999  -999
##  [7,]     1     1     1     0     0     1     1     0     0     0     1
##  [8,]     0     0     0     0     1     0     0     0     0     0     0
##  [9,]  -999  -999  -999  -999  -999  -999  -999  -999  -999  -999  -999
## [10,]     1     0     1     1     0     1     0     1     0     0     1
## [11,]     1     0     0     0  -999  -999  -999  -999  -999  -999  -999
## [12,]  -999  -999  -999  -999  -999  -999  -999  -999  -999  -999  -999
## [13,]  -999  -999  -999  -999  -999  -999  -999  -999  -999  -999  -999
## [14,]     0     1     1     1     1     0     0     1     1  -999  -999
## [15,]     0     1     0     0     0     0     0     0     0     0     0
## [16,]     0     0     0     0     1     1     0     0     1     0     1
## [17,]     0     0     0     1     0     0     0     1     0     1     1
## [18,]     0     0     0     0     1     1     0     1     1     0     1
## [19,]     0     0     0     0     0     0     0  -999  -999  -999  -999
## [20,]     0     0     0     1     1     0     0     0     0     1     0
## [21,]     0     0     0     0     1     0     0     1     0     0     0
## [22,]     0     0     0  -999  -999  -999  -999  -999  -999  -999  -999
## [23,]  -999  -999  -999  -999  -999  -999  -999  -999  -999  -999  -999
## [24,]     1     1     0     1     1     1     1     0     0     0     0
## [25,]     0     0     0     0     0     1     0     0     0     0     0
## [26,]     1     0     0     0     0     1     1     0     0     0     1
## [27,]     0     1     0     0     1     1     0     0     0     0     1
## [28,]     0     0     0     0     0     0     1     0     1     0     0
## [29,]  -999  -999  -999  -999  -999  -999  -999  -999  -999  -999  -999
## [30,]     0     0     0  -999  -999  -999  -999  -999  -999  -999  -999
##       [,25] [,26] [,27] [,28] [,29] [,30] [,31] [,32] [,33] [,34] [,35]
##  [1,]  -999  -999  -999  -999  -999  -999  -999  -999  -999  -999  -999
##  [2,]  -999  -999  -999  -999  -999  -999  -999  -999  -999  -999  -999
##  [3,]     1     0     0     1     0     0     1     0     1     0     1
##  [4,]  -999  -999  -999  -999  -999  -999  -999  -999  -999  -999  -999
##  [5,]  -999  -999  -999  -999  -999  -999  -999  -999  -999  -999  -999
##  [6,]  -999  -999  -999  -999  -999  -999  -999  -999  -999  -999  -999
##  [7,]     1     1     0     0     0     1     0     1     0     1     0
##  [8,]     0     0     0     0     0     0     0     0  -999  -999  -999
##  [9,]  -999  -999  -999  -999  -999  -999  -999  -999  -999  -999  -999
## [10,]     1     0     0     1     0     0     0     1  -999  -999  -999
## [11,]  -999  -999  -999  -999  -999  -999  -999  -999  -999  -999  -999
## [12,]  -999  -999  -999  -999  -999  -999  -999  -999  -999  -999  -999
## [13,]  -999  -999  -999  -999  -999  -999  -999  -999  -999  -999  -999
## [14,]  -999  -999  -999  -999  -999  -999  -999  -999  -999  -999  -999
## [15,]     0     0     0     0     1     0     0  -999  -999  -999  -999
## [16,]     1     0     0     0     1     1     1     1     0     0     1
## [17,]     1     0     1     0     0     0     0     0     1     0  -999
## [18,]     1     1     1     1     1     0     0     0     1     0     0
## [19,]  -999  -999  -999  -999  -999  -999  -999  -999  -999  -999  -999
## [20,]     0     0     0     1     0     1     1  -999  -999  -999  -999
## [21,]     0     1     0     0     0     1     0     1     0  -999  -999
## [22,]  -999  -999  -999  -999  -999  -999  -999  -999  -999  -999  -999
## [23,]  -999  -999  -999  -999  -999  -999  -999  -999  -999  -999  -999
## [24,]     1     0     1     1     0     1     0     0     0     1     1
## [25,]     1     0     0     0  -999  -999  -999  -999  -999  -999  -999
## [26,]     1     0     0     1     0     1     1     0     0     1     1
## [27,]     1     0     1     0  -999  -999  -999  -999  -999  -999  -999
## [28,]     1     0     0     0     0     0  -999  -999  -999  -999  -999
## [29,]  -999  -999  -999  -999  -999  -999  -999  -999  -999  -999  -999
## [30,]  -999  -999  -999  -999  -999  -999  -999  -999  -999  -999  -999
##       [,36] [,37] [,38] [,39] [,40] [,41] [,42] [,43] [,44] [,45] [,46]
##  [1,]  -999  -999  -999  -999  -999  -999  -999  -999  -999  -999  -999
##  [2,]  -999  -999  -999  -999  -999  -999  -999  -999  -999  -999  -999
##  [3,]     0     0     0     0     0     0     1     1     1  -999  -999
##  [4,]  -999  -999  -999  -999  -999  -999  -999  -999  -999  -999  -999
##  [5,]  -999  -999  -999  -999  -999  -999  -999  -999  -999  -999  -999
##  [6,]  -999  -999  -999  -999  -999  -999  -999  -999  -999  -999  -999
##  [7,]     0  -999  -999  -999  -999  -999  -999  -999  -999  -999  -999
##  [8,]  -999  -999  -999  -999  -999  -999  -999  -999  -999  -999  -999
##  [9,]  -999  -999  -999  -999  -999  -999  -999  -999  -999  -999  -999
## [10,]  -999  -999  -999  -999  -999  -999  -999  -999  -999  -999  -999
## [11,]  -999  -999  -999  -999  -999  -999  -999  -999  -999  -999  -999
## [12,]  -999  -999  -999  -999  -999  -999  -999  -999  -999  -999  -999
## [13,]  -999  -999  -999  -999  -999  -999  -999  -999  -999  -999  -999
## [14,]  -999  -999  -999  -999  -999  -999  -999  -999  -999  -999  -999
## [15,]  -999  -999  -999  -999  -999  -999  -999  -999  -999  -999  -999
## [16,]     1     0     1     1  -999  -999  -999  -999  -999  -999  -999
## [17,]  -999  -999  -999  -999  -999  -999  -999  -999  -999  -999  -999
## [18,]     0     1     1     0     0     1     0     0     0     0     1
## [19,]  -999  -999  -999  -999  -999  -999  -999  -999  -999  -999  -999
## [20,]  -999  -999  -999  -999  -999  -999  -999  -999  -999  -999  -999
## [21,]  -999  -999  -999  -999  -999  -999  -999  -999  -999  -999  -999
## [22,]  -999  -999  -999  -999  -999  -999  -999  -999  -999  -999  -999
## [23,]  -999  -999  -999  -999  -999  -999  -999  -999  -999  -999  -999
## [24,]     0     1     0     1     1     0  -999  -999  -999  -999  -999
## [25,]  -999  -999  -999  -999  -999  -999  -999  -999  -999  -999  -999
## [26,]  -999  -999  -999  -999  -999  -999  -999  -999  -999  -999  -999
## [27,]  -999  -999  -999  -999  -999  -999  -999  -999  -999  -999  -999
## [28,]  -999  -999  -999  -999  -999  -999  -999  -999  -999  -999  -999
## [29,]  -999  -999  -999  -999  -999  -999  -999  -999  -999  -999  -999
## [30,]  -999  -999  -999  -999  -999  -999  -999  -999  -999  -999  -999
## 
## $t
##                [,1] [,2] [,3] [,4] [,5] [,6] [,7] [,8] [,9] [,10] [,11]
##      601          1    0    0    1    0   NA    0    0   NA     0     0
##      602         NA    0    0    0    0    0    0   NA   NA     0     0
##      603          0    1    0    0    0    0    0    0    0    NA     0
##      604         NA    1    0    0    0    0    0   NA    0     0     0
##      605          0    0    1    0   NA    0    0   NA   NA     0     0
##      606          0    0    0   NA    0    0    1   NA    0     0     1
##      607          0    0   NA    0    0    0    0    0    0    NA     0
##      608          0    0    0    0    0   NA    1    0    0     0     0
##      609          0    1    0   NA    0    0    0    0 -999  -999  -999
##      610          1    0    0    0    1    0    0    0    0     0     0
##      611          0    0    1    0    0    0    0    0    0     0     0
##      612          0   NA -999 -999 -999 -999 -999 -999 -999  -999  -999
##      613          0    1    1    0    0    0    1    0    0     0     0
##      614          0    0    0    0    0   NA    0    0    0     0    NA
##      615          0    0    0    1    0    0    0    0    0     1     0
##      616          0    0    0    0    1    0    0    1   NA     1     0
##      617          0    0    0   NA    0    0    1    0    1     0     1
##      618          0    0    1    0    0    0    0    0    0     0    NA
##      619          0    0   NA    0    0    0    0    0    0     0     0
##      620          0    0    0    0    0    0    0    0    0     0    NA
##      621          0    0    0    0    0    0    0    0    0     0     0
##      622          0    0    0    0    0    0    0    0    0     1     0
##      623          0    0    0    0    0    1   NA    0    0     1  -999
##      624          0    0   NA    0   NA   NA    1    1    0     1    NA
##      625          0    0    0    1    0    0    0    0    0     0     0
##      626          0    0    0    0    0    0    0    0    0     0     0
##      627          0    0    0    0    0    0    0    0    0     0     0
##      628          0    0   NA    0    0    0    0    0    0     0     0
##      629         NA    0    0    0    0    0    0    1    0     0     0
##      630          0    0    0    0    0    0    0    0    0    NA     0
##                [,12] [,13] [,14] [,15] [,16] [,17] [,18] [,19] [,20] [,21]
##      601           0     0     0     0     0    NA     0     0     0     1
##      602           0    NA     1  -999  -999  -999  -999  -999  -999  -999
##      603          NA     0     0     0     0     1     0     0     0    NA
##      604           0    NA  -999  -999  -999  -999  -999  -999  -999  -999
##      605           0     0     0     0    NA  -999  -999  -999  -999  -999
##      606          NA     0     0     1    NA     0     0     0     0     0
##      607           0     0     1    NA     1     0     0     1     1     0
##      608           0     0     0     0     0     0     1     0     0     0
##      609        -999  -999  -999  -999  -999  -999  -999  -999  -999  -999
##      610           0     1     1     0     1     0     0     1     0     1
##      611           0     1    NA     0     0     0  -999  -999  -999  -999
##      612        -999  -999  -999  -999  -999  -999  -999  -999  -999  -999
##      613        -999  -999  -999  -999  -999  -999  -999  -999  -999  -999
##      614           0     0     0    NA     0    NA    NA     0     0     0
##      615           0     0     0     0     0     0     0     0     0     0
##      616           0     0     0     0     0     0    NA     1     0     0
##      617          NA     0     0     0     0     1     0     0     0     0
##      618           1     0     0     0     0     0     1     0     0    NA
##      619           0     0     0     0     0     0     0     0     0  -999
##      620           0     0     0     0     0     0     0     0     0     0
##      621           0     1     0     0     0     0    NA     0     0    NA
##      622           0     0     0     0     0  -999  -999  -999  -999  -999
##      623        -999  -999  -999  -999  -999  -999  -999  -999  -999  -999
##      624           0     0     0    NA     0     1     0     1     0     0
##      625          NA     0     0     0     0     0     0    NA     0     0
##      626           0     0     0     0     0     0     0    NA     0     0
##      627           0     0     0     1     0     0     1     1     0     0
##      628           0     0     0     0     0     0     0     0    NA     0
##      629        -999  -999  -999  -999  -999  -999  -999  -999  -999  -999
##      630           0     0     0     0     0  -999  -999  -999  -999  -999
##                [,22] [,23] [,24] [,25] [,26] [,27] [,28] [,29] [,30] [,31]
##      601          NA  -999  -999  -999  -999  -999  -999  -999  -999  -999
##      602        -999  -999  -999  -999  -999  -999  -999  -999  -999  -999
##      603           0     0     0     1     0     0     1     0     0     1
##      604        -999  -999  -999  -999  -999  -999  -999  -999  -999  -999
##      605        -999  -999  -999  -999  -999  -999  -999  -999  -999  -999
##      606           0  -999  -999  -999  -999  -999  -999  -999  -999  -999
##      607           0     0     1     1     0     0     0     0     0     0
##      608           0     0     0     0     0     0     0     0     0     0
##      609        -999  -999  -999  -999  -999  -999  -999  -999  -999  -999
##      610           0     0     1     1     0     0     1     0     0     0
##      611        -999  -999  -999  -999  -999  -999  -999  -999  -999  -999
##      612        -999  -999  -999  -999  -999  -999  -999  -999  -999  -999
##      613        -999  -999  -999  -999  -999  -999  -999  -999  -999  -999
##      614           0  -999  -999  -999  -999  -999  -999  -999  -999  -999
##      615           0     0     0     0     0     0     0     1     0     0
##      616          NA     0     0     1     0     0     0     1    NA     1
##      617           0     1    NA     0     0     0     0     0     0     0
##      618           1     0     0     0    NA     0    NA    NA     0     0
##      619        -999  -999  -999  -999  -999  -999  -999  -999  -999  -999
##      620           0    NA     0     0     0     0     0     0     1     1
##      621           0     0     0     0    NA     0     0     0    NA     0
##      622        -999  -999  -999  -999  -999  -999  -999  -999  -999  -999
##      623        -999  -999  -999  -999  -999  -999  -999  -999  -999  -999
##      624           0     0     0     1     0    NA     0     0     1     0
##      625           0     0     0    NA     0     0     0  -999  -999  -999
##      626           0     0     0     0     0     0     0     0    NA    NA
##      627           0     0     1     1     0    NA     0  -999  -999  -999
##      628           0     0     0     1     0     0     0     0     0  -999
##      629        -999  -999  -999  -999  -999  -999  -999  -999  -999  -999
##      630        -999  -999  -999  -999  -999  -999  -999  -999  -999  -999
##                [,32] [,33] [,34] [,35] [,36] [,37] [,38] [,39] [,40] [,41]
##      601        -999  -999  -999  -999  -999  -999  -999  -999  -999  -999
##      602        -999  -999  -999  -999  -999  -999  -999  -999  -999  -999
##      603           0     1     0     1     0     0     0     0     0     0
##      604        -999  -999  -999  -999  -999  -999  -999  -999  -999  -999
##      605        -999  -999  -999  -999  -999  -999  -999  -999  -999  -999
##      606        -999  -999  -999  -999  -999  -999  -999  -999  -999  -999
##      607           1     0     1     0     0  -999  -999  -999  -999  -999
##      608           0  -999  -999  -999  -999  -999  -999  -999  -999  -999
##      609        -999  -999  -999  -999  -999  -999  -999  -999  -999  -999
##      610          NA  -999  -999  -999  -999  -999  -999  -999  -999  -999
##      611        -999  -999  -999  -999  -999  -999  -999  -999  -999  -999
##      612        -999  -999  -999  -999  -999  -999  -999  -999  -999  -999
##      613        -999  -999  -999  -999  -999  -999  -999  -999  -999  -999
##      614        -999  -999  -999  -999  -999  -999  -999  -999  -999  -999
##      615        -999  -999  -999  -999  -999  -999  -999  -999  -999  -999
##      616           0     0     0     1    NA     0     1     1  -999  -999
##      617           0     1     0  -999  -999  -999  -999  -999  -999  -999
##      618           0     0     0     0     0     1     1     0     0     0
##      619        -999  -999  -999  -999  -999  -999  -999  -999  -999  -999
##      620        -999  -999  -999  -999  -999  -999  -999  -999  -999  -999
##      621          NA     0  -999  -999  -999  -999  -999  -999  -999  -999
##      622        -999  -999  -999  -999  -999  -999  -999  -999  -999  -999
##      623        -999  -999  -999  -999  -999  -999  -999  -999  -999  -999
##      624           0     0     1    NA     0    NA     0    NA    NA     0
##      625        -999  -999  -999  -999  -999  -999  -999  -999  -999  -999
##      626           0     0     1     0  -999  -999  -999  -999  -999  -999
##      627        -999  -999  -999  -999  -999  -999  -999  -999  -999  -999
##      628        -999  -999  -999  -999  -999  -999  -999  -999  -999  -999
##      629        -999  -999  -999  -999  -999  -999  -999  -999  -999  -999
##      630        -999  -999  -999  -999  -999  -999  -999  -999  -999  -999
##                [,42] [,43] [,44] [,45] [,46]
##      601        -999  -999  -999  -999  -999
##      602        -999  -999  -999  -999  -999
##      603           1     0     0  -999  -999
##      604        -999  -999  -999  -999  -999
##      605        -999  -999  -999  -999  -999
##      606        -999  -999  -999  -999  -999
##      607        -999  -999  -999  -999  -999
##      608        -999  -999  -999  -999  -999
##      609        -999  -999  -999  -999  -999
##      610        -999  -999  -999  -999  -999
##      611        -999  -999  -999  -999  -999
##      612        -999  -999  -999  -999  -999
##      613        -999  -999  -999  -999  -999
##      614        -999  -999  -999  -999  -999
##      615        -999  -999  -999  -999  -999
##      616        -999  -999  -999  -999  -999
##      617        -999  -999  -999  -999  -999
##      618           0     0     0     0     1
##      619        -999  -999  -999  -999  -999
##      620        -999  -999  -999  -999  -999
##      621        -999  -999  -999  -999  -999
##      622        -999  -999  -999  -999  -999
##      623        -999  -999  -999  -999  -999
##      624        -999  -999  -999  -999  -999
##      625        -999  -999  -999  -999  -999
##      626        -999  -999  -999  -999  -999
##      627        -999  -999  -999  -999  -999
##      628        -999  -999  -999  -999  -999
##      629        -999  -999  -999  -999  -999
##      630        -999  -999  -999  -999  -999
## 
## $x
##                hostHindexAll emptyVar log_hostPELEprop log_hostTASTprop
##      601         -0.15313333              -0.386414919      -2.77860025
##      602         -0.08213333              -0.011334547       0.53194277
##      603          0.59586667              -0.405165468       1.45260350
##      604         -0.39013333              -0.048175117       0.33934966
##      605          0.04486667               0.341716818      -2.77860025
##      606         -1.41713333               0.544016842      -2.77860025
##      607          0.73686667              -0.258705394       1.39270536
##      608          0.01186667               0.062469427      -2.77860025
##      609         -0.13613333              -0.059274010       2.01055678
##      610          0.67486667               0.163869542       0.47949629
##      611         -0.21713333               0.459997986      -0.86167763
##      612         -0.08413333              -0.017737984       1.14929611
##      613         -0.16713333              -0.272423030       1.54155098
##      614         -0.20913333               0.387013093       1.67574705
##      615          0.06086667               0.195506627      -0.04423274
##      616          0.36086667               0.268872851       0.78244584
##      617          0.66686667               0.247751026      -0.21365089
##      618          0.30586667               0.311365475       0.94949992
##      619          0.33586667               0.188562154       0.03080245
##      620          0.56186667               0.127501898       0.24669083
##      621         -0.35113333              -0.518620344       1.19921050
##      622          0.69686667              -0.275189284       0.46399211
##      623         -0.31313333              -0.323425585       1.12941474
##      624          0.38586667              -0.075022367       0.32149204
##      625          0.21386667               0.278472924      -2.77860025
##      626         -0.18013333               0.458669082      -2.77860025
##      627         -0.37313333               0.249391715       1.56520518
##      628         -0.91313333              -2.036932857       1.85806861
##      629         -0.36913333               0.408215301      -2.77860025
##      630         -0.29713333              -0.004971854       1.44969229
##                log_hostBLBRprop
##      601             0.83156557
##      602             0.90700528
##      603            -0.68188397
##      604             0.52884562
##      605            -0.31677015
##      606             0.21731233
##      607             0.48760266
##      608             0.16055952
##      609             0.73364003
##      610             0.45551435
##      611            -0.28208460
##      612            -0.71637014
##      613             0.72951632
##      614            -0.46896197
##      615             0.21731233
##      616             0.02807033
##      617             0.39387018
##      618             1.09565051
##      619            -3.14998350
##      620             0.52378232
##      621            -0.27078504
##      622            -0.19507322
##      623             1.29501794
##      624             0.45551435
##      625             0.76603153
##      626            -3.14998350
##      627             0.20342322
##      628            -3.14998350
##      629             0.73774682
##      630             1.61389838  
  jags.script.aa <- "

model{ # same as 'c' except ignore RLB failure, alpha_2=gamma_2=0 (ignore emptyVar)
  
  # ---------- definitions
  
  tau <- 1/sqrt(tausq.inv) # SD in eq 2.3
  omega <- 1/sqrt(omsq.inv) # SD in eq 2.5
  alph[2] <- 0
  gam[2] <- 0
  
  for(i in 1:S){

    pB[i] <- ilogit(logitpB[i])
    pC[i] <- ilogit(logitpC[i])
    
    nuL[i] <- alph0 + alph[1] * x[i,1] + inprod(alph[3:5], x[i,3:5]) # mean in eq 2.3
    nuH[i] <- gam0 + gam[1] * x[i,1] + inprod(gam[3:5], x[i,3:5]) # mean in eq 2.5

    for(j in 1:n[i]){

      pH[i,j] <- z[i,j] * pC[i] # inside eq 3.1aa
    }
  }
  
  
  # ---------- likelihood
  
  for(i in 1:S){
    
    logitpB[i] ~ dnorm(nuL[i], tausq.inv) # eq 2.3
    logitpC[i] ~ dnorm(nuH[i], omsq.inv) # eq 2.5
    
    for(j in 1:n[i]){

      z[i,j] ~ dbern(pB[i]) # eq 2.2a 
      t[i,j] ~ dbern(pH[i,j]) # eq 3.1aa
    }    
  }
  
  
  # ---------- priors
  
  tausq.inv ~ dgamma(1, .01)
  omsq.inv ~ dgamma(1, .01)
  
  alph0 ~ dnorm(0, .001)
  gam0 ~ dnorm(0, .001)
  alph[1] ~ dnorm(0, .001)
  gam[1] ~ dnorm(0, .001)

  for(k in 3:5){
    
    alph[k] ~ dnorm(0, .001)
    gam[k] ~ dnorm(0, .001)
        
  }
}
"  
  fit.aa <- run.jags(jags.script.aa, data=dat.a, n.chains=2,
                      inits=list(tausq.inv=1, omsq.inv=1),
                      adapt=1000, burnin=10000, sample=5000, thin=10,
                      monitor=c(
                        "t[1,6]","t[1,9]","t[1,17]","t[1,22]","t[2,1]",
                        "t[2,8]","t[2,9]","t[2,13]","t[3,10]","t[3,12]",
                        "t[3,21]","t[4,1]","t[4,8]","t[4,13]","t[5,5]",
                        "t[5,8]","t[5,9]","t[5,16]","t[6,4]","t[6,8]",
                        "t[6,12]","t[6,16]","t[7,3]","t[7,10]","t[7,15]",
                        "t[8,6]","t[9,4]","t[10,32]","t[11,14]","t[12,2]",
                        "t[14,6]","t[14,11]","t[14,15]","t[14,17]","t[14,18]",
                        "t[16,9]","t[16,18]","t[16,22]","t[16,30]","t[16,36]",
                        "t[17,4]","t[17,12]","t[17,24]","t[18,11]","t[18,21]",
                        "t[18,26]","t[18,28]","t[18,29]","t[19,3]","t[20,11]",
                        "t[20,23]","t[21,18]","t[21,21]","t[21,26]","t[21,30]",
                        "t[21,32]","t[23,7]","t[24,3]","t[24,5]","t[24,6]",
                        "t[24,11]","t[24,15]","t[24,27]","t[24,35]","t[24,37]",
                        "t[24,39]","t[24,40]","t[25,12]","t[25,19]","t[25,25]",
                        "t[26,19]","t[26,30]","t[26,31]","t[27,27]","t[28,3]",
                        "t[28,20]","t[29,1]","t[30,10]",
                        "logitpB","logitpC","alph0","gam0","alph","gam","tau","omega","deviance","pd","dic"))  
  ## module dic loaded
## Compiling rjags model...
## Calling the simulation using the rjags method...
## Adapting the model for 1000 iterations...
##   |++++++++++++++++++++++++++++++++++++++++++++++++++| 100%
## Burning in the model for 10000 iterations...
##   |**************************************************| 100%
## Running the model for 50000 iterations...
##   |**************************************************| 100%
## Extending 50000 iterations for pD/DIC estimates...
##   |**************************************************| 100%
## Simulation complete
## Calculating summary statistics...
## Note: The monitored variables 'alph[2]' and 'gam[2]' appear to be non-stochastic;
## they will not be included in the convergence diagnostic
## Calculating the Gelman-Rubin statistic for 153 variables....
## Finished running the simulation
## Warning message:
## The length of the initial values argument supplied found does not correspond to the number of chains specified.  Some initial values were recycled or ignored.   
  scans.aa <- as.mcmc.list(fit.aa)
scans.aa.pooled <- rbind(scans.aa[[1]], scans.aa[[2]])  
  fit.aa$psrf$mpsrf # Gelman-Rubin convergence check  
  ## [1] 1.031637  
  fit.aa.dic.alt <- mean(scans.aa.pooled[,"deviance"]) + var(scans.aa.pooled[,"deviance"])/2
fit.aa.dic.alt  
  ## [1] 1190.508  
  par(mfrow=c(4,3))
traceplot(scans.aa)  
                           
 No apparent mixing issues, even for logit( \(p_i^c\) ) for  \(i\)  =
12 (ID=612), 14 (ID=614), 19 (ID=619), 30 (ID=630) where RLB tests gave either all NAs or most nonNAs that were 0. 
     
 
 Hypothetical model as if RLB test had been administered to entire
sample 
  \[  
\begin{aligned}  
\text{[2.2a]} &&  
\left. z_{ij} \right| p_i^B \sim \text{(ind) Bernoulli}(p_i^B) \\  
\text{[2.3]} &&  
\left. \text{logit } p_i^B \right| \alpha_0, \boldsymbol{\alpha}, \tau^2, \boldsymbol{x}_i \sim \text{(ind) } N(\alpha_0 + \boldsymbol{x}_i ' \boldsymbol{\alpha}, \tau^2) \\  
\text{[2.6]} &&  
\left. v_{ij} \right| \{z_{ij}=1\}, p_i^S \sim \text{(ind) Bernoulli}(p_i^S) \\  
\text{[3.1a]} &&
\left. t_{ij} \right| p_i^B, p_i^c \sim \text{(ind) Bernoulli}(p_i^B p_i^c) \\
\text{[2.7a]} &&  
p_i^S = \frac{1-p_i^c/p_i^{FH}}{1-p_i^{SH}/p_i^{FH}} \\
&& p_i^{SH} < p_i^c < p_i^{FH} \\
\text{[2.5]} &&  
\text{logit } p_i^c \left| \gamma_0, \boldsymbol{\gamma}, \omega^2, \boldsymbol{x}_i \right. \sim \text{(ind) } N(\gamma_0 + \boldsymbol{x}_i ' \boldsymbol{\gamma}, \omega^2) \\  
\end{aligned}  
\]  
  jags.script.cc <- "

model{ # hypothetical, RLB, alpha_2=gamma_2=0 (ignore emptyVariable)

  ########################### NOTE ##########################
  ##
  ## To reflect conditional binomials, partition n_i for
  ## each i into
  ##
  ##         n_i = n_i_noLyme + n_i_RLB + n_i_noRLB
  ##
  ## and similarly for the associated data vectors z_i, t_i, v_i.
  ## Thus, if the partition is irrelevant to the model statement
  ## (e.g. Eq. [3.1a]), then must use same distribution (with 
  ## same parameters) over all loop partitions under likelihood.
  ##
  ##############################################################
  
  # ---------- definitions
  
  tau <- 1/sqrt(tausq.inv) # SD in eq 2.3
  omega <- 1/sqrt(omsq.inv) # SD in eq 2.5
  alph[2] <- 0
  gam[2] <- 0
  
  for(i in 1:S){

    pB[i] <- ilogit(logitpB[i])
    pC[i] <- ilogit(logitpC[i])
    pS[i] <- (1-pC[i]/pFH[i])/(1-pSH[i]/pFH[i]) # eq 2.7
    logitpS[i] <- logit(pS[i])
    pH[i] <- pB[i] * pC[i] # inside eq. 3.1a
    logitpSH[i] <- logit(pSH[i])
    logitpFH[i] <- logit(pFH[i])

    nuL[i] <- alph0 + alph[1] * x[i,1] + inprod(alph[3:5], x[i,3:5]) # mean in eq 2.3
    nuH[i] <- gam0 + alph[1] * x[i,1] + inprod(gam[3:5], x[i,3:5]) # mean in eq 2.5     
  }
    
  # ---------- likelihood
  
  for(i in 1:S){
    
    logitpB[i] ~ dnorm(nuL[i], tausq.inv) # eq 2.3    
    logitpC[i] ~ dnorm(nuH[i], omsq.inv) # eq 2.5

    for(j in 1:n_noLyme[i]){ # z=0

      z_noLyme[i,j] ~ dbern(pB[i]) # eq 2.2 for PCR-
      t_noLyme[i,j] ~ dbern(pH[i]) # eq 3.1a for PCR-
    }
  }

  for(i in 1:12){ # z=1, v=0

    for(j in 1:n_noRLB[i]){
      z_noRLB[i,j] ~ dbern(pB[i]) # eq 2.2 for PCR+, v[i,j] = 0
      v_noRLB[i,j] ~ dbern(pS[i]) # eq 2.6 for PCR+, v[i,j] = 0
      t_noRLB[i,j] ~ dbern(pH[i]) # eq 3.1a for PCR+, v[i,j] = 0
    }
  }    

  for(i in 14:14){ # z=1, v=0

    for(j in 1:n_noRLB[i]){
      z_noRLB[i,j] ~ dbern(pB[i]) # eq 2.2 for PCR+, v[i,j] = 0
      v_noRLB[i,j] ~ dbern(pS[i]) # eq 2.6 for PCR+, v[i,j] = 0
      t_noRLB[i,j] ~ dbern(pH[i]) # eq 3.1a for PCR+, v[i,j] = 0
    }
  }    

  for(i in 16:21){ # z=1, v=0

    for(j in 1:n_noRLB[i]){
      z_noRLB[i,j] ~ dbern(pB[i]) # eq 2.2 for PCR+, v[i,j] = 0
      v_noRLB[i,j] ~ dbern(pS[i]) # eq 2.6 for PCR+, v[i,j] = 0
      t_noRLB[i,j] ~ dbern(pH[i]) # eq 3.1a for PCR+, v[i,j] = 0
    }
  }    

  for(i in 23:S){ # z=1, v=0

    for(j in 1:n_noRLB[i]){
      z_noRLB[i,j] ~ dbern(pB[i]) # eq 2.2 for PCR+, v[i,j] = 0
      v_noRLB[i,j] ~ dbern(pS[i]) # eq 2.6 for PCR+, v[i,j] = 0
      t_noRLB[i,j] ~ dbern(pFH[i]) # eq 3.1a for PCR+, v[i,j] = 0
    }
  }    

  for(i in 1:11){ # z=v=1

    for(j in 1:n_RLB[i]){

        z_RLB[i,j] ~ dbern(pB[i]) # eq 2.2 for PCR+, v[i,j] = 1
        v_RLB[i,j] ~ dbern(pS[i]) # eq 2.6 for PCR+, v[i,j] = 1
        t_RLB[i,j] ~ dbern(pSH[i]) # eq 3.1a for PCR+, v[i,j] = 1
    }    
  }

  for(i in 13:18){ # z=v=1

    for(j in 1:n_RLB[i]){

        z_RLB[i,j] ~ dbern(pB[i]) # eq 2.2 for PCR+, v[i,j] = 1
        v_RLB[i,j] ~ dbern(pS[i]) # eq 2.6 for PCR+, v[i,j] = 1
        t_RLB[i,j] ~ dbern(pSH[i]) # eq 3.1a for PCR+, v[i,j] = 1
    }    
  }

  for(i in 20:S){ # z=v=1

    for(j in 1:n_RLB[i]){

        z_RLB[i,j] ~ dbern(pB[i]) # eq 2.2 for PCR+, v[i,j] = 1
        v_RLB[i,j] ~ dbern(pS[i]) # eq 2.6 for PCR+, v[i,j] = 1
        t_RLB[i,j] ~ dbern(pSH[i]) # eq 3.1a for PCR+, v[i,j] = 1
    }    
  }

  # ---------- priors
  
  tausq.inv ~ dgamma(1, .01)
  omsq.inv ~ dgamma(1, .01)
  
  alph0 ~ dnorm(0, .001)
  gam0 ~ dnorm(0, .001)
  alph[1] ~ dnorm(0, .001)
  gam[1] ~ dnorm(0, .001)

  for(k in 3:5){
    
    alph[k] ~ dnorm(0, .001)
    gam[k] ~ dnorm(0, .001)
        
  }

  for(i in 1:S){

    pSH[i] ~ dunif(0, pC[i])
    pFH[i] ~ dunif(pC[i], 1)
  }
}
"  
  fit.cc <- run.jags(jags.script.cc, data=dat.c, n.chains=2,
                      inits=list(tausq.inv=1, omsq.inv=1),
                      adapt=10000, burnin=10000, sample=5000, thin=10,
                      monitor=c(
                        "t_noRLB[1,1]","t_noRLB[1,2]","t_noRLB[1,3]","t_noRLB[1,4]",
                        "t_noRLB[2,1]","t_noRLB[2,2]","t_noRLB[2,3]","t_noRLB[2,4]",
                        "t_noRLB[3,1]","t_noRLB[3,2]","t_noRLB[3,3]",
                        "t_noRLB[4,1]","t_noRLB[4,2]","t_noRLB[4,3]",
                        "t_noRLB[5,1]","t_noRLB[5,2]","t_noRLB[5,3]","t_noRLB[5,4]",
                        "t_noRLB[6,1]","t_noRLB[6,2]","t_noRLB[6,3]","t_noRLB[6,4]",
                        "t_noRLB[7,1]","t_noRLB[7,2]","t_noRLB[7,3]",
                        "t_noRLB[8,1]",
                        "t_noRLB[9,1]",
                        "t_noRLB[10,1]",
                        "t_noRLB[11,1]",
                        "t_noRLB[12,1]",
                        "t_noRLB[14,1]","t_noRLB[14,2]","t_noRLB[14,3]","t_noRLB[14,4]","t_noRLB[14,5]",
                        "t_noRLB[16,1]","t_noRLB[16,2]","t_noRLB[16,3]","t_noRLB[16,4]","t_noRLB[16,5]",
                        "t_noRLB[17,1]","t_noRLB[17,2]","t_noRLB[17,3]",
                        "t_noRLB[18,1]","t_noRLB[18,2]","t_noRLB[18,3]","t_noRLB[18,4]","t_noRLB[18,5]",
                        "t_noRLB[19,1]",
                        "t_noRLB[20,1]","t_noRLB[20,2]",
                        "t_noRLB[21,1]","t_noRLB[21,2]","t_noRLB[21,3]","t_noRLB[21,4]","t_noRLB[21,5]",
                        "t_noRLB[23,1]",
                        "t_noRLB[24,1]","t_noRLB[24,2]","t_noRLB[24,3]","t_noRLB[24,4]","t_noRLB[24,5]",
                        "t_noRLB[24,6]","t_noRLB[24,7]","t_noRLB[24,8]","t_noRLB[24,9]","t_noRLB[24,10]",
                        "t_noRLB[25,1]","t_noRLB[25,2]","t_noRLB[25,3]",
                        "t_noRLB[26,1]","t_noRLB[26,2]","t_noRLB[26,3]",
                        "t_noRLB[27,1]",
                        "t_noRLB[28,1]","t_noRLB[28,2]",
                        "t_noRLB[29,1]",
                        "t_noRLB[30,1]",
                        "logitpB","logitpC","logitpS","logitpSH","logitpFH",
                        "alph0","alph","gam0","gam","tau","omega","deviance","pd","dic"))  
  ## module dic loaded
## Compiling rjags model...
## Calling the simulation using the rjags method...
## Adapting the model for 10000 iterations...
##   |++++++++++++++++++++++++++++++++++++++++++++++++++| 100%
## Burning in the model for 10000 iterations...
##   |**************************************************| 100%
## Running the model for 50000 iterations...
##   |**************************************************| 100%
## Extending 50000 iterations for pD/DIC estimates...
##   |**************************************************| 100%
## Simulation complete
## Calculating summary statistics...
## Note: The monitored variables 'alph[2]' and 'gam[2]' appear to be non-stochastic; they will not be
## included in the convergence diagnostic
## Calculating the Gelman-Rubin statistic for 243 variables....
## Finished running the simulation
## Warning messages:
## 1: The length of the initial values argument supplied found does not correspond to the number of chains specified.  Some initial values were recycled or ignored. 
## 2: In rjags::jags.samples(rjags, variable.names = monitor, n.iter = extra.options$sample,  :
##   Failed to set trace monitor for pD
## pD is infinite because at least one observed node does not have fixed support  
  scans.cc <- as.mcmc.list(fit.cc)
scans.cc.pooled <- rbind(scans.cc[[1]], scans.cc[[2]])  
  fit.cc$psrf$mpsrf # Gelman-Rubin convergence check  
  ## [1] 1.032797  
  fit.cc.dic.alt <- mean(scans.cc.pooled[,"deviance"]) + var(scans.cc.pooled[,"deviance"])/2
fit.cc.dic.alt  
  ## [1] 1660.277  
  par(mfrow=c(4,3))
traceplot(scans.cc)  
                                           
     
 
 
 Diagnosis for realistic model with RLB 
 Now we diagnose the “residuals”  \(\eta\)  and  \(\xi\)  (note that normality of posterior of each  \(\eta_i\)  and  \(\xi_i\)  is irrelevant): 
  eta <- scans.c.pooled[,79:108] # just logit(pB)
eta <- eta - scans.c.pooled[,"alph0"] # logit(pB) - alph0
eta <- eta - scans.c.pooled[,c(230,232:234)] %*% t(dat$x[,c(1,3:5)]) # actual eta

xi <- scans.c.pooled[,109:138] # just logit(pC)
xi <- xi - scans.c.pooled[,"gam0"] # logit(pC) - gam0
xi <- xi - scans.c.pooled[,c(236,238:240)] %*% t(dat$x[,c(1,3:5)]) # actual xi  
  par(mfrow=c(4,1))
par(cex.lab=1.5)
par(cex.main=2)

zoomlim <- 2.5
densfac <- 4

covar <- colnames(dat$x)[c(1,3:5)]
dens <- apply(eta, 2, density) 

dens.x <- NULL
dens.y <- NULL

for(i in 1:nrow(dat$x)){
  
  dens.x <- cbind(dens.x, dens[[i]]$x)
  dens.y <- cbind(dens.y, dens[[i]]$y)
}

for(k in covar){
  
  plot(1:2, type="n", xlim=range(dat$x[,k]), ylim=range(dens.x),
    xlab=paste("2006",k), ylab="eta posterior") # dummy plot
  abline(h=0)
  
  for(i in 1:nrow(dat$x))
    points(rep(dat$x[i,k],length(dens.x[,i])), dens.x[,i], cex=dens.y[,i]*densfac)
}

tmp <- rbind( c(-.4,.8),
              c(-.6,.6),
              c(-1,2.1),
              c(-.8,1.8)
              )

rownames(tmp) <- covar

for(k in covar){
  
  plot(1:2, type="n", ylim=c(-zoomlim,zoomlim),
    xlim=tmp[k,], main="omit influential resid, zoomed in",   
    xlab=paste("2006",k), ylab="eta posterior") # dummy plot
  abline(h=0)
  
  for(i in 1:nrow(dat$x))
    points(rep(dat$x[i,k],length(dens.x[,i])), dens.x[,i], cex=dens.y[,i]*densfac)
}

plot(1:nrow(dat$x), type="n", ylim=range(dens.x),
  xlab="2006 site i", ylab="eta posterior") # dummy plot
abline(h=0)
  
for(i in 1:nrow(dat$x))
  points(rep(i,length(dens.x[,i])), dens.x[,i], cex=dens.y[,i]*3)

plot(1:nrow(dat$x), type="n", ylim=c(-zoomlim,zoomlim),
  xlab="2006 site i", ylab="eta posterior", main="zoomed in") # dummy plot
abline(h=0)
  
for(i in 1:nrow(dat$x))
  points(rep(i,length(dens.x[,i])), dens.x[,i], cex=dens.y[,i]*3)  
       
  par(mfrow=c(4,1))
par(cex.lab=1.5)
par(cex.main=2)

zoomlim <- .6
densfac <- 1.5

covar <- colnames(dat$x)[c(1,3:5)]
dens <- apply(xi, 2, density) 

dens.x <- NULL
dens.y <- NULL

for(i in 1:nrow(dat$x)){
  
  dens.x <- cbind(dens.x, dens[[i]]$x)
  dens.y <- cbind(dens.y, dens[[i]]$y)
}

tmp <- rbind( c(-.4,.8),
              c(-.6,.6),
              c(-1,2.1),
              c(-.8,1.8)
              )

rownames(tmp) <- covar


for(k in covar){
  
  plot(1:2, type="n", xlim=range(dat$x[,k]), ylim=range(dens.x),
    xlab=paste("2006",k), ylab="xi posterior") # dummy plot
  abline(h=0)
  
  for(i in 1:nrow(dat$x))
    points(rep(dat$x[i,k],length(dens.x[,i])), dens.x[,i], cex=dens.y[,i]*densfac)
}

for(k in covar){
  
  plot(1:2, type="n", ylim=c(-zoomlim,zoomlim),
    xlim=tmp[k,], main="omit influential resid, zoomed in",   
    xlab=paste("2006",k), ylab="xi posterior") # dummy plot
  abline(h=0)
  
  for(i in 1:nrow(dat$x))
    points(rep(dat$x[i,k],length(dens.x[,i])), dens.x[,i], cex=dens.y[,i]*densfac)
}

plot(1:nrow(dat$x), type="n", ylim=range(dens.x),
  xlab="2006 site i", ylab="xi posterior") # dummy plot
abline(h=0)
  
for(i in 1:nrow(dat$x))
  points(rep(i,length(dens.x[,i])), dens.x[,i], cex=dens.y[,i]*densfac)

plot(1:nrow(dat$x), type="n", ylim=c(-zoomlim,zoomlim),
  xlab="2006 site i", ylab="xi posterior", main="zoomed in") # dummy plot
abline(h=0)
  
for(i in 1:nrow(dat$x))
  points(rep(i,length(dens.x[,i])), dens.x[,i], cex=dens.y[,i]*densfac)  
       
 The full model  fit.c  shows no mixing issues nor undesirable residual patterns => can use these results as final inference. 
    
 
 
 
 Compare fit (posterior SD) and goodness-of-fit (default DIC) 
 
 
  
  
  
  
  
  
  
 
 
 
 model 
 z 
 t 
 RLB 
 realistic? 
 reduced? 
 DIC 
 
 
 
 
 aa 
 Y 
 Y 
 N 
 Y (except for ignoring RLB failure) 
 Y 
 1191 
 
 
 c 
 Y 
 Y 
 Y 
 Y 
 N 
 1466 
 
 
 cc 
  Y 
 Y 
 Y 
 N 
 N 
 1623 
 
 
 
 NOTE: DIC is based on deviance => can only compare DICs among models that consider the same set of responses. 
  # names(fit.aa$summary$stat[,"SD"])
# rbind(names(fit.c$summary$stat[,"SD"]),names(fit.cc$summary$stat[,"SD"]))

pBpCcolname <- names(fit.aa$summary$stat[,"SD"])[79:138]
coef_prec_colname <- names(fit.aa$summary$stat[,"SD"])[c(139:141,143:146,148:153)]
paramcolname <- names(fit.aa$summary$stat[,"SD"])[c(139:141,143:146,148:153,79:138)]

library(boot)
merge(
  t(data.frame(c(mod="aa",inv.logit(fit.aa$summary$quant[pBpCcolname,"50%"])))),
  merge(t(data.frame(c(mod="c",inv.logit(fit.c$summary$quant[pBpCcolname,"50%"])))),
		t(data.frame(c(mod="cc",inv.logit(fit.cc$summary$quant[pBpCcolname,"50%"])))),all=T),
  all=T) # posterior medians on original scale    
  ##      mod        logitpB[1]        logitpB[2]        logitpB[3]
## 1     aa 0.286275680341725 0.377200798547525 0.312362346164428
## 2      c 0.287744327216969 0.378251170025369 0.314126431482953
## 3     cc 0.224213947625426 0.307636152454255 0.256245009761382
##          logitpB[4]        logitpB[5]        logitpB[6] logitpB[7]
## 1 0.333320129802471 0.333546224376753 0.404328899350201 0.358182213551589
## 2  0.33273271444382 0.335304338494897 0.405307694333071 0.357910001271798
## 3 0.252931498295195 0.292291375968954 0.345234553420738 0.290652223313192
##          logitpB[8]        logitpB[9]       logitpB[10] logitpB[11]
## 1 0.212150507330821 0.404272020611921 0.409252881052269 0.390894096861515
## 2 0.209849124469809 0.402433816764527 0.410004416304936 0.389385091694235
## 3 0.149586526014099 0.331262650409587  0.35675183027141 0.340923617350502
##         logitpB[12]       logitpB[13]       logitpB[14] logitpB[15]
## 1 0.335313072059939 0.348905889180321 0.434472747369546 0.251674227818513
## 2 0.334363696729813 0.348753517137964 0.436121365539287 0.249206735297587
## 3 0.275679026033247 0.270449305788599 0.387066324241151 0.178041036487967
##         logitpB[16]       logitpB[17]       logitpB[18] logitpB[19]
## 1  0.42955600748724 0.366330141310985 0.400648030661053 0.191891032235729
## 2  0.42953950755372 0.367430551944616 0.400119611856332 0.189647923512377
## 3 0.378756111704248 0.306012892909537 0.326235417062445 0.153553361498798
##         logitpB[20]       logitpB[21]       logitpB[22] logitpB[23]
## 1 0.326363546450276 0.252764360230601 0.261232120759884 0.358721044777837
## 2 0.325046572032911 0.252004501866955 0.260978001564976 0.359017621768765
## 3 0.255709291238385 0.182133079083367 0.198205735555762 0.273760014552085
##         logitpB[24]       logitpB[25]       logitpB[26] logitpB[27]
## 1 0.451536382056986 0.243381260659997 0.270201618433772 0.332412097240632
## 2 0.454896083121185 0.241001728322721 0.269694471557962 0.330568945665612
## 3 0.404354554762651 0.175073322027557 0.245871971801113 0.251673468560199
##          logitpB[28]       logitpB[29]       logitpB[30] logitpC[1]
## 1  0.123071300629036 0.318363385503664 0.335355588162847 0.745288558734118
## 2  0.122653217680628  0.31785890240741 0.333356098851662 0.74388082083711
## 3 0.0830672822253752 0.245608286904683 0.234302833709962 0.559651674957678
##          logitpC[2]        logitpC[3]        logitpC[4] logitpC[5]
## 1 0.626628558440849 0.597238496585605  0.57131403341174 0.472648042910999
## 2 0.652178303522756 0.642987368466495 0.625826888457087 0.570161824386182
## 3 0.494951590809613 0.453976445580962 0.472258280285624 0.374390546012562
##          logitpC[6]        logitpC[7]        logitpC[8] logitpC[9]
## 1 0.351022157092187 0.701857163421253 0.595649608340639 0.584528800489353
## 2  0.49331412832672 0.692932518824053 0.643607335243804 0.620779773105128
## 3 0.364065428879117 0.515663992989087  0.44031440266289 0.479608686889698
##         logitpC[10]       logitpC[11]       logitpC[12] logitpC[13]
## 1 0.619981884519604 0.387082348967783  0.43203406833043 0.645979711554642
## 2 0.634799850487406  0.50034685668626 0.542793211748421 0.664176002906938
## 3 0.455033795838808 0.345152614333685  0.38642975154125 0.515231754948423
##         logitpC[14]       logitpC[15]       logitpC[16] logitpC[17]
## 1 0.331262483904011 0.532384373076934 0.518854031589226 0.597483589768923
## 2 0.465570988022531 0.588687159748592 0.577234469934066 0.622682898118569
## 3  0.33732555449222 0.419110254564096 0.412985899846002 0.43555757236538
##         logitpC[18]       logitpC[19]       logitpC[20] logitpC[21]
## 1  0.60319303785903 0.200119506928761 0.609532090156252 0.576609262170153
## 2 0.618993706294134 0.393220113339315 0.628411363018045 0.646718539820754
## 3 0.461571985056062 0.226340328894845 0.447520960831188 0.482882534579423
##         logitpC[22]       logitpC[23]       logitpC[24] logitpC[25]
## 1 0.637273827204701 0.704482774696421  0.64104203738729 0.634928209434662
## 2 0.658876245770762 0.701785964702165 0.662655482862873 0.658035822970187
## 3 0.462680358109986  0.55936643975727 0.485360682165771 0.456002959681775
##         logitpC[26]       logitpC[27]       logitpC[28] logitpC[29]
## 1 0.164209456005324 0.453401538519058 0.534496449198115 0.541704043509629
## 2 0.355983607150089 0.540992886488328 0.683607311790795 0.60067451775838
## 3 0.199624866560396 0.407026081407832 0.496699927115124 0.427557274251797
##         logitpC[30]
## 1 0.658603558781841
## 2   0.6631499792292
## 3 0.527277910527855  
  merge(
  t(data.frame(c(mod="aa",fit.aaNoS$summary$quant[coef_prec_colname,"50%"]))),
  merge(t(data.frame(c(mod="c",fit.c$summary$quant[coef_prec_colname,"50%"]))),
      t(data.frame(c(mod="cc",fit.cc$summary$quant[coef_prec_colname,"50%"]))),all=T),
  all=T) # posterior medians  
  ##      mod              alph0             alph[1] alph[3]
## 1     aa -0.744991260848557  -0.037064029359063 0.526552167166945
## 2      c -0.746272681636666 -0.0447808198563683 0.524654133413428
## 3     cc  -1.06436348483086   0.056222679861029 0.612238438073267
##              alph[4]            alph[5]               gam0
## 1 0.0990121670573621  0.127944746116117  0.159750354003106
## 2 0.0994082840930224  0.129944965409824  0.427962474216662
## 3 0.0972637241323568 0.0909995174312724 -0.263709832052086
##               gam[1]             gam[3]              gam[4]
## 1   0.40271445400255 -0.964487762106847  -0.075542216120264
## 2  0.200476322719819 -0.727196202093208   -0.06117827688635
## 3 -0.126804139800104 -0.596788498112673 -0.0114243798173063
##              gam[5]               tau             omega deviance
## 1 0.476582194793155 0.380267456196503 0.120862543981439 1128.28973675357
## 2 0.256562292312631  0.38582967125466 0.111443679616285 1419.30066145254
## 3 0.275272719667932 0.444427261166902 0.112951969251933 1575.88401583129  
  merge(
  t(data.frame(c(mod="aa",fit.aaNoS$summary$stat[paramcolname,"SD"]))),
    merge(t(data.frame(c(mod="c",fit.c$summary$stat[paramcolname,"SD"]))),
      t(data.frame(c(mod="cc",fit.cc$summary$stat[paramcolname,"SD"]))),all=T),
  all=T) # posterior SDs (logit scale for p)  
  ##      mod             alph0           alph[1] alph[3]
## 1     aa 0.116453901998254 0.244497922882195 0.292844147177524
## 2      c 0.116234075166985 0.247367349146739 0.299950929706252
## 3     cc  0.12612288369119 0.180670977643597 0.320528626585234
##              alph[4]           alph[5]              gam0 gam[1]
## 1 0.0809370352946895 0.105976347573409 0.187544594176602 0.357759912569506
## 2 0.0823956227297001  0.10598949954466 0.152483952758831 0.310660053295692
## 3 0.0855679511750812   0.1153994651087 0.158217678502502 31.7869838247839
##              gam[3]            gam[4]            gam[5] tau
## 1 0.543777405365543 0.144392324232291  0.21224718903663 0.154169351522706
## 2 0.420954839735748 0.118711063852355 0.156644397871067 0.153569925700505
## 3 0.430344819566998 0.117445416698138 0.164123373657617 0.142929070984102
##                omega         deviance        logitpB[1] logitpB[2]
## 1  0.115232543966504 11.0503189324571  0.36808507794432 0.330575156724792
## 2 0.0795333626354588 13.4941555969172 0.374554139418522 0.33039448070943
## 3 0.0785776236636045 12.9210558854353 0.378324097879355 0.353445382380385
##          logitpB[3]        logitpB[4]        logitpB[5] logitpB[6]
## 1 0.256970715815465 0.340148497841952 0.347524194992219 0.385138520035015
## 2  0.25635580013168 0.345754568303797 0.356625171203561 0.386074950180639
## 3 0.268091262086179 0.362519201759414 0.373006655266224 0.36791872930912
##          logitpB[7]        logitpB[8]        logitpB[9] logitpB[10]
## 1  0.26779456435986  0.36405441589406 0.382725347054261 0.278744229790735
## 2 0.269921003649936 0.365808055108282 0.381791845827488 0.280702650581445
## 3  0.27653220675284 0.378743689170874 0.412093591241131 0.289335660022653
##         logitpB[11]       logitpB[12]       logitpB[13] logitpB[14]
## 1  0.32810386565998 0.421507625290027 0.364966834101772 0.331562473142562
## 2 0.335996233153091 0.425613414082882 0.361599609696398 0.327252531923079
## 3 0.353704085215468 0.472024328314693 0.381715583170823 0.341323400458301
##         logitpB[15]       logitpB[16]       logitpB[17] logitpB[18]
## 1 0.365112755140872 0.261514128392857 0.268227851056739 0.247752388273688
## 2 0.367125280541311 0.261254561431267 0.270708208583391 0.243397566333423
## 3  0.37257259938427 0.268633047657384 0.279394059100425 0.252300246372432
##         logitpB[19]       logitpB[20]       logitpB[21] logitpB[22]
## 1 0.478385544590157 0.287757608347857 0.303472381427287 0.361929729820525
## 2 0.495000252307315 0.288818114726033 0.308889287305192 0.370991578715777
## 3 0.496705227799186 0.296412686414443 0.323209468904882 0.390507431316728
##         logitpB[23]       logitpB[24]       logitpB[25] logitpB[26]
## 1 0.375520511981175 0.305375533751416 0.372589726571813 0.334870839638384
## 2 0.384299240968608 0.303384279710179 0.374632246712722 0.331759022978076
## 3  0.40734517039777 0.290919004909208  0.38665726537675 0.347122441790222
##         logitpB[27]       logitpB[28]       logitpB[29] logitpB[30]
## 1 0.326059674728195 0.510438702973802 0.382488800297149 0.386179255525152
## 2 0.329575159442413 0.514251361870474  0.38342310784036 0.388579425123633
## 3 0.333244309064732 0.527682586757859 0.414233137813887 0.404113344620195
##          logitpC[1]        logitpC[2]        logitpC[3] logitpC[4]
## 1  0.65718772396178 0.319481171724085 0.363891573678911 0.349091954389417
## 2 0.509771983403403 0.257627393401854 0.306107880675853 0.284672599232602
## 3 0.537904822600131  0.25580059921283 0.298916414586777 0.254900518085732
##          logitpC[5]        logitpC[6]        logitpC[7] logitpC[8]
## 1 0.437459647852321 0.592349749243151 0.322520390645636 0.479528853242403
## 2 0.371514591861682 0.479221083279775 0.279690395052763 0.391229757671555
## 3 0.359832619973532 0.420877360250204 0.257948142837326 0.393867622480815
##          logitpC[9]       logitpC[10]       logitpC[11] logitpC[12]
## 1  0.39199819707069 0.281501647392307 0.331906596410157 0.380823587579195
## 2 0.331201230928907 0.243507740181076 0.265493356341518 0.288193988646584
## 3 0.307430388751561 0.218073470999664 0.264784335866168 0.289007304268889
##         logitpC[13]       logitpC[14]       logitpC[15] logitpC[16]
## 1 0.378452739759891 0.510901099136485 0.255151536444294 0.28597753782729
## 2 0.317834397386474 0.376367627545367 0.211954261059861 0.2363951486285
## 3 0.295927607369386 0.376989093886644 0.212600634245666 0.241710186300164
##         logitpC[17]       logitpC[18]       logitpC[19] logitpC[20]
## 1 0.306316390968879 0.308997360640142 0.769368219658805 0.298262539199432
## 2 0.270766595207796  0.25610339327358 0.572462522295954 0.246742752175197
## 3 0.228942140094307 0.267565882313455 0.590913957442594 0.231629700286442
##         logitpC[21]       logitpC[22]       logitpC[23] logitpC[24]
## 1  0.43874009995545 0.353826454496628 0.456091014347591 0.256636873053592
## 2 0.351421886786474 0.308140633446226 0.377791457732199 0.221833981760168
## 3 0.327152649754851 0.271588976331774 0.346740220122096 0.216316922335039
##         logitpC[25]       logitpC[26]       logitpC[27] logitpC[28]
## 1 0.508223038794655 0.746674255890884 0.421884219640203 1.21616410728608
## 2 0.420807453238395  0.57298422080111 0.347338963174625 0.946725155311658
## 3 0.404313406457059 0.589574894313576 0.311446286722094 0.924409310399316
##         logitpC[29]       logitpC[30]
## 1 0.470943016358126 0.469062695761407
## 2 0.380520353772254   0.3891048479601
## 3  0.38986686966711  0.35670145197181
  
 
 Posterior median (original scale) and SD (logit scale) of each  \(p^B\)  are mostly comparable among models, similarly for median and SD of each  \(\alpha\)  and  \(\tau\) 
 
 to be expected because  \(\boldsymbol{z}\)  fully observed and modeled identically in all cases. 
  
 RLB models ( c  and  cc )
 
 have noticeably smaller posterior SD for each logit( \(p^c\) ), relevant  \(\gamma\) , and  \(\omega\)  => modeling  \(\boldsymbol{v}\)  improves HIS-related inference 
 
 additionally gain inference for  \(p^S, p^{SH}, p^{FH}\)  
  
 Unrealistic model ( cc )
 
 doesn’t reflect actual lab procedure (RLB tested only when Lyme detected) 
 
 poorer predictive performance (larger DICs) 
 
 posterior median for  \(\gamma_0\)  and some  \(p^c\)  differ noticeably 
 
 posterior SD for some  \(\gamma\)  and some logit( \(p^c\) ) differ noticeably 
  
 
   
   
 
 
 Some useful info for rest of document: 
  names(site)  
  ##  [1] "siteName"             "emptyVar"             "siteFragHa"          
##  [4] "siteFragCat"          "siteDenDragLen"       "siteDenNymphsDragged"
##  [7] "siteDON"              "siteLabNymphs"        "siteLabNIP"          
## [10] "siteDON.NIP"          "siteOspCdiv"          "siteHISProp"         
## [13] "hostHindexAll"        "siteRich"             "hostPELEprop"        
## [16] "hostTASTprop"         "hostBLBRprop"         "hostPropPTBtot"      
## [19] "sitePELEden"          "siteTASTden"          "siteBLBRden"         
## [22] "Level"  
 For each site, 
 
  \(p_B\)  = true rate of “Lyme+” (model parameter) 
 
  \(\lambda\)  = true spatial density of ticks (parameter, unmodeled) 
 
  \(\lambda p_B\)  = true rate of “Lyme+” per unit space (parameter, partially modeled) 
 
  \(n\)  =  siteLabNymphs  = # test ticks (by design) 
 
  \(y\)  = # observed “Lyme+” test ticks (random) 
 
  \(\hat{p}_B = y/n\)  =  siteLabNIP  = proportion of observed Lyme+ (random) 
 
  \(a\)  =  siteDenDragLen  = drag area (by design) 
 
  \(m\)  =  siteDenNymphsDragged  = # ticks observed in  \(a\)  (random) 
 
  \(\hat{\lambda} = m/a\)  =  siteDON  = observed spatial density of ticks (random) 
 
  \(\hat{\lambda}\hat{p}_B\)  =  site’s DIN = naive estimate  of “Lyme+” rate per unit space (random) 
=  siteDON.NIP  =  siteDON   \(\times\)   siteLabNIP  
 
   
 
 
 Naive NIP.All estimates and their modeled counterparts 
  tmp <- data.frame( site[,c("siteLabNIP","siteLabNymphs")] )

pB.naive.ci95 <- 1.96*sqrt(tmp[,1]*(1-tmp[,1])/tmp[,2]) # half width only
pB.naive.ci95 <- data.frame(lo=tmp[,1]-pB.naive.ci95, hi=tmp[,1]+pB.naive.ci95)
pB.c.ci95 <- apply(pB.c, 2, quantile, prob=c(.025,.5,.975))

tmp <- data.frame( tmp, t(pB.c.ci95), pB.naive.ci95 )
tmp <- tmp[ order(tmp[,1], decreasing = TRUE), ]
tmp1 <- rownames(tmp)

par(las=2) # make label text perpendicular to axis
par(mar=c(9,5,5,2)) # increase x-axis margin
par(mgp=c(3,.4,0))
tmp2 <- barplot( tmp[,1], 
                 names.arg = paste(tmp1, "(", 
                                   format(tmp[,2], justify="right"),
                                   ")"),
                 col="gray95", ylab=expression(paste(p[B], " = NIP")), 
                 ylim=c(-0.1,1) )
title("GRAY: naive NIP[All] estimate in desc. order and\nnaive 95% CI\nBLACK: modeled posterior median ('o') and\n95% credible interval")
text(tmp2[15], -.08, "site name (n)")

points(tmp2-.3, tmp[,6], pch="-", font=2, col="gray60")
points(tmp2-.3, tmp[,7], pch="-", font=2, col="gray60")

segments(tmp2-.3, tmp[,6], tmp2-.3, tmp[,7], lwd=2, col="gray60")

points(tmp2+.3, tmp[,4], pch="o", font=2)
points(tmp2+.3, tmp[,3], pch="-", font=2)
points(tmp2+.3, tmp[,5], pch="-", font=2)

segments(tmp2+.3, tmp[,3], tmp2+.3, tmp[,5], lwd=2)  
   
   
 
 
 Naive NIP[HIS] estimates and their modeled counterparts 
  library(boot)

pC.c <- inv.logit(scans.c.pooled[,109:138])  
pC.c.ci95 <- apply(pC.c, 2, quantile, prob=c(.025,.5,.975))
colnames(pC.c.ci95) <- site[,"siteName"]

pC.naive.ci95 <- sapply(site[,"siteName"],
              function(x){
                sum(subset(tick1.lyme, siteName==x)[,"his"])
              }) # HIS+ only

pC.naive.ci95 <- pC.naive.ci95 / tick2[,"lyme"] # naive pC est only

tmp <- data.frame(est=pC.naive.ci95, nh=tick2[,"lyme", drop=FALSE])
tmp  
  ##                      est lyme
##      601              NA    8
##      602              NA    6
##      603              NA   15
##      604              NA    4
##      605              NA    7
##      606              NA   11
##      607              NA   14
##      608              NA    4
##      609              NA    4
##      610              NA   15
##      611              NA    8
##      612              NA    1
##      613       0.7500000    4
##      614              NA   11
##      615       0.7500000    4
##      616              NA   19
##      617              NA   13
##      618              NA   18
##      619              NA    1
##      620              NA    9
##      621              NA    7
##      622       0.3333333    3
##      623              NA    4
##      624              NA   23
##      625              NA    4
##      626              NA   11
##      627              NA    7
##      628              NA    4
##      629              NA    3
##      630              NA    3  
  pC.naive.ci95 <- 1.96*sqrt(tmp[,1]*(1-tmp[,1])/tmp[,2]) # half width only
pC.naive.ci95 <- data.frame(est=tmp[,1], lo=tmp[,1]-pC.naive.ci95, hi=tmp[,1]+pC.naive.ci95)
rownames(pC.naive.ci95) <- rownames(tmp)

tmp <- data.frame( tmp, t(pC.c.ci95), pC.naive.ci95[,2:3] )
tmp <- tmp[ tmp1, ]

par(las=2) # make label text perpendicular to axis
par(mar=c(9,5,5,2)) # increase x-axis margin
par(mgp=c(3,.4,0))
tmp2 <- barplot( tmp[,1], 
                 names.arg = paste(tmp1, "(", 
                                   format(tmp[,2], justify="right"),
                                   ")"),
                 col="gray95", ylab=expression(paste(p[C], " = NIP[HIS]")), 
                 ylim=c(-0.1,1) )
title("GRAY: naive NIP[HIS] estimate\nBLACK: modeled posterior median ('o') and\n95% credible interval")
text(tmp2[15], -.04, "site name (y)")

points(tmp2, tmp[,4], pch="o", font=2)
points(tmp2, tmp[,3], pch="-", font=2)
points(tmp2, tmp[,5], pch="-", font=2)

segments(tmp2, tmp[,3], tmp2, tmp[,5], lwd=2)  
   
   
 
 
 Naive DIN estimates and their modeled (posterior predictive) counterparts 
For each site:
 
 CAUTION:
 
 posterior of  \(\hat{\lambda}p_B\)  isn’t a posterior predictive! 
 
 pointless to model  \(m, \lambda\)  because
 
 just one data point for  \(m\) , 
 
 no spatial info about sites so can’t model  \(\lambda\)  spatially 
  
  
 let  \(y^\ast\)  = # dragged ticks that would’ve been “Lyme+” (random, unobserved)
 
  \(E(y^\ast \mid m) = mp_B\)  
 
 get conditional posterior predictive for  \(y^\ast\) :
 
 let  \(p_B^{(s)}\)  =  \(s\) th MCMC scan of  \(p_B\)  
 
 simulate  \(y^\ast\mid m\sim\)  Bin( \(m,p_B^{(s)}\) ) 
 
  
 conditional posterior predictive for  siteDON.NIP  is that for  \(y^\ast/a\)  
 
 can’t use for cross validation because don’t have an observed version of  \(y^\ast\)  
  
 
  library(boot)

pB.c <- inv.logit(scans.c.pooled[,79:108])  
pB.c.ls <- as.list( as.data.frame(pB.c) )

library(parallel)

ystar.c <- mcmapply( rbinom, 
          site$siteDenNymphsDragged, 
          pB.c.ls, 
          MoreArgs = list(n=length(pB.c.ls)),
          mc.cores=6)

din.c <- t( t(ystar.c)/site$siteDenDragLen )
colnames(din.c) <- site$siteName
din.c.ci95 <- apply(din.c, 2, quantile, prob=c(.025,.5,.975))  
  tmp <- site[,c("siteDON.NIP","siteDenNymphsDragged")]
tmp <- tmp[ tmp1, ]

par(las=2) # make label text perpendicular to axis
par(mar=c(9,5,4,2)) # increase x-axis margin
par(mgp=c(3,.4,0))
tmp2 <- barplot( tmp[,1], 
                 names.arg = paste(tmp1, "(", 
                                   format(tmp[,2], justify="right"),
                                   ")"),
                 col="gray95", ylab=expression(paste(p[B]*m/a, " = DIN")), 
                 ylim=c(-0.015,.15) )
title("GRAY: naive DIN estimate\nBLACK: modeled posterior median ('o') and\n95% 'predictive' interval")
text(tmp2[15], -.008, "site name (m)")

points(tmp2, din.c.ci95[2,tmp1], pch="o", font=2)
points(tmp2, din.c.ci95[1,tmp1], pch="-", font=2)
points(tmp2, din.c.ci95[3,tmp1], pch="-", font=2)

segments(tmp2, din.c.ci95[1,tmp1], tmp2, din.c.ci95[3,tmp1], lwd=2)  
   
   
 
 
 Cross-validation via posterior prediction of  \(\hat{p}^B_{\text{med}}\)  (an  example in ecology ) 
  Can  do this for  \(\hat{p}^B_{\text{med}}\)  = median{  \(y_i/n_i\)  } (but not for median{  \(y^\ast_i/n_i\)  }) and for  \(i_{(15)} = \{i: \hat{p}^B_i=\hat{p}^B_{(15)}\}\)  and  \(i_{(16)}\) . 
 Due to the discreteness of  \(y_i\) , the true 50th percentile among  \(S\) =30 sites could be a value shared identically among more than 2 sites. Here, for pure cross-validation (i.e. not necessarily the true 50th percentile technically defined for a distribution), we take 
  \[
\hat{p}^B_{\text{med}} = \frac{\hat{p}^B_{(15)}+\hat{p}^B_{(16)}}{2}
\]  
 where the subscript “ \((i)\) ”
denotes the  \(i\) th sorted value, and
sorting is done by a combination of the value of  \(\hat{p}^B_i\)  and the corresponding site label,  irrespective of whether either  \(\hat{p}^B_{(15)}\)  or  \(\hat{p}^B_{(16)}\)  is equal to another site’s value . For example, suppose 
  \[
\hat{p}^B_{(14)} = \hat{p}^B_{(15)} < \hat{p}^B_{(16)} < \hat{p}^B_{(17)}
\]  
 with “(14), (15), (16)” corresponding to “614, 615, 616”
respectively in alphabetical order. Then, even though “614” would’ve been another contributor to a technically defined 50th percentile, we report 
 
 our  median  (for cross-validation purposes only) is the
mean of  \(\hat{p}^B_{(15)}\)  and
 \(\hat{p}^B_{(16)}\)  corresponding to
“614, 615.” 
 
   
  pBhat <- site[order(site$siteLabNIP),"siteLabNIP",drop=FALSE] 
medpBhat <- mean(pBhat[15:16,])
medpBhat.site <- rownames(pBhat)[15:16]
medpBhat # observed median  
  ## [1] 0.364  
  medpBhat.site # observed median site(s)  
  ## [1] "     601" "     613"  
  library(parallel)

y.c <- mcmapply( rbinom, 
          site$siteLabNymphs, 
          pB.c.ls, 
          MoreArgs = list(n=nrow(pB.c)),
          mc.cores=6)

phat.c <- t( t(y.c)/site$siteLabNymphs )

find.med <- function(vect, rname, loc){
    ord <- order(vect)
    mid <- vect[ord][loc]
    md <- mean( mid )
    mdname <- rname[ord][loc]
    md <- data.frame( c(mid,md),
                      check.names=FALSE,
                      row.names=c(mdname,"median"))
    colnames(md) <- "pBhat"
    return(md)
}

phat.med.c <- apply(phat.c, 1, 
                       find.med, 
                       rname=as.character(site$siteName),
                       loc=c(15:16))  
  tmp <- sapply(
        phat.med.c,
        function(df){
          return(df[3,1])
        })

hist( tmp,
      main=paste("posterior predictive distr'n for median{naive NIP estimates}\nred = observed median (",
                 medpBhat.site[1], ",", medpBhat.site[2], ")"),
      cex.main=1, font.main=1,
      xlab=expression(paste("median ", hat(p)[B]))
)

abline(v=medpBhat, col="red")  
   
  sum( tmp > medpBhat ) / length(tmp) # 1-sided posterior predictive p  
  ## [1] 0.1336  
  quantile(tmp, prob=c(.05,.95)) # 90% predictive interval  
  ##        5%       95% 
## 0.2727273 0.3798077  
  phat.med.c.site <- t( sapply(phat.med.c,
                                    function(x){ rownames(x)[1:2] },
                                    simplify="array" ))  
head(phat.med.c.site)  
  ##        [,1]   [,2]         
## [1,] " 619" " 617"    
## [2,] " 630" " 606"  
## [3,] " 601" " 614"       
## [4,] " 630" " 625"  
## [5,] " 601" " 611"      
## [6,] " 610" " 624"  
  phat.med.c.site.tab <- apply(phat.med.c.site, 2, table)

phat.med.c.site15 <- sort(phat.med.c.site.tab[,1], decreasing=TRUE)
phat.med.c.site16 <- sort(phat.med.c.site.tab[,2], decreasing=TRUE)  
  tmp <- names(phat.med.c.site15)==medpBhat.site[1] 
tmp1 <- rep("grey",S)
tmp1[tmp] <- "red"

par(mfrow=c(2,1))
par(las=2) # make label text perpendicular to axis
par(mgp=c(3,1,0)) # default
par(mar=c(10,5,4,2)) # increase x-axis margin

barplot( phat.med.c.site15, col=tmp1 )
title("posterior predictive dist'n for site \nwith '15th largest' naive NIP estimate\nred = observed")

tmp <- names(phat.med.c.site16)==medpBhat.site[2] 
tmp1 <- rep("grey",S)
tmp1[tmp] <- "red"

barplot( phat.med.c.site16, col=tmp1 )
title("posterior predictive dist'n for site \nwith '16th largest' naive NIP estimate\nred = observed")  
   
 
 
 
 


 

 

 
 

 
 
