# Supplementary material for: Influences of Host Community Characteristics on Borrelia burgdorferi Infection Prevalence in Blacklegged Ticks
Source: PLoS One. 2017 Jan 17;12(1):e0167810. doi: 10.1371/journal.pone.0167810 (PMC5241014; doi:10.1371/journal.pone.0167810)
Supplement: S9 File — (HTML) [file pone.0167810.s009.html]

Supporting Information S9 for Vuong, Chiu, Smouse, Fonseca, Brisson, Morin, and Ostfeld (2016). PLOS ONE.


# Supporting Information S9 for Vuong, Chiu, Smouse, Fonseca, Brisson, Morin, and Ostfeld (2016). *PLOS ONE.*

---

```
library(coda)
library(runjags)

head(tick2)
```

```
##                 n lyme his
##           901  21    4   3
##           902  39    7   6
##           903  29    3   3
##           904  25    7   5
##           606  11    3   3
##           609  28    4   3
##           618  39    6   5
##           908  24    5   5
##           909  31    2   2
##           910  17    0   0
##           911  28    7   3
##           912  33    7   6
##           627  42    9   6
##           914  22    8   6
##           628  38    3   3
##           629  26    3   3
##           630  28    6   5
##           918  54   19  16
```

```
print(dat.a) # covariates have been centered
```

```
## $S
## [1] 18
## 
## $n
##            901            902            903            904            606 
##             21             39             29             25             11 
##            609            618            908            909            910 
##             28             39             24             31             17 
##            911            912            627            914            628 
##             28             33             42             22             38 
##            629            630            918 
##             26             28             54 
## 
## $z
##                [,1] [,2] [,3] [,4] [,5] [,6] [,7] [,8] [,9] [,10] [,11]
##           901     0    0    1    0    0    1    0    0    0     0     0
##           902     0    1    0    0    0    0    0    0    0     0     0
##           903     1    0    0    1    0    0    1    0    0     0     0
##           904     0    0    0    0    0    0    0    0    0     1     1
##           606     0    0    1    0    0    0    0    0    1     0     1
##           609     0    0    0    0    0    0    0    0    0     0     1
##           618     0    0    0    0    0    0    0    0    0     0     0
##           908     0    1    0    0    0    1    0    0    0     0     0
##           909     0    0    1    0    0    0    0    0    0     0     0
##           910     0    0    0    0    0    0    0    0    0     0     0
##           911     0    0    0    0    0    1    0    0    0     0     0
##           912     1    1    0    0    0    0    0    1    0     0     0
##           627     0    0    0    0    0    0    0    1    0     0     1
##           914     0    0    0    1    0    0    0    1    1     0     1
##           628     0    0    0    0    0    1    0    0    0     0     0
##           629     0    0    0    1    0    0    0    0    0     0     1
##           630     0    0    0    0    0    0    0    1    0     1     0
##           918     1    0    0    0    0    0    0    1    0     0     0
##                [,12] [,13] [,14] [,15] [,16] [,17] [,18] [,19] [,20] [,21]
##           901      1     0     0     0     1     0     0     0     0     0
##           902      0     0     0     0     0     0     0     1     0     1
##           903      0     0     0     0     0     0     0     0     0     0
##           904      1     0     0     0     0     0     0     1     0     0
##           606   -999  -999  -999  -999  -999  -999  -999  -999  -999  -999
##           609      1     0     0     1     0     0     0     0     0     0
##           618      0     0     0     1     0     0     1     1     0     0
##           908      0     0     0     1     0     0     0     1     0     0
##           909      0     0     0     0     0     0     0     0     0     0
##           910      0     0     0     0     0     0  -999  -999  -999  -999
##           911      0     0     0     1     0     0     0     1     1     1
##           912      0     0     0     1     0     0     0     0     0     1
##           627      0     0     0     0     0     0     1     0     1     0
##           914      0     0     1     0     0     0     1     0     1     1
##           628      0     0     0     1     0     0     0     0     0     0
##           629      0     0     0     0     0     0     1     0     0     0
##           630      1     0     0     0     1     0     0     0     0     0
##           918      1     0     1     0     0     1     0     0     1     1
##                [,22] [,23] [,24] [,25] [,26] [,27] [,28] [,29] [,30] [,31]
##           901   -999  -999  -999  -999  -999  -999  -999  -999  -999  -999
##           902      0     1     0     0     0     0     0     1     1     0
##           903      0     0     0     0     0     0     0     0  -999  -999
##           904      1     0     1     1  -999  -999  -999  -999  -999  -999
##           606   -999  -999  -999  -999  -999  -999  -999  -999  -999  -999
##           609      0     0     0     1     0     0     0  -999  -999  -999
##           618      0     0     0     0     0     1     0     0     0     1
##           908      0     0     1  -999  -999  -999  -999  -999  -999  -999
##           909      1     0     0     0     0     0     0     0     0     0
##           910   -999  -999  -999  -999  -999  -999  -999  -999  -999  -999
##           911      1     0     0     0     0     0     1  -999  -999  -999
##           912      1     0     0     0     0     0     0     0     0     1
##           627      0     0     0     1     0     0     0     0     0     1
##           914      0  -999  -999  -999  -999  -999  -999  -999  -999  -999
##           628      0     0     0     1     0     0     0     0     0     0
##           629      0     0     0     0     0  -999  -999  -999  -999  -999
##           630      1     0     0     0     0     0     1  -999  -999  -999
##           918      0     0     0     0     0     0     1     0     1     0
##                [,32] [,33] [,34] [,35] [,36] [,37] [,38] [,39] [,40] [,41]
##           901   -999  -999  -999  -999  -999  -999  -999  -999  -999  -999
##           902      0     0     0     0     0     0     0     1  -999  -999
##           903   -999  -999  -999  -999  -999  -999  -999  -999  -999  -999
##           904   -999  -999  -999  -999  -999  -999  -999  -999  -999  -999
##           606   -999  -999  -999  -999  -999  -999  -999  -999  -999  -999
##           609   -999  -999  -999  -999  -999  -999  -999  -999  -999  -999
##           618      0     0     0     0     0     0     1     0  -999  -999
##           908   -999  -999  -999  -999  -999  -999  -999  -999  -999  -999
##           909   -999  -999  -999  -999  -999  -999  -999  -999  -999  -999
##           910   -999  -999  -999  -999  -999  -999  -999  -999  -999  -999
##           911   -999  -999  -999  -999  -999  -999  -999  -999  -999  -999
##           912      0     0  -999  -999  -999  -999  -999  -999  -999  -999
##           627      1     1     0     0     0     0     0     0     0     0
##           914   -999  -999  -999  -999  -999  -999  -999  -999  -999  -999
##           628      0     0     0     0     0     0     0  -999  -999  -999
##           629   -999  -999  -999  -999  -999  -999  -999  -999  -999  -999
##           630   -999  -999  -999  -999  -999  -999  -999  -999  -999  -999
##           918      0     1     0     1     1     0     1     1     1     1
##                [,42] [,43] [,44] [,45] [,46] [,47] [,48] [,49] [,50] [,51]
##           901   -999  -999  -999  -999  -999  -999  -999  -999  -999  -999
##           902   -999  -999  -999  -999  -999  -999  -999  -999  -999  -999
##           903   -999  -999  -999  -999  -999  -999  -999  -999  -999  -999
##           904   -999  -999  -999  -999  -999  -999  -999  -999  -999  -999
##           606   -999  -999  -999  -999  -999  -999  -999  -999  -999  -999
##           609   -999  -999  -999  -999  -999  -999  -999  -999  -999  -999
##           618   -999  -999  -999  -999  -999  -999  -999  -999  -999  -999
##           908   -999  -999  -999  -999  -999  -999  -999  -999  -999  -999
##           909   -999  -999  -999  -999  -999  -999  -999  -999  -999  -999
##           910   -999  -999  -999  -999  -999  -999  -999  -999  -999  -999
##           911   -999  -999  -999  -999  -999  -999  -999  -999  -999  -999
##           912   -999  -999  -999  -999  -999  -999  -999  -999  -999  -999
##           627      1  -999  -999  -999  -999  -999  -999  -999  -999  -999
##           914   -999  -999  -999  -999  -999  -999  -999  -999  -999  -999
##           628   -999  -999  -999  -999  -999  -999  -999  -999  -999  -999
##           629   -999  -999  -999  -999  -999  -999  -999  -999  -999  -999
##           630   -999  -999  -999  -999  -999  -999  -999  -999  -999  -999
##           918      0     0     0     0     1     1     1     0     0     0
##                [,52] [,53] [,54]
##           901   -999  -999  -999
##           902   -999  -999  -999
##           903   -999  -999  -999
##           904   -999  -999  -999
##           606   -999  -999  -999
##           609   -999  -999  -999
##           618   -999  -999  -999
##           908   -999  -999  -999
##           909   -999  -999  -999
##           910   -999  -999  -999
##           911   -999  -999  -999
##           912   -999  -999  -999
##           627   -999  -999  -999
##           914   -999  -999  -999
##           628   -999  -999  -999
##           629   -999  -999  -999
##           630   -999  -999  -999
##           918      0     0     0
## 
## $t
##                [,1] [,2] [,3] [,4] [,5] [,6] [,7] [,8] [,9] [,10] [,11]
##           901     0    0    1    0    0    1    0    0    0     0     0
##           902     0    1    0    0    0    0    0    0    0     0     0
##           903     1    0    0    1    0    0    1    0    0     0     0
##           904     0    0    0    0    0    0    0    0    0     1     1
##           606     0    0    1    0    0    0    0    0    1     0     1
##           609     0    0    0    0    0    0    0    0    0     0     0
##           618     0    0    0    0    0    0    0    0    0     0     0
##           908     0    1    0    0    0    1    0    0    0     0     0
##           909     0    0    1    0    0    0    0    0    0     0     0
##           910     0    0    0    0    0    0    0    0    0     0     0
##           911     0    0    0    0    0    0    0    0    0     0     0
##           912     1    0    0    0    0    0    0    1    0     0     0
##           627     0    0    0    0    0    0    0    1    0     0     0
##           914     0    0    0    0    0    0    0    1    1     0     1
##           628     0    0    0    0    0    1    0    0    0     0     0
##           629     0    0    0    1    0    0    0    0    0     0     1
##           630     0    0    0    0    0    0    0    1    0     1     0
##           918     1    0    0    0    0    0    0    1    0     0     0
##                [,12] [,13] [,14] [,15] [,16] [,17] [,18] [,19] [,20] [,21]
##           901      1     0     0     0     0     0     0     0     0     0
##           902      0     0     0     0     0     0     0     1     0     1
##           903      0     0     0     0     0     0     0     0     0     0
##           904      1     0     0     0     0     0     0     1     0     0
##           606   -999  -999  -999  -999  -999  -999  -999  -999  -999  -999
##           609      1     0     0     1     0     0     0     0     0     0
##           618      0     0     0     1     0     0     1     1     0     0
##           908      0     0     0     1     0     0     0     1     0     0
##           909      0     0     0     0     0     0     0     0     0     0
##           910      0     0     0     0     0     0  -999  -999  -999  -999
##           911      0     0     0     1     0     0     0     0     1     0
##           912      0     0     0     1     0     0     0     0     0     1
##           627      0     0     0     0     0     0     0     0     1     0
##           914      0     0     0     0     0     0     1     0     1     1
##           628      0     0     0     1     0     0     0     0     0     0
##           629      0     0     0     0     0     0     1     0     0     0
##           630      1     0     0     0     0     0     0     0     0     0
##           918      1     0     0     0     0     0     0     0     1     1
##                [,22] [,23] [,24] [,25] [,26] [,27] [,28] [,29] [,30] [,31]
##           901   -999  -999  -999  -999  -999  -999  -999  -999  -999  -999
##           902      0     0     0     0     0     0     0     1     1     0
##           903      0     0     0     0     0     0     0     0  -999  -999
##           904      0     0     1     0  -999  -999  -999  -999  -999  -999
##           606   -999  -999  -999  -999  -999  -999  -999  -999  -999  -999
##           609      0     0     0     1     0     0     0  -999  -999  -999
##           618      0     0     0     0     0     1     0     0     0     0
##           908      0     0     1  -999  -999  -999  -999  -999  -999  -999
##           909      1     0     0     0     0     0     0     0     0     0
##           910   -999  -999  -999  -999  -999  -999  -999  -999  -999  -999
##           911      0     0     0     0     0     0     1  -999  -999  -999
##           912      1     0     0     0     0     0     0     0     0     1
##           627      0     0     0     1     0     0     0     0     0     0
##           914      0  -999  -999  -999  -999  -999  -999  -999  -999  -999
##           628      0     0     0     1     0     0     0     0     0     0
##           629      0     0     0     0     0  -999  -999  -999  -999  -999
##           630      1     0     0     0     0     0     1  -999  -999  -999
##           918      0     0     0     0     0     0     1     0     1     0
##                [,32] [,33] [,34] [,35] [,36] [,37] [,38] [,39] [,40] [,41]
##           901   -999  -999  -999  -999  -999  -999  -999  -999  -999  -999
##           902      0     0     0     0     0     0     0     1  -999  -999
##           903   -999  -999  -999  -999  -999  -999  -999  -999  -999  -999
##           904   -999  -999  -999  -999  -999  -999  -999  -999  -999  -999
##           606   -999  -999  -999  -999  -999  -999  -999  -999  -999  -999
##           609   -999  -999  -999  -999  -999  -999  -999  -999  -999  -999
##           618      0     0     0     0     0     0     1     0  -999  -999
##           908   -999  -999  -999  -999  -999  -999  -999  -999  -999  -999
##           909   -999  -999  -999  -999  -999  -999  -999  -999  -999  -999
##           910   -999  -999  -999  -999  -999  -999  -999  -999  -999  -999
##           911   -999  -999  -999  -999  -999  -999  -999  -999  -999  -999
##           912      0     0  -999  -999  -999  -999  -999  -999  -999  -999
##           627      1     1     0     0     0     0     0     0     0     0
##           914   -999  -999  -999  -999  -999  -999  -999  -999  -999  -999
##           628      0     0     0     0     0     0     0  -999  -999  -999
##           629   -999  -999  -999  -999  -999  -999  -999  -999  -999  -999
##           630   -999  -999  -999  -999  -999  -999  -999  -999  -999  -999
##           918      0     1     0     0     1     0     1     1     1     1
##                [,42] [,43] [,44] [,45] [,46] [,47] [,48] [,49] [,50] [,51]
##           901   -999  -999  -999  -999  -999  -999  -999  -999  -999  -999
##           902   -999  -999  -999  -999  -999  -999  -999  -999  -999  -999
##           903   -999  -999  -999  -999  -999  -999  -999  -999  -999  -999
##           904   -999  -999  -999  -999  -999  -999  -999  -999  -999  -999
##           606   -999  -999  -999  -999  -999  -999  -999  -999  -999  -999
##           609   -999  -999  -999  -999  -999  -999  -999  -999  -999  -999
##           618   -999  -999  -999  -999  -999  -999  -999  -999  -999  -999
##           908   -999  -999  -999  -999  -999  -999  -999  -999  -999  -999
##           909   -999  -999  -999  -999  -999  -999  -999  -999  -999  -999
##           910   -999  -999  -999  -999  -999  -999  -999  -999  -999  -999
##           911   -999  -999  -999  -999  -999  -999  -999  -999  -999  -999
##           912   -999  -999  -999  -999  -999  -999  -999  -999  -999  -999
##           627      1  -999  -999  -999  -999  -999  -999  -999  -999  -999
##           914   -999  -999  -999  -999  -999  -999  -999  -999  -999  -999
##           628   -999  -999  -999  -999  -999  -999  -999  -999  -999  -999
##           629   -999  -999  -999  -999  -999  -999  -999  -999  -999  -999
##           630   -999  -999  -999  -999  -999  -999  -999  -999  -999  -999
##           918      0     0     0     0     1     1     1     0     0     0
##                [,52] [,53] [,54]
##           901   -999  -999  -999
##           902   -999  -999  -999
##           903   -999  -999  -999
##           904   -999  -999  -999
##           606   -999  -999  -999
##           609   -999  -999  -999
##           618   -999  -999  -999
##           908   -999  -999  -999
##           909   -999  -999  -999
##           910   -999  -999  -999
##           911   -999  -999  -999
##           912   -999  -999  -999
##           627   -999  -999  -999
##           914   -999  -999  -999
##           628   -999  -999  -999
##           629   -999  -999  -999
##           630   -999  -999  -999
##           918      0     0     0
## 
## $x
##                hostHindexAll   siteRich log_hostPELEprop log_hostTASTprop
##           901   -0.097046398 -1.3888889       0.40907332       0.48565586
##           902    0.272162280  2.6111111      -0.08541871       1.36795679
##           903    0.385641216 -0.3888889       0.05216835       0.02570484
##           904    0.183292394 -1.3888889      -0.16901336       1.86836594
##           606    0.070597698 -0.3888889       0.16823427      -0.46471293
##           609   -0.284795351  0.6111111       0.54524110       0.69222840
##           618   -0.011432566  0.6111111       0.40603723       0.47364198
##           908   -0.053461884 -1.3888889       0.36578127      -1.45317111
##           909    0.427782103  0.6111111      -0.34546208       0.48840064
##           910    0.005119898  2.6111111      -0.97616275      -1.47447684
##           911    0.080986552 -1.3888889       0.19676034       0.38293510
##           912    0.053186513  0.6111111       0.23583963      -0.44432882
##           627   -0.267199821 -2.3888889       0.52236108       0.81469433
##           914   -0.326037762 -1.3888889       0.54200803      -1.66273794
##           628   -0.258415449  0.6111111      -2.24357992      -1.20119814
##           629    0.224453445  0.6111111      -0.62198891       0.19768941
##           630   -0.004888057 -0.3888889       0.36729477       0.21261974
##           918   -0.399944812  1.6111111       0.63082635      -0.30926725
##                log_hostBLBRprop
##           901         0.3087519
##           902        -0.4328582
##           903         1.6280253
##           904         0.7988039
##           606        -0.1907819
##           609        -0.3626169
##           618         0.5748170
##           908         0.9986955
##           909         0.9097081
##           910        -0.1055291
##           911         0.7688322
##           912        -0.2405409
##           627        -1.0778400
##           914         0.3607813
##           628        -2.2897810
##           629         0.8558357
##           630        -0.6213197
##           918        -1.8829834
```

## Preliminary JAGS model

### Hypothetical model as if PCR and RLB tests had been perfect

\[
\begin{aligned}
\text{[2.2a]} &&
\left. z\_{ij} \right| p\_i^B \sim \text{(ind) Bernoulli}(p\_i^B) \\
&& \log \frac{p\_i^B}{1-p\_i^B} = \alpha\_0 + \boldsymbol{x}\_i ' \boldsymbol{\alpha} + \eta\_i \\
&& \eta\_i \sim \text{(iid) }N(0, \tau^2) \\
\implies \text{[2.3]} &&
\left. \text{logit } p\_i^B \right| \alpha\_0, \boldsymbol{\alpha}, \tau^2, \boldsymbol{x}\_i \sim \text{(ind) } N(\alpha\_0 + \boldsymbol{x}\_i ' \boldsymbol{\alpha}, \tau^2) \\
&& \log \frac{p\_i^H / p\_i^B}{1- p\_i^H / p\_i^B} = \mu\_i = 0 + \boldsymbol{x}\_i ' \boldsymbol{\gamma} + \xi\_i \\
\implies && p\_i^H = \frac{p\_i^B e^{\mu\_i}}{1 + e^{\mu\_i}} \\\
\text{[3.1a]} &&
\left. t\_{ij} \right| p\_i^B, p\_i^c \sim \text{(ind) Bernoulli}(p\_i^B p\_i^c) \\
\implies \text{[2.5a]} &&
\left. \text{logit } p\_i^c \right| \gamma\_0, \boldsymbol{\gamma}, \omega^2, \boldsymbol{x}\_i \sim \text{(ind) } N(\boldsymbol{x}\_i ' \boldsymbol{\gamma}, \omega^2) \\
\end{aligned}
\]

```
jags.script.a <- "

model{ # hypothetical, all covariates 
  
  # ---------- definitions
  
  tau <- 1/sqrt(tausq.inv) # SD in eq 2.3
  omega <- 1/sqrt(omsq.inv) # SD in eq 2.5a
  alph[2] <- -999
  gam[2] <- -999
  
  for(i in 1:S){

    pB[i] <- ilogit(logitpB[i])
    pC[i] <- ilogit(logitpC[i])
    
    nuL[i] <- alph0 + alph[1]*x[i,1] + inprod(alph[3:5], x[i,3:5]) # mean in eq 2.3
    nuH[i] <- gam0 + gam[1]*x[i,1] + inprod(gam[3:5], x[i,3:5]) # mean in eq 2.5a
     
    pH[i] <- pB[i]*pC[i] # inside eq 3.1a 
  }
  
  
  # ---------- likelihood
  
  for(i in 1:S){
    
    logitpB[i] ~ dnorm(nuL[i], tausq.inv) # eq 2.3
    
    logitpC[i] ~ dnorm(nuH[i], omsq.inv) # eq 2.5a
    
    for(j in 1:n[i]){

      z[i,j] ~ dbern(pB[i]) # eq 2.2a
      t[i,j] ~ dbern(pH[i]) # eq 3.1a
    }    
  }
  
  
  # ---------- priors
  
  tausq.inv ~ dgamma(1, .01)
  omsq.inv ~ dgamma(.1, .1) # poor mixing when more diffuse => weak identifiability
  
  alph0 ~ dnorm(0, .001)
  gam0 ~ dnorm(0, .001)
  alph[1] ~ dnorm(0, .001)
  gam[1] ~ dnorm(0, .001)

  for(k in 3:5){
    
    alph[k] ~ dnorm(0, .001)
    gam[k] ~ dnorm(0, .001)
        
  }
}
"
```

```
fit.a <- run.jags(jags.script.a, data=dat.a, n.chains=2,
                      inits=list(tausq.inv=1, omsq.inv=1),
                      modules='glm', # improves mixing
                      adapt=50000, burnin=20000, sample=5000, thin=10,
                      monitor=c(
                        "logitpB","logitpC","alph0","alph","gam0","gam","tau","omega",
                        "deviance","pd","dic"))
```

```
## module glm loaded
## module dic loaded
## Compiling rjags model...
## Calling the simulation using the rjags method...
## Adapting the model for 50000 iterations...
##   |++++++++++++++++++++++++++++++++++++++++++++++++++| 100%
## Burning in the model for 20000 iterations...
##   |**************************************************| 100%
## Running the model for 50000 iterations...
##   |**************************************************| 100%
## Extending 50000 iterations for pD/DIC estimates...
##   |**************************************************| 100%
## Simulation complete
## Calculating summary statistics...
## Note: The monitored variables 'alph[2]' and 'gam[2]' appear to
## be non-stochastic; they will not be included in the
## convergence diagnostic
## Calculating the Gelman-Rubin statistic for 51 variables....
## Finished running the simulation
## Warning message:
## The length of the initial values argument supplied found does not correspond to the number of chains specified.  Some initial values were recycled or ignored.
```

```
scans.a <- as.mcmc.list(fit.a)
scans.a.pooled <- rbind(scans.a[[1]], scans.a[[2]])
```

```
fit.a$runjags.version
```

```
## [1] "2.0.2-8"                      "R version 3.2.2 (2015-08-14)"
## [3] "unix"                         "RStudio"                     
## [5] "mac.binary.mavericks"         "2015-10-09 17:45:34"
```

```
fit.a$psrf$mpsrf # Gelman-Rubin convergence check
```

```
## [1] 1.029946
```

```
fit.a.dic.alt <- mean(scans.a.pooled[,"deviance"]) + var(scans.a.pooled[,"deviance"])/2
fit.a.dic.alt
```

```
## [1] 974.5282
```

```
par(mfrow=c(4,3))
traceplot(scans.a)
```

### Realistic model, all covariates

\[
\begin{aligned}
\text{[2.2a]} &&
\left. z\_{ij} \right| p\_i^B \sim \text{(ind) Bernoulli}(p\_i^B) \\
\text{[2.3]} &&
\left. \text{logit } p\_i^B \right| \alpha\_0, \boldsymbol{\alpha}, \tau^2, \boldsymbol{x}\_i \sim \text{(ind) } N(\alpha\_0 + \boldsymbol{x}\_i ' \boldsymbol{\alpha}, \tau^2) \\
\text{[3.1aa]} &&
\left. t\_{ij} \right| z\_{ij}, p\_i^c \sim \text{(ind) Bernoulli}(z\_{ij} p\_i^c) \\
\text{[2.5]} &&
\text{logit } p\_i^c \left| \gamma\_0, \boldsymbol{\gamma}, \omega^2, \boldsymbol{x}\_i \right. \sim \text{(ind) } N(\gamma\_0 + \boldsymbol{x}\_i ' \boldsymbol{\gamma}, \omega^2) \\
\end{aligned}
\]

```
jags.script.aa <- "

model{ # realistic, all covariates 
  
  # ---------- definitions

  alph[2] <- -999
  gam[2] <- -999
  
  tau <- 1/sqrt(tausq.inv) # SD in eq 2.3
  omega <- 1/sqrt(omsq.inv) # SD in eq 2.5
  
  for(i in 1:S){

    pB[i] <- ilogit(logitpB[i])
    pC[i] <- ilogit(logitpC[i])
    
    nuL[i] <- alph0 + alph[1]*x[i,1] + inprod(alph[3:5], x[i,3:5]) # mean in eq 2.3
    nuH[i] <- gam0 + gam[1]*x[i,1] + inprod(gam[3:5], x[i,3:5]) # mean in eq 2.5

    for(j in 1:n[i]){

      pH[i,j] <- z[i,j] * pC[i] # inside eq 3.1aa
    }
  }
  
  
  # ---------- likelihood
  
  for(i in 1:S){
    
    logitpB[i] ~ dnorm(nuL[i], tausq.inv) # eq 2.3
    logitpC[i] ~ dnorm(nuH[i], omsq.inv) # eq 2.5
    
    for(j in 1:n[i]){

      z[i,j] ~ dbern(pB[i]) # eq 2.2a 
      t[i,j] ~ dbern(pH[i,j]) # eq 3.1aa
    }    
  }
  
  
  # ---------- priors
  
  tausq.inv ~ dgamma(1, .01)
  omsq.inv ~ dgamma(1, .1) # poor mixing if more diffuse

  alph0 ~ dnorm(0, .001)
  gam0 ~ dnorm(0, .001)

  alph[1] ~ dnorm(0, .001)
  gam[1] ~ dnorm(0, .001)

  for(k in 3:5){
    
    alph[k] ~ dnorm(0, .001)
    gam[k] ~ dnorm(0, .001)
        
  }
}
"
```

```
fit.aa <- run.jags(jags.script.aa, data=dat.a, n.chains=2,
                      inits=list(tausq.inv=1, omsq.inv=1),
                      module='glm',
                      adapt=40000, burnin=40000, sample=2500, thin=25,
                      monitor=c(
                        "logitpB","logitpC","alph0","gam0","alph","gam","tau","omega",
                        "deviance","pd","dic"))
```

```
## module glm loaded
## module dic loaded
## Compiling rjags model...
## Calling the simulation using the rjags method...
## Adapting the model for 40000 iterations...
##   |++++++++++++++++++++++++++++++++++++++++++++++++++| 100%
## Burning in the model for 40000 iterations...
##   |**************************************************| 100%
## Running the model for 62500 iterations...
##   |**************************************************| 100%
## Extending 62500 iterations for pD/DIC estimates...
##   |**************************************************| 100%
## Simulation complete
## Calculating summary statistics...
## Note: The monitored variables 'alph[2]' and 'gam[2]' appear to be non-stochastic; they will
## not be included in the convergence diagnostic
## Calculating the Gelman-Rubin statistic for 51 variables....
## Finished running the simulation
## Warning message:
## The length of the initial values argument supplied found does not correspond to the number of chains specified.  Some initial values were recycled or ignored.
```

```
scans.aa <- as.mcmc.list(fit.aa)
scans.aa.pooled <- rbind(scans.aa[[1]], scans.aa[[2]])
```

```
fit.aa$psrf$mpsrf # Gelman-Rubin convergence check
```

```
## [1] 1.032063
```

```
fit.aa.dic.alt <- mean(scans.aa.pooled[,"deviance"]) + var(scans.aa.pooled[,"deviance"])/2
fit.aa.dic.alt
```

```
## [1] 625.5422
```

```
pval <- apply(scans.aa.pooled[,c(39,41:44,46:48)], 2, ecdf) # placeholder
pval <- sapply(pval,do.call,args=list(0))
pval <- data.frame(lefttail=pval,righttail=1-pval)
tmp <- subset(pval,lefttail<.5)
pval <- rbind(tmp, subset(pval,righttail<.5))

print(pval)
```

```
##         lefttail righttail
## alph[3]   0.0036    0.9964
## gam[1]    0.3226    0.6774
## alph[1]   0.6454    0.3546
## alph[4]   0.5602    0.4398
## alph[5]   0.7254    0.2746
## gam[3]    0.9124    0.0876
## gam[4]    0.9840    0.0160
## gam[5]    0.9036    0.0964
```

```
par(mfrow=c(4,3))
traceplot(scans.aa)
```

Tail probabilities and traceplots for \(\alpha\)s and \(\gamma\)s suggest no evidence that \(x\_4\) is relevant to \(p^B\) (lyme prevalence).

### Reduced realistic model

```
jags.script.aa4 <- "

model{ # realistic, set alpha_4=gamma_2=0 (in addition to alpha_2=0)
  
  # ---------- definitions
  
  tau <- 1/sqrt(tausq.inv) # SD in eq 2.3
  omega <- 1/sqrt(omsq.inv) # SD in eq 2.5
  alph[2] <- -999
  alph[4] <- -999
  gam[2] <- -999
  
  for(i in 1:S){

    pB[i] <- ilogit(logitpB[i])
    pC[i] <- ilogit(logitpC[i])
    
    nuL[i] <- alph0 + alph[1]*x[i,1] + alph[3]*x[i,3] + alph[5]*x[i,5] # mean in eq 2.3
    nuH[i] <- gam0 + gam[1]*x[i,1] + inprod(gam[3:5], x[i,3:5]) # mean in eq 2.5

    for(j in 1:n[i]){

      pH[i,j] <- z[i,j] * pC[i] # inside eq 3.1aa
    }
  }
  
  
  # ---------- likelihood
  
  for(i in 1:S){
    
    logitpB[i] ~ dnorm(nuL[i], tausq.inv) # eq 2.3
    logitpC[i] ~ dnorm(nuH[i], omsq.inv) # eq 2.5
    
    for(j in 1:n[i]){

      z[i,j] ~ dbern(pB[i]) # eq 2.2a 
      t[i,j] ~ dbern(pH[i,j]) # eq 3.1aa
    }    
  }
  
  
  # ---------- priors
  
  tausq.inv ~ dgamma(1, .01)
  omsq.inv ~ dgamma(1, .1)
  
  alph0 ~ dnorm(0, .001)
  gam0 ~ dnorm(0, .001)
  alph[1] ~ dnorm(0, .001)
  alph[3] ~ dnorm(0, .001)
  alph[5] ~ dnorm(0, .001)
  gam[1] ~ dnorm(0, .001)

  for(k in 3:5){
    gam[k] ~ dnorm(0, .001)
        
  }
}
"
```

```
fit.aa4 <- run.jags(jags.script.aa4, data=dat.a, n.chains=2,
                      inits=list(tausq.inv=1, omsq.inv=1),
                      module='glm',
                      adapt=30000, burnin=20000, sample=2000, thin=20,
                      monitor=c(
                        "logitpB","logitpC","alph0","gam0","alph[1]","alph[3]","alph[5]",
                        "gam","tau","omega","deviance","pd","dic"))
```

```
## Compiling rjags model...
## Calling the simulation using the rjags method...
## Adapting the model for 30000 iterations...
##   |++++++++++++++++++++++++++++++++++++++++++++++++++| 100%
## Burning in the model for 20000 iterations...
##   |**************************************************| 100%
## Running the model for 40000 iterations...
##   |**************************************************| 100%
## Extending 40000 iterations for pD/DIC estimates...
##   |**************************************************| 100%
## Simulation complete
## Calculating summary statistics...
## Note: The monitored variable 'gam[2]' appears to be non-stochastic; it will not be included in
## the convergence diagnostic
## Calculating the Gelman-Rubin statistic for 49 variables....
## Finished running the simulation
## Warning message:
## The length of the initial values argument supplied found does not correspond to the number of chains specified.  Some initial values were recycled or ignored.
```

```
scans.aa4 <- as.mcmc.list(fit.aa4)
scans.aa4.pooled <- rbind(scans.aa4[[1]], scans.aa4[[2]])
```

```
fit.aa4$psrf$mpsrf # Gelman-Rubin convergence check
```

```
## [1] 1.028398
```

```
fit.aa4.dic.alt <- mean(scans.aa4.pooled[,"deviance"]) + var(scans.aa4.pooled[,"deviance"])/2
fit.aa4.dic.alt
```

```
## [1] 623.0716
```

```
pval <- apply(scans.aa4.pooled[,c(39:42,44:46)], 2, ecdf) # placeholder
pval <- sapply(pval,do.call,args=list(0))
pval <- data.frame(lefttail=pval,righttail=1-pval,
                   median=apply(scans.aa4.pooled[,c(39:42,44:46)], 2, median))
tmp <- subset(pval,lefttail<.5)
pval <- rbind(tmp, subset(pval,righttail<.5))

print(pval)
```

```
##         lefttail righttail      median
## alph[3]  0.00100   0.99900  0.66352679
## gam[1]   0.28625   0.71375  1.66887685
## alph[1]  0.70025   0.29975 -0.40562644
## alph[5]  0.70650   0.29350 -0.09916881
## gam[3]   0.90325   0.09675 -1.92911368
## gam[4]   0.98275   0.01725 -0.80634533
## gam[5]   0.89100   0.10900 -0.56579151
```

**NOTE:**

Taking \(\omega^{-2}\sim\) Gamma(1, .01) gives basically same DIC but poorer mixing (i.e., not our model of choice):

```
## > fit.aa4$psrf$mpsrf # with dgamma(1, .01)
## [1] 1.066223
## > fit.aa4.dic.alt # with dgamma(1, .01)
## [1] 623.7575
```

### Model diagnosis for reduced realistic model

Now we diagnose the “residuals” \(\eta\) and \(\xi\) (note that normality of posterior of each \(\eta\_i\) and \(\xi\_i\) is irrelevant):

```
eta <- scans.aa4.pooled[,1:18] # just logit(pB)
eta <- eta - scans.aa4.pooled[,"alph0"] # logit(pB) - alph0
eta <- eta - scans.aa4.pooled[,39:41] %*% t(dat.a$x[,c(1,3,5)]) # actual eta

xi <- scans.aa4.pooled[,19:36] # just logit(pC)
xi <- xi - scans.aa4.pooled[,"gam0"] # logit(pC) - gam0
xi <- xi - scans.aa4.pooled[,c(42,44:46)] %*% t(dat.a$x[,c(1,3:5)]) # actual xi
```

```
par(mfrow=c(3,1))
par(cex.lab=1.5)
par(cex.main=2)

zoomlim <- .8
densfac <- 1.5

covar <- colnames(dat.a$x)[c(1,3,5)]
dens <- apply(eta, 2, density) 

dens.x <- NULL
dens.y <- NULL

for(i in 1:nrow(dat.a$x)){
  
  dens.x <- cbind(dens.x, dens[[i]]$x)
  dens.y <- cbind(dens.y, dens[[i]]$y)
}

for(k in covar){
  
  plot(1:2, type="n", xlim=range(dat.a$x[,k]), ylim=range(dens.x),
    xlab=paste("2009",k), ylab="eta posterior") # dummy plot
  abline(h=0)
  
  for(i in 1:nrow(dat.a$x))
    points(rep(dat.a$x[i,k],length(dens.x[,i])), dens.x[,i], cex=dens.y[,i]*densfac)
}

tmp <- rbind( c(-.4,.42),
              c(-1,.8),
              c(-2.5,2)
              )

rownames(tmp) <- covar

for(k in covar){
  
  plot(1:2, type="n", ylim=c(-zoomlim,zoomlim),
    xlim=tmp[k,], main="omit influential resid, zoomed in",   
    xlab=paste("2009",k), ylab="eta posterior") # dummy plot
  abline(h=0)
  
  for(i in 1:nrow(dat.a$x))
    points(rep(dat.a$x[i,k],length(dens.x[,i])), dens.x[,i], cex=dens.y[,i]*densfac)
}

plot(1:nrow(dat.a$x), type="n", ylim=range(dens.x),
  xlab="2009 site i", ylab="eta posterior") # dummy plot
abline(h=0)
  
for(i in 1:nrow(dat.a$x))
  points(rep(i,length(dens.x[,i])), dens.x[,i], cex=dens.y[,i]*3)

plot(1:nrow(dat.a$x), type="n", ylim=c(-zoomlim,zoomlim),
  xlab="2009 site i", ylab="eta posterior", main="zoomed in") # dummy plot
abline(h=0)
  
for(i in 1:nrow(dat.a$x))
  points(rep(i,length(dens.x[,i])), dens.x[,i], cex=dens.y[,i]*3)
```

```
par(mfrow=c(4,1))
par(cex.lab=1.5)
par(cex.main=2)

zoomlim <- 1.8
densfac <- 5

covar <- colnames(dat.a$x)[c(1,3:5)]
dens <- apply(xi, 2, density) 

dens.x <- NULL
dens.y <- NULL

for(i in 1:nrow(dat.a$x)){
  
  dens.x <- cbind(dens.x, dens[[i]]$x)
  dens.y <- cbind(dens.y, dens[[i]]$y)
}

tmp <- rbind( c(-.4,.42),
              c(-1,.8),
              c(-1.6, 2),
              c(-2.5,2)
              )

rownames(tmp) <- covar

for(k in covar){
  
  plot(1:2, type="n", xlim=range(dat.a$x[,k]), ylim=range(dens.x),
    xlab=paste("2009",k), ylab="xi posterior") # dummy plot
  abline(h=0)
  
  for(i in 1:nrow(dat.a$x))
    points(rep(dat.a$x[i,k],length(dens.x[,i])), dens.x[,i], cex=dens.y[,i]*densfac)
}

for(k in covar){
  
  plot(1:2, type="n", ylim=c(-zoomlim,zoomlim),
    xlim=tmp[k,], main="omit influential resid, zoomed in",   
    xlab=paste("2009",k), ylab="xi posterior") # dummy plot
  abline(h=0)
  
  for(i in 1:nrow(dat.a$x))
    points(rep(dat.a$x[i,k],length(dens.x[,i])), dens.x[,i], cex=dens.y[,i]*densfac)
}

plot(1:nrow(dat.a$x), type="n", ylim=range(dens.x),
  xlab="2009 site i", ylab="xi posterior") # dummy plot
abline(h=0)
  
for(i in 1:nrow(dat.a$x))
  points(rep(i,length(dens.x[,i])), dens.x[,i], cex=dens.y[,i]*densfac)

plot(1:nrow(dat.a$x), type="n", ylim=c(-zoomlim,zoomlim),
  xlab="2009 site i", ylab="xi posterior", main="zoomed in") # dummy plot
abline(h=0)
  
for(i in 1:nrow(dat.a$x))
  points(rep(i,length(dens.x[,i])), dens.x[,i], cex=dens.y[,i]*densfac)
```

The reduced model shows no mixing issues nor residual plots that are undesirable => can use these results as final inference.

## Compare fit (posterior SD) and goodness-of-fit

| model | z | t | realistic? | reduced? | DIC |
| --- | --- | --- | --- | --- | --- |
| aa4 | `Y` | `Y` | `Y` | `Y` | 623 |
| aa | `Y` | `Y` | `Y` | N | 626 |
| a | `Y` | `Y` | N | N | 975 |

```
# rbind(names(fit.a$summary$stat[,"SD"]),names(fit.aa$summary$stat[,"SD"]))

pBpCcolname <- names(fit.a$summary$stat[,"SD"])[1:36]
coef_prec_colname <- names(fit.a$summary$stat[,"SD"])[37:50]
paramcolname <- names(fit.a$summary$stat[,"SD"])[c(37:50, 1:36)]

library(boot)
```

```
## 
## Attaching package: 'boot'
```

```
## The following object is masked _by_ '.GlobalEnv':
## 
##     logit
```

```
merge(t(data.frame(c(mod="aa",inv.logit(fit.aa$summary$quant[pBpCcolname,"50%"])))),
      t(data.frame(c(mod="a",inv.logit(fit.a$summary$quant[pBpCcolname,"50%"])))),all=T) # posterior medians on original scale
```

```
##      mod        logitpB[1]        logitpB[2]        logitpB[3]
## 1     aa 0.211163275247097 0.155345068247715 0.134524153462952
## 2      a 0.182342324448995 0.147981952820653 0.124953228246418
##          logitpB[4]        logitpB[5]        logitpB[6]        logitpB[7]
## 1 0.140429107288182 0.193029661511495 0.242506503258002 0.196789888580971
## 2 0.128544100563349  0.18996837187152 0.199109869325763 0.171091774261795
##          logitpB[8]        logitpB[9]        logitpB[10]       logitpB[11]
## 1 0.201340051384161 0.110269455577213 0.0991930176992349 0.177362657665989
## 2 0.190536224957216 0.103543003645489 0.0955774205376872 0.158472246514399
##         logitpB[12]       logitpB[13]       logitpB[14]        logitpB[15]
## 1 0.200393239533982 0.257101546836149 0.260229738190181 0.0648013872829047
## 2 0.191639178872764  0.21793070157169 0.242289254084946 0.0680795472349941
##          logitpB[16]       logitpB[17]       logitpB[18]        logitpC[1]
## 1  0.103392721087901 0.221417647542933 0.319493405780531 0.685138043435341
## 2 0.0986105868205791 0.204073122038492 0.299226346464536                 1
##          logitpC[2]        logitpC[3]        logitpC[4]        logitpC[5]
## 1 0.897641218802985 0.876334952966617 0.745762635912904 0.937724567155127
## 2                 1                 1                 1                 1
##          logitpC[6]        logitpC[7]       logitpC[8]        logitpC[9]
## 1 0.612867821768345 0.699209062618992 0.90130529862341 0.955312240769761
## 2                 1                 1                1                 1
##         logitpC[10]       logitpC[11]       logitpC[12]       logitpC[13]
## 1 0.997571732818166 0.732274368638444 0.919748174688347 0.677207879099769
## 2                 1                 1                 1                 1
##         logitpC[14]       logitpC[15]       logitpC[16]       logitpC[17]
## 1 0.848948125288691 0.999932549009945 0.973570360326836 0.849396306393737
## 2                 1                 1                 1                 1
##         logitpC[18]
## 1 0.848079755132354
## 2                 1
```

```
merge(t(data.frame(c(mod="aa",fit.aa$summary$quant[coef_prec_colname,"50%"]))),
      t(data.frame(c(mod="a",fit.a$summary$quant[coef_prec_colname,"50%"]))),all=T) # posterior medians
```

```
##      mod             alph0             alph[1] alph[2]           alph[3]
## 1     aa -1.57256384148928  -0.328891217302297    -999 0.661457354530607
## 2      a -1.66777866196921 -0.0733381352659651    -999 0.644398632814859
##               alph[4]            alph[5]             gam0
## 1 -0.0265257218652091  -0.10719230233747 2.47096154271831
## 2 -0.0761303894066254 -0.152953480923531 49.4552450792682
##               gam[1] gam[2]            gam[3]             gam[4]
## 1   1.46965285769387   -999 -2.32918336952889 -0.847538264382159
## 2 -0.956604125251987   -999 -7.36384284695969  -3.00810240649183
##               gam[5]               tau             omega
## 1 -0.593893752427079 0.112297514337144 0.347958024557168
## 2  -4.03013261822275 0.136873304678068  2.04948520491781
```

```
merge(t(data.frame(c(mod="aa",fit.aa$summary$stat[paramcolname,"SD"]))),
      t(data.frame(c(mod="a",fit.a$summary$stat[paramcolname,"SD"]))),all=T) # posterior SDs (logit scale for p)
```

```
##      mod             alph0           alph[1] alph[2]           alph[3]
## 1     aa 0.131054999642846 0.948164920834027       0 0.270848944125131
## 2      a 0.101263885066988 0.762020890256065       0 0.205478894264039
##             alph[4]           alph[5]              gam0           gam[1]
## 1 0.166199641696339 0.191151677702176 0.713164206907022 3.16401019603604
## 2 0.132776533423446 0.154988847673038  18.2928813799646 30.8608175292399
##   gam[2]           gam[3]            gam[4]            gam[5]
## 1      0   2.228765827745 0.425779731123185 0.472929174843794
## 2      0 23.6394036347558  15.6856356428087  13.8386390322421
##                  tau             omega        logitpB[1]        logitpB[2]
## 1 0.0797875552311881 0.241212602070953 0.228211330665399 0.295386167780119
## 2 0.0993921574295562  8.08495106363356  0.20580862828897 0.229291890509749
##          logitpB[3]        logitpB[4]        logitpB[5]        logitpB[6]
## 1 0.292938279907323 0.353232425356599 0.259483181158245 0.271169009632412
## 2 0.242036825774491 0.315659889197197 0.229222677987904 0.240180041223643
##          logitpB[7]        logitpB[8]        logitpB[9]       logitpB[10]
## 1 0.210335650214183 0.310118216032376 0.294044676964387 0.371809397422306
## 2 0.187836687600068 0.239686003074795 0.253336862652271 0.322577115404581
##         logitpB[11]       logitpB[12]       logitpB[13]       logitpB[14]
## 1 0.217224445315031 0.236722795825226 0.224094088622657 0.329004052638496
## 2 0.188479461617128 0.199405466164408 0.194853866166504 0.259165549437167
##         logitpB[15]       logitpB[16]       logitpB[17]       logitpB[18]
## 1 0.619203833021752 0.276264558187381 0.218021631927631 0.238941082494046
## 2 0.423787671283938 0.231973989065891 0.189471749517864 0.182578523170196
##          logitpC[1]       logitpC[2]        logitpC[3]       logitpC[4]
## 1 0.567142189516534 0.81162903966783 0.971707526425215 0.75218596887055
## 2  24.7450126800535 30.0654432649749  28.8042617548124 32.5944218391275
##          logitpC[5]        logitpC[6]        logitpC[7]        logitpC[8]
## 1 0.808441417524929 0.659571861502625 0.587541756589929 0.894904227847862
## 2  22.9922748200995  30.8852011947961  24.4064301873278  32.5354941243877
##         logitpC[9]      logitpC[10]       logitpC[11]       logitpC[12]
## 1 1.04941811295494 3.06447357367835 0.557654055312638 0.701857494710011
## 2 25.7771866327534 42.9367790460796  23.1877760675723  22.9323729948747
##         logitpC[13]       logitpC[14]      logitpC[15]      logitpC[16]
## 1 0.523491020533135 0.806639539701885 6.30642076589738 1.65529233921366
## 2  35.3263660584325  32.9135940354897 63.8593419606833 27.1613687203872
##         logitpC[17]       logitpC[18]
## 1 0.635252020970344 0.557714179110597
## 2  25.5475093680281   37.948361540404
```

- Posterior median (original scale) and SD (logit scale) of each \(p^B\) are comparable between models, similarly for median and SD of each \(\alpha\) and \(\tau\)
  - to be expected because \(\boldsymbol{z}\) fully observed and modelled identically in all cases.
- Posterior median (original scale) for \(p^c\):
  - realistic model (`aa`): all are > 0.6, most are \(\approx\) 1,
  - hypothetical model (`a`, i.e. marginal model for \(\boldsymbol{t}\)): all are 1
  - => not enough spread for HIS-related regression to be useful (especially true for `a`).
- Posterior SD for `a` is
  - somewhat smaller for each \(p^B\) and \(\alpha\),
  - very similar for \(\tau\),
  - between 1 and 2 orders of magnitude larger for each logit(\(p^c\)), \(\gamma\), and \(\omega\).
- => hypothetical model slightly improves inference associated with \(\boldsymbol{z}\) but hugely sacrificing
  - inference associated with \(\boldsymbol{t}\),
  - predictive performance (larger DICs).

## Naive NIP.All estimates and their modeled counterparts

```
tmp <- data.frame( site[,c("siteLabNIP","siteLabNymphs")] )
rownames(tmp) <- site[,"siteName"]

pB.naive.ci95 <- 1.96*sqrt(tmp[,1]*(1-tmp[,1])/tmp[,2]) # half width only
pB.naive.ci95 <- data.frame(lo=tmp[,1]-pB.naive.ci95, hi=tmp[,1]+pB.naive.ci95)
pB.aa4.ci95 <- apply(pB.aa4, 2, quantile, prob=c(.025,.5,.975))

tmp <- data.frame( tmp, t(pB.aa4.ci95), pB.naive.ci95 )
tmp <- tmp[ order(tmp[,1], decreasing = TRUE), ]
tmp1 <- rownames(tmp)

par(las=2) # make label text perpendicular to axis
par(mar=c(9,5,5,2)) # increase x-axis margin
par(mgp=c(3,.4,0))
tmp2 <- barplot( tmp[,1], 
                 names.arg = paste(tmp1, "(", 
                                   format(tmp[,2], justify="right"),
                                   ")"),
                 col="gray95", ylab=expression(paste(p[B], " = NIP")), 
                 ylim=c(-0.08,.6) )
title("GRAY: naive NIP[All] estimate in desc. order and\nnaive 95% CI\nBLACK: modeled posterior median ('o') and\n95% credible interval")
text(tmp2[9], -.05, "site name (n)")

points(tmp2-.3, tmp[,6], pch="-", font=2, col="gray60")
points(tmp2-.3, tmp[,7], pch="-", font=2, col="gray60")

segments(tmp2-.3, tmp[,6], tmp2-.3, tmp[,7], lwd=2, col="gray60")

points(tmp2+.3, tmp[,4], pch="o", font=2)
points(tmp2+.3, tmp[,3], pch="-", font=2)
points(tmp2+.3, tmp[,5], pch="-", font=2)

segments(tmp2+.3, tmp[,3], tmp2+.3, tmp[,5], lwd=2)
```

## Naive NIP[HIS] estimates and their modeled counterparts

```
library(boot)

pC.aa4 <- inv.logit(scans.aa4.pooled[,19:36])  
pC.aa4.ci95 <- apply(pC.aa4, 2, quantile, prob=c(.025,.5,.975))
colnames(pC.aa4.ci95) <- rownames(tick2)

pC.naive.ci95 <- sapply(rownames(tick2),
              function(x){
                sum(subset(tick1, siteName==x)[,"his"])
              }) # HIS+ only

pC.naive.ci95 <- pC.naive.ci95 / tick2[,"lyme"] # naive pC est only

tmp <- data.frame(est=pC.naive.ci95, nh=tick2[,"lyme", drop=FALSE])
tmp
```

```
##                      est lyme
##           901  0.7500000    4
##           902  0.8571429    7
##           903  1.0000000    3
##           904  0.7142857    7
##           606  1.0000000    3
##           609  0.7500000    4
##           618  0.8333333    6
##           908  1.0000000    5
##           909  1.0000000    2
##           910        NaN    0
##           911  0.4285714    7
##           912  0.8571429    7
##           627  0.6666667    9
##           914  0.7500000    8
##           628  1.0000000    3
##           629  1.0000000    3
##           630  0.8333333    6
##           918  0.8421053   19
```

```
pC.naive.ci95 <- 1.96*sqrt(tmp[,1]*(1-tmp[,1])/tmp[,2]) # half width only
pC.naive.ci95 <- data.frame(est=tmp[,1], lo=tmp[,1]-pC.naive.ci95, hi=tmp[,1]+pC.naive.ci95)
rownames(pC.naive.ci95) <- rownames(tmp)

tmp <- data.frame( tmp, t(pC.aa4.ci95), pC.naive.ci95[,2:3] )
tmp <- tmp[ tmp1, ]

par(las=2) # make label text perpendicular to axis
par(mar=c(9,5,5,2)) # increase x-axis margin
par(mgp=c(3,.4,0))
tmp2 <- barplot( tmp[,1], 
                 names.arg = paste(tmp1, "(", 
                                   format(tmp[,2], justify="right"),
                                   ")"),
                 col="gray95", ylab=expression(paste(p[C], " = NIP[HIS]")), 
                 ylim=c(-0.1,1.1) )
#title("GRAY: naive NIP[HIS] estimate in desc. order and\nnaive 95% CI\nBLACK: modeled posterior median ('o') and\n95% credible interval")
title("GRAY: naive NIP[HIS] estimate\nBLACK: modeled posterior median ('o') and\n95% credible interval")
text(tmp2[9], -.04, "site name (y)")

points(tmp2, tmp[,4], pch="o", font=2)
points(tmp2, tmp[,3], pch="-", font=2)
points(tmp2, tmp[,5], pch="-", font=2)

segments(tmp2, tmp[,3], tmp2, tmp[,5], lwd=2)
```

## Naive DIN estimates and their modeled (posterior predictive) counterparts

```
library(boot)

pB.aa4 <- inv.logit(scans.aa4.pooled[,1:18])  
pB.aa4.ls <- as.list( as.data.frame(pB.aa4) )

library(parallel)

ystar.aa4 <- mcmapply( rbinom, 
          site$siteDenNymphsDragged, 
          pB.aa4.ls, 
          MoreArgs = list(n=length(pB.aa4.ls)),
          mc.cores=6)

din.aa4 <- t( t(ystar.aa4)/site$siteDenDragLen )
colnames(din.aa4) <- site$siteName
din.aa4.ci95 <- apply(din.aa4, 2, quantile, prob=c(.025,.5,.975))
```

```
tmp <- site[,c("siteDON.NIP","siteDenNymphsDragged")]
rownames(tmp) <- site[,"siteName"]

par(las=2) # make label text perpendicular to axis
par(mar=c(9,5,4,2)) # increase x-axis margin
par(mgp=c(3,.4,0))
tmp2 <- barplot( tmp[,1], 
                 names.arg = paste(tmp1, "(", 
                                   format(tmp[,2], justify="right"),
                                   ")"),
                 col="gray95", ylab=expression(paste(p[B]*m/a, " = DIN")),
                 ylim=c(-.005,.04))
title("GRAY: naive DIN estimate\nBLACK: modeled posterior median ('o') and\n95% 'predictive' interval")
text(tmp2[9], -.003, "site name (m)")

points(tmp2, din.aa4.ci95[2,tmp1], pch="o", font=2)
points(tmp2, din.aa4.ci95[1,tmp1], pch="-", font=2)
points(tmp2, din.aa4.ci95[3,tmp1], pch="-", font=2)

segments(tmp2, din.aa4.ci95[1,tmp1], tmp2, din.aa4.ci95[3,tmp1], lwd=2)
```

## Cross-validation via posterior prediction of \(\hat{p}^B\_{\text{med}}\)

```
pBhat <- site[order(site$siteLabNIP),c("siteLabNIP","siteName")] 
rownames(pBhat) <- pBhat[,2]

medpBhat <- mean(pBhat[9:10,])
medpBhat.site <- rownames(pBhat)[9:10]
medpBhat # observed median
```

```
## [1] 0.1994048
```

```
medpBhat.site # observed median site(s)
```

```
## [1]       901            908
```

```
library(parallel)

y.aa4 <- mcmapply( rbinom, 
          tick2$n, 
          pB.aa4.ls, 
          MoreArgs = list(n=nrow(pB.aa4)),
          mc.cores=6)

phat.aa4 <- t( t(y.aa4)/tick2$n )

find.med <- function(vect, rname, loc){
    ord <- order(vect)
    mid <- vect[ord][loc]
    md <- mean( mid )
    mdname <- rname[ord][loc]
    md <- data.frame( c(mid,md),
                      check.names=FALSE,
                      row.names=c(mdname,"median"))
    colnames(md) <- "pBhat"
    return(md)
}

phat.med.aa4 <- apply(phat.aa4, 1, 
                       find.med, 
                       rname=as.character(rownames(tick2)),
                       loc=c(9:10))
```

```
tmp <- sapply(
        phat.med.aa4,
        function(df){
          return(df[3,1])
        })

hist( tmp,
      main=paste("posterior predictive distr'n for median{naive NIP estimates}\nred = observed median (",
                 medpBhat.site[1], ",", medpBhat.site[2], ")"),
      cex.main=1, font.main=1,
      xlab=expression(paste("median ", hat(p)[B]))
)

abline(v=medpBhat, col="red")
```

```
sum( tmp > medpBhat ) / length(tmp) # 1-sided posterior predictive p
```

```
## [1] 0.224
```

```
quantile(tmp, prob=c(.05,.95)) # 90% predictive interval
```

```
##        5%       95% 
## 0.1281818 0.2283550
```

```
phat.med.aa4.site <- t( sapply(phat.med.aa4,
                                    function(x){ rownames(x)[1:2] },
                                    simplify="array" ))  
head(phat.med.aa4.site)
```

```
##             [,1]      [,2]            
## [1,] "      618" "    912"       
## [2,] "      901" "    902"    
## [3,] "      908" "    630"          
## [4,] "      912" "    911"       
## [5,] "      902" "    914"         
## [6,] "      606" "    908"
```

```
phat.med.aa4.site.tab <- apply(phat.med.aa4.site, 2, table)

phat.med.aa4.site9 <- sort(phat.med.aa4.site.tab[,1], decreasing=TRUE)
phat.med.aa4.site10 <- sort(phat.med.aa4.site.tab[,2], decreasing=TRUE)
```

```
tmp <- names(phat.med.aa4.site9)==medpBhat.site[1] 
tmp1 <- rep("grey",S)
tmp1[tmp] <- "red"

par(mfrow=c(2,1))
par(las=2) # make label text perpendicular to axis
par(mgp=c(3,1,0)) # default
par(mar=c(10,5,4,2)) # increase x-axis margin

barplot( phat.med.aa4.site9, col=tmp1 )
title("posterior predictive dist'n for site \nwith '9th largest' naive NIP estimate\nred = observed")

tmp <- names(phat.med.aa4.site10)==medpBhat.site[2] 
tmp1 <- rep("grey",S)
tmp1[tmp] <- "red"

barplot( phat.med.aa4.site10, col=tmp1 )
title("posterior predictive dist'n for site \nwith '10th largest' naive NIP estimate\nred = observed")
```

---
